# Comprehensive safety evaluation of *Withania somnifera* (Ashwagandha): an AI-driven meta-analysis and quantitative structure–activity relationship based toxicity assessment

**DOI:** 10.3389/fnut.2025.1658265

**Published:** 2025-11-24

**Authors:** Yotam Ronen, Coralie Ebert, Bat-Chen Tamim-Yecheskel, Shani Zev, Ophir Kantor, Hilla Ben-Hamo Arbel

**Affiliations:** MeNow Ltd., Ramat-Gan, Israel

**Keywords:** *Withania somnifera*, Ashwagandha, toxicity, meta-analysis, QSAR

## Abstract

**Objective:**

This study evaluates the safety profiles of *Withania somnifera* (Ashwagandha), an adaptogenic herb prevalent in Ayurvedic medicine, focusing on liver and reproductive toxicity. Utilizing advanced AI methodologies, we conducted a comprehensive meta-data analysis to assess the safety of the plant’s root and non-root parts, comparing Ashwagandha’s safety to other herbal supplements.

**Methods:**

We employed natural language processing (NLP) to systematically review existing literature and utilized quantitative structure–activity relationship (QSAR) models to predict liver and reproductive toxicity at the molecular level. Special attention was given to withanolides, the bioactive compounds in Ashwagandha, due to conflicting safety information. Additionally, we reviewed case studies reporting liver toxicity, noting that many involved supplements containing both leaves and roots, complicating the identification of the toxicity source.

**Results:**

Our analysis indicated that Ashwagandha root exhibits a superior safety profile compared to non-root parts, particularly concerning liver and reproductive toxicity. When compared to a broad set of other herbal supplements, Ashwagandha root was found to have a better safety profile than most, making it a first-choice ingredient for safe and effective use in supplements. While non-root parts of Ashwagandha showed higher toxicity potential than the root, their safety profile was still comparable to other edible plants and herbal supplements.

**Conclusion:**

This study suggests that the root of *Withania somnifera* (Ashwagandha) demonstrates a favorable safety profile, particularly concerning liver and reproductive toxicity, when compared to other herbal supplements. Our findings support the traditional preference for root-based formulations and highlight the importance of distinguishing between plant parts in safety assessments. While these results strengthen the evidence supporting the safe use of Ashwagandha root, further experimental and clinical validation would be valuable to confirm these AI-driven predictions and literature-based findings. The study also illustrates how artificial intelligence approaches can complement traditional toxicological evaluations and enhance safety assessment frameworks in the herbal supplement industry.

## Introduction

*Withania somnifera* (Ashwagandha) is a medicinal plant widely used in Ayurvedic practice for its adaptogenic properties, including stress reduction, cognitive support, and enhancement of overall vitality (1–3). Among its various plant parts, the root has been traditionally preferred and is the main source of modern dietary supplements designed to promote relaxation and well-being (2, 3).

The biological activity of Ashwagandha is largely attributed to withanolides, a class of steroidal lactones with reported anti-inflammatory, neuroprotective, and cytotoxic effects (2, 4). However, safety data on these compounds remain inconsistent. Some studies describe hepatoprotective and beneficial effects (5, 6), whereas others have raised safety concerns regarding Ashwagandha, particularly its potential hepatotoxicity. Studies have suggested that withanone may induce hepatic DNA damage under certain conditions (4), while case series from India reported liver injury associated with multi-ingredient or non-root formulations (7). A subsequent review emphasized the need for standardization and monitoring of Ashwagandha products to mitigate such risks (8). These findings highlight the importance of distinguishing between root and non-root preparations in safety evaluations. These discrepancies highlight the need for a more systematic evaluation of Ashwagandha’s safety, particularly regarding differences between the root and non-root parts and the specific molecular constituents involved.

Recently, interest in the leaves of Ashwagandha has grown, as they have been reported to exhibit additional biological activities such as anti-inflammatory and immunomodulatory effects (9). However, as the use of Ashwagandha supplements expands, concerns have emerged regarding the safety of both the root and the leaves, particularly in terms of liver and reproductive toxicity. Several case studies have documented liver injury linked to Ashwagandha supplementation (4), but a key challenge in these reports is that many supplements contain a mixture of leaves and roots, making it difficult to determine which part of the plant, if any, is responsible for the toxicity. This highlights the need for a more rigorous examination of the specific parts of the plant.

To address these concerns, this study aims to conduct a comprehensive meta-data analysis of Ashwagandha’s safety, focusing on the liver and reproductive toxicity of the molecules present in both the root and the leaves. Using advanced AI methodologies such as natural language processing (NLP) and QSAR-based toxicity predictions, we seek to identify potential toxic molecules and differentiate the safety profiles of the plant’s parts. The goal of this study is to provide evidence-based guidance on the safest use of Ashwagandha, helping inform both the herbal supplement industry and future research.

## Materials and methods

In this safety evaluation of Ashwagandha, we utilized a combination of advanced AI techniques and predictive modeling to provide a comprehensive assessment. Our approach integrated both literature-based analysis and molecular-level toxicity predictions to ensure a robust and multidimensional understanding of Ashwagandha’s safety profile.

The analysis was conducted in two major parts. First, we applied state-of-the-art Natural Language Processing (NLP) models to analyze scientific literature. This allowed us to assess the safety and toxicity of Ashwagandha by extracting relevant data from research abstracts. We evaluated three NLP systems, including a publicly available transformer-based model (SciBERT) and two commercially available large language models (OpenAI, USA) (Supplementary Table 1).

In addition, we used two separate Quantitative Structure–Activity Relationship (QSAR) models to predict the potential toxicity of chemical constituents found in Ashwagandha. These models were specifically tailored to analyze the molecular composition, identifying any risks associated with compounds such as mutagenicity or carcinogenicity. The analysis focused specifically on the root and leaves, as these are the primary plant parts used in commercial herbal supplements and reported in safety case studies, allowing for a more targeted evaluation of their toxicological profiles.

To refine our molecular analysis, we developed a predictive model capable of identifying the plant part (root or leaves) in which each molecule is most likely to occur. This model enabled us to more accurately attribute individual compounds to the corresponding plant part and, consequently, to perform a more precise QSAR-based toxicity evaluation of the root and leaves.

In parallel, we constructed a comparison dataset comprising edible plants and commonly used herbal supplements, which served as a reference framework for assessing the relative safety of *Withania somnifera*.

By integrating these complementary approaches—literature-based NLP analysis, molecular prediction, and QSAR modeling—we generated a comprehensive and comparative toxicological assessment of Ashwagandha, distinguishing between the root and aerial parts. The specific methodologies are described in detail in the following sections.

### Meta-analysis using NLP models

To evaluate the safety of Ashwagandha and compare it with other herbal extracts, we tested three different Natural Language Processing (NLP) models for their effectiveness in predicting the safety and toxicity of herbs based on scientific literature. After comparative testing on a diverse set of plant-related abstracts, the GPT-4 model demonstrated the highest accuracy and consistency in toxicity prediction and was therefore selected for the subsequent analyses.

#### Model testing and selection process

**Figure 1:**
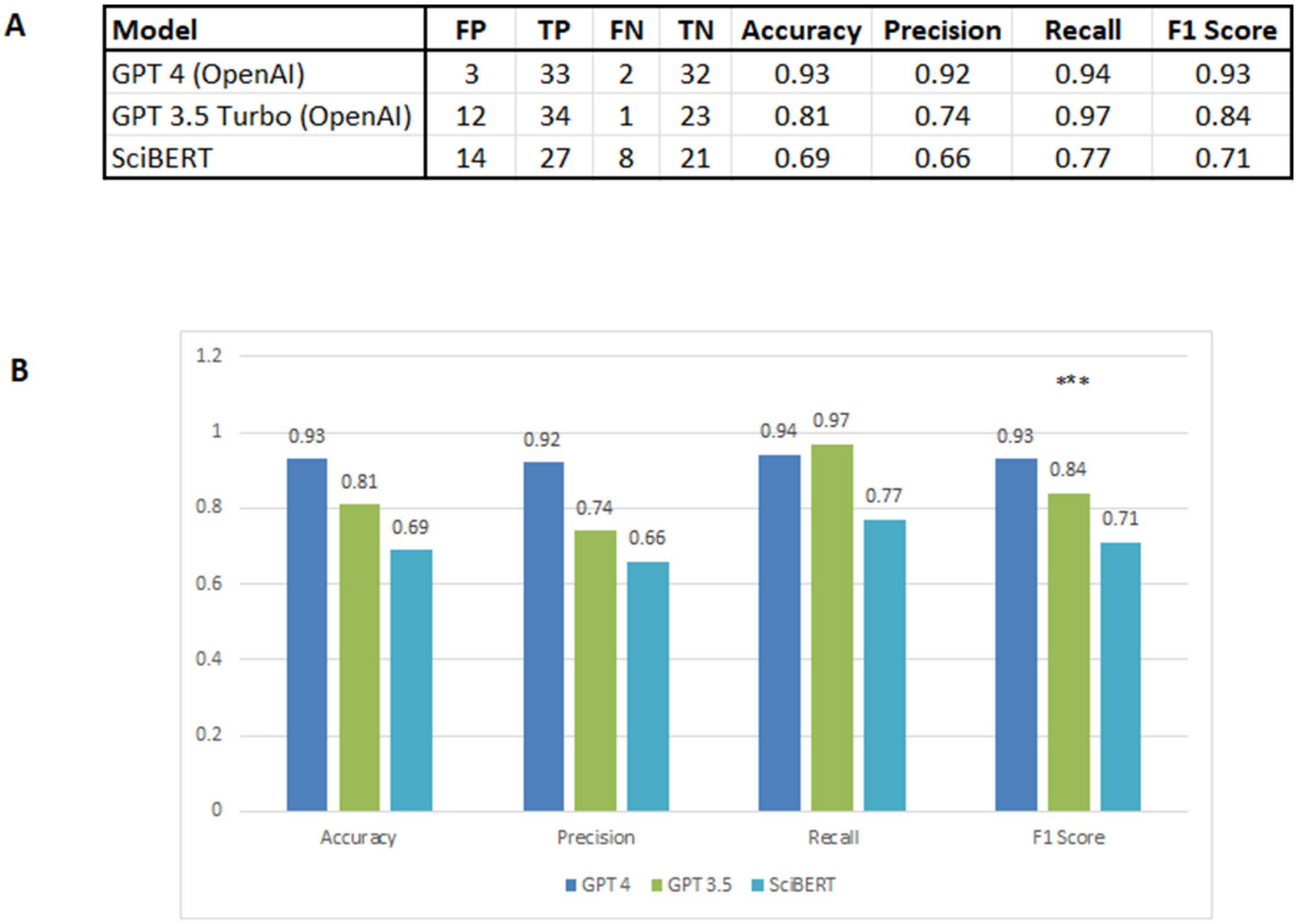
Performance evaluation of three NLP models demonstrates that GPT-4 outperformed GPT-3.5 Turbo and SciBERT. **(A)** Performance comparison of three NLP models—GPT-3.5 Turbo, GPT-4, and SciBERT—evaluated on a validation dataset of 70 articles. Models were assessed based on their ability to accurately predict mentions of toxicity in the literature. Performance metrics include FP (False Positive), TP (True Positive), FN (False Negative), TN (True Negative), accuracy, precision, F-score, and recall **(B)** Histogram comparing the performance of GPT-3.5 Turbo, GPT-4, and SciBERT across four key metrics: accuracy, precision, F-score, and recall. The figure illustrates the relative strengths of each model in identifying toxicological information within the validation dataset. GPT-4 demonstrates superior performance across most metrics, followed by GPT-3.5 Turbo, whereas SciBERT shows comparatively lower performance.

To establish a reliable baseline for model performance, we manually compiled a dataset of 70 peer-reviewed articles from PubMed describing toxicological assessments of various plants, encompassing both known toxic and non-toxic herbs. The dataset was balanced, with approximately half of the articles reporting toxicity concerns (Supplementary Table 2). This balanced dataset was used to evaluate the ability of different Natural Language Processing (NLP) models to distinguish between toxic and safe herbal extracts based on scientific text (Figure 1).

We selected three transformer-based NLP models—GPT-3.5 Turbo, GPT-4, and SciBERT—to represent a spectrum of architectures with varying training corpora, parameter scales, and domain specializations:

- GPT-3.5 Turbo (OpenAI) was chosen as a representative large language model (LLM) optimized for speed and efficiency. Although it is a general-purpose model, it has demonstrated solid performance on natural language classification tasks and serves as a useful baseline for comparison against more advanced or domain-specific models.
- GPT-4 (OpenAI) was included as a state-of-the-art LLM with enhanced contextual reasoning, multi-step inference, and improved handling of complex scientific syntax. Its broader and more diverse training corpus enables superior semantic understanding and reduction of hallucination in evidence-based classification tasks. GPT-4 therefore represents the most advanced general-purpose NLP approach currently available for scientific text interpretation.
- SciBERT, developed by the Allen Institute for AI, was selected as a domain-specific model trained on 1.14 million scientific papers from Semantic Scholar. As a BERT-derived architecture, it is specifically designed to understand technical terminology, sentence structures, and context typical of scientific publications, making it an appropriate comparator for assessing whether domain-specific pretraining confers advantages over general-purpose LLMs.

Each model was prompted and evaluated on the same dataset using standardized input prompts to predict whether an abstract described a toxic or non-toxic plant extract. Model performance was assessed across four metrics: accuracy, precision, recall, and F-score.

Among the tested models, GPT-4 achieved the best overall performance, showing superior classification accuracy and balanced precision–recall trade-offs (Figure 1A). In contrast, GPT-3.5 Turbo demonstrated strong recall but lower precision, tending to over predict toxicity, while SciBERT exhibited good recognition of domain-specific terminology but lower generalization across diverse abstracts.

Statistical comparisons between model outputs were conducted using bootstrap confidence intervals for each performance metric. GPT-4 showed significantly higher accuracy and F-score compared to SciBERT (Supplementary Table 3), whereas other pairwise differences were not statistically significant.

Based on its consistent superiority across all evaluation criteria, GPT-4 was selected as the primary NLP model for the subsequent literature-based safety assessment of *Withania somnifera*. Its capacity to accurately interpret scientific context and distinguish subtle toxicological signals ensured a robust and reliable foundation for the meta-analysis of Ashwagandha’s safety profile (Figure 1B).

### Toxicity prediction based on molecular structure (SMILES)

To predict the potential toxicity of molecules in Ashwagandha, we employed a Quantitative Structure–Activity Relationship (QSAR) model based on molecular structure data, specifically using Simplified Molecular Input Line Entry System (SMILES) representations. The goal was to assess the toxicity of each molecule by predicting its biological activity and linking it to potential toxic outcomes.

#### Training dataset and model development

For training the model, we utilized a dataset of molecules known to have potential toxic effects, as reported in the SideR database.^1^ This dataset provided a comprehensive resource of chemically diverse compounds with documented toxicity profiles, enabling the model to learn which molecular features are indicative of toxicity.

In addition to the SideR data, we trained the model on a broader dataset of 300,000 molecules, which included information on their interactions with 1,667 different molecular targets. These targets span a wide range of biological pathways and are relevant to the key toxicological endpoints we aimed to predict, such as mutagenicity, hepatotoxicity, and carcinogenicity.

The QSAR model used for predicting toxicity was developed internally for this study using the RandomForestClassifier implementation from the *scikit-learn* (sklearn) package (Supplementary Table 1). Random forests are ensemble learning algorithms that build multiple decision trees during training and combine their outputs to improve predictive accuracy and reduce the risk of overfitting. In this context, each decision tree learns to classify molecular structures according to their predicted toxicological outcomes, and the aggregated “majority vote” of the forest provides a robust final prediction. This approach is particularly well suited for QSAR tasks, as it can handle high-dimensional molecular descriptor data, capture nonlinear relationships between features and biological activity, and provide measures of feature importance that help interpret the drivers of predicted toxicity.

#### Prediction process and model accuracy

Using this extensive training dataset, the QSAR model predicted the biological activities of each molecule found in Ashwagandha based on their structural features. By analyzing these predicted activities, we were able to infer potential toxic effects, identifying compounds that might pose risks due to their biological target interactions.

**Figure 2:**
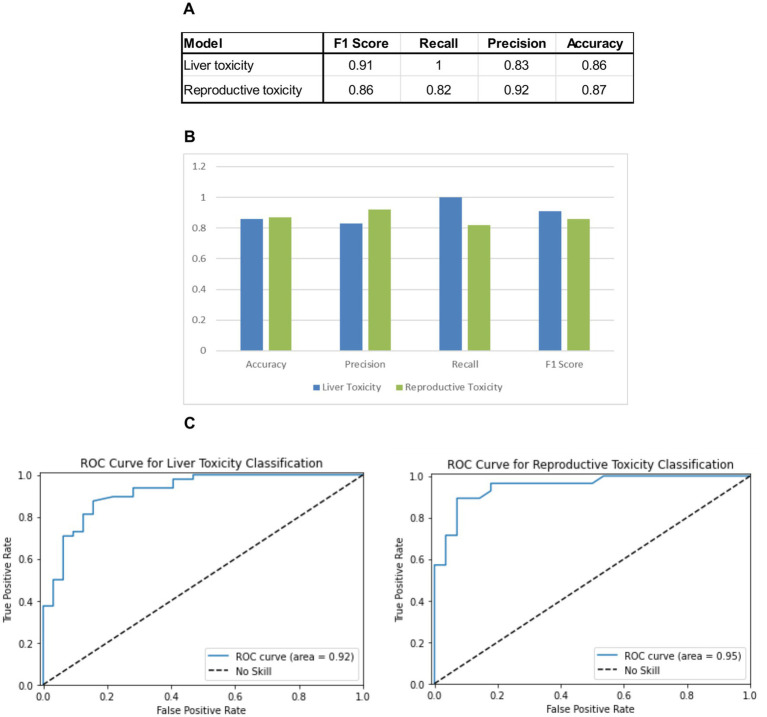
Performance evaluation of QSAR models for liver and reproductive toxicity. **(A)** Summary of the performance of key metrics, including accuracy, precision, F-score, and recall, used to evaluate each model’s ability to predict the toxicity of molecules. **(B)** Histogram comparing the performance of two QSAR toxicity models—liver toxicity and reproductive toxicity—across key metrics: accuracy, precision, F-score, and recall. **(C)** ROC curves for the two QSAR toxicity models—liver toxicity (left) and reproductive toxicity (right). Each curve plots the true positive rate (sensitivity) against the false positive rate (1-specificity) at various threshold settings, illustrating the models’ ability to distinguish between toxic and non-toxic molecules. The area under the curve (AUC) indicates the models’ performance, with higher AUC values representing stronger discriminatory power for predicting liver and reproductive toxicity.

The model achieved a high accuracy in predicting the toxicity of molecules based on their structure, demonstrating its effectiveness in identifying toxicological risks, for both predicting liver toxicity and reproductive toxicity (fetal, hormonal and thyroid related toxicity) (Figures 2A–C). This high level of accuracy was crucial for ensuring that the toxic predictions were reliable and actionable in the context of Ashwagandha’s safety evaluation.

### Predicting the part of the plant for molecules

While the root of Ashwagandha is commonly used for medicinal purposes, there are also cases where other parts of the plant, such as the leaves, are used, despite associated safety concerns. To better compare the safety profiles of molecules found in the root versus other parts of Ashwagandha, we developed a predictive model that classifies whether a molecule is primarily present in the root or elsewhere based on its SMILES representation.

#### Training dataset and model development

We trained the model on a dataset comprising over 600 data points from more than 50 different plants. Each molecule was labeled according to the plant part in which it is predominantly found, such as the root, leaf, stem, or flower. The molecular structures of these compounds, encoded in SMILES format, were used as input for the model.

This diverse dataset enabled the model to learn structural and chemical patterns associated with the molecular composition of different plant organs. As a result, it could predict with high confidence the most probable localization of a given compound within the plant—particularly distinguishing molecules characteristic of the root from those predominantly found in aerial parts. This predictive capacity improved the precision of the subsequent QSAR analysis by allowing toxicity assessment to be specifically attributed to root- or leaf-derived molecules. A similar approach as the toxicity’s QSAR model was used for the prediction of plant parts, based on random forest (Supplementary Table 1).

#### Model accuracy and application

**Figure 3:**
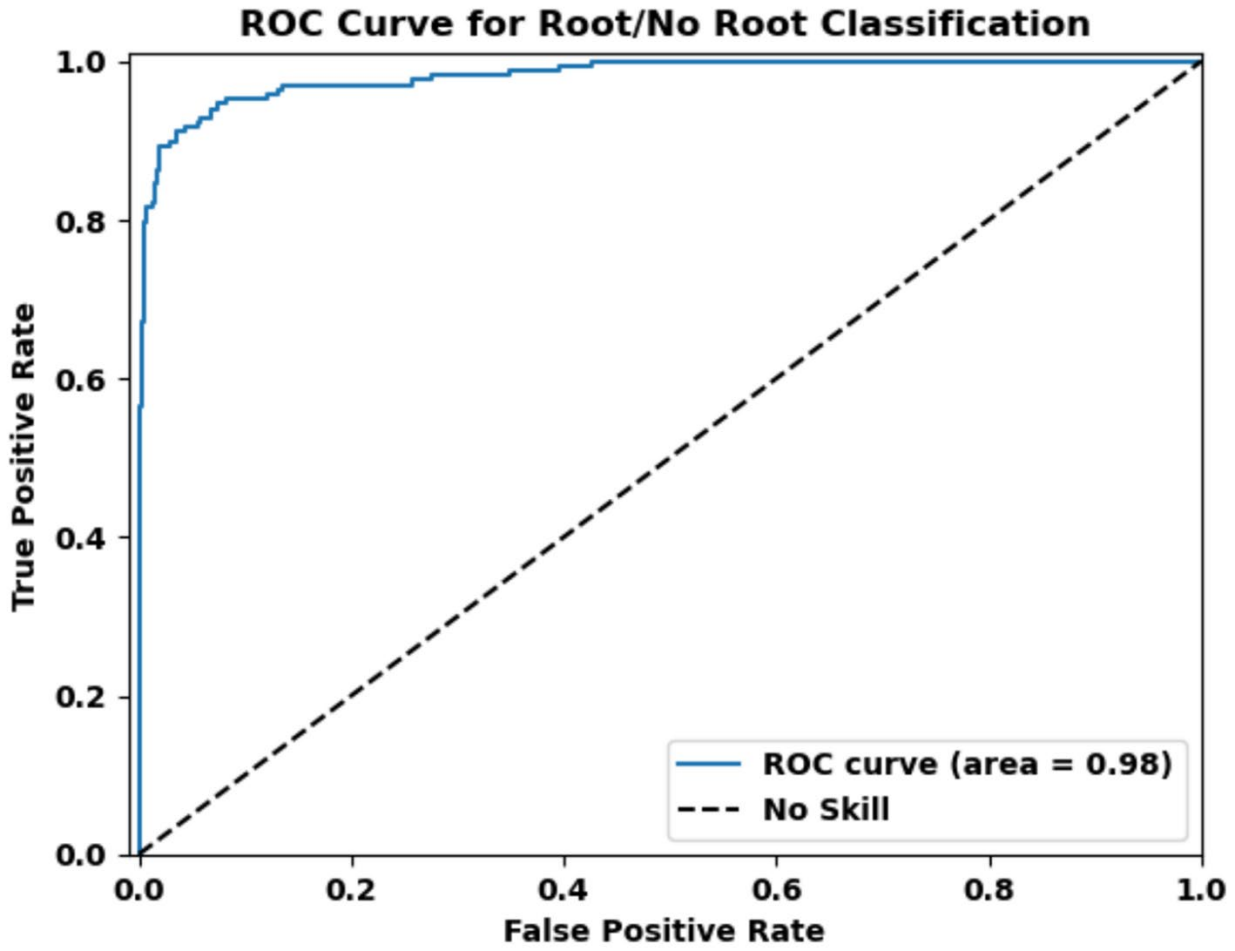
ROC curve for the model classifying molecules likely to be present in plant roots. The curve plots the true positive rate (sensitivity) against the false positive rate (1-specificity) at various thresholds. The area under the curve (AUC) reflects the model’s performance in distinguishing between molecules that are predicted to be present in the root versus other parts of the plant, with higher AUC values indicating better classification accuracy.

The model achieved an accuracy of 0.84 in classifying molecules based on their part of origin and ROC AUC of 0.98 (Figure 3). The high ROC AUC value indicates excellent sensitivity and specificity, which translates into a very low number of false positives—i.e., compounds incorrectly classified as root-derived. This low error rate is critical for downstream analyses, as it reduces the likelihood of misattributing molecular origin and strengthens the reliability of the biological interpretations. This strong performance gave us confidence in the model’s ability to distinguish root-specific compounds from those found in other plant parts.

By applying this model to Ashwagandha, we were able to refine our safety analysis by identifying the specific molecular profiles of the root versus the rest of the plant. This enabled a more detailed and accurate comparison, especially in cases where the leaves might be used despite documented safety concerns.

#### Computation of a toxicity score based on the molecular composition

To facilitate the comparison of the toxicity profiles between the different plant parts of Ashwagandha and a dataset of edible plants and herbal supplements, we developed a toxicity score. This score was calculated based on the predicted toxicity of each molecule in the specific plant part or in the whole plant depending on the scope of analysis. Specifically, the toxicity score was computed as the sum of the toxicity confidence values for all molecules present in the plant, divided by the total number of molecules. This approach provided an aggregate measure of toxicity, allowing for a direct comparison between Ashwagandha and other plants in terms of their overall toxicity risk. The score helped standardize the evaluation, enabling us to quantify the safety profile of Ashwagandha in the context of the broader dataset.

### Comparison to edible plants and herbal supplements

To contextualize the results of both our NLP literature analysis and the toxicity predictions for Ashwagandha, we compared them to a set of edible plants and herbal supplements. This comparison was made possible through the MeNow database,^2^ a proprietary resource that contains detailed information on the molecular composition of over 60,000 organisms, including approximately 43,000 plants. The database also includes valuable data on plant edibility and traditional uses, allowing us to perform a meaningful benchmark analysis.

#### Selection of edible plants

In the MeNow database, there are 2,766 plants classified as edible. To ensure a fair comparison with Ashwagandha, we selected edible plants that had a similar number of active molecules to those found in Ashwagandha. According to the MeNow database, Ashwagandha contains 338 unique molecules. For our comparison, we chose edible plants that have between 100 and 500 active secondary metabolites appearing in the MeNow database, resulting in a selection of 676 plants that matched this criterion.

By narrowing the comparison to plants with a similar molecular complexity, we ensured that the differences in safety profiles were not simply a reflection of the number of molecules present, but rather of their specific biological activities and potential toxicities.

#### Comparison to herbal supplements

In addition to edible plants, we compared Ashwagandha’s safety profile to that of other well-known herbal supplements. To compile a relevant dataset, we referenced a published list of herbal supplements for which detailed molecular composition data is available in the MeNow database (8). From this list, we identified 228 herbal supplements to include in our comparison dataset.

This comparative approach enabled us to evaluate Ashwagandha’s safety within the broader context of herbal medicine. By comparing its molecular and safety profiles with those of other edible plants and widely used supplements, we could assess Ashwagandha’s relative safety and identify any unique risks linked to its specific molecular composition.

## Results

### NLP analysis results demonstrate of a high safety profile of Ashwagandha

#### NLP analysis of articles mentioning Ashwagandha identified low number of articles with toxicity concerns

We collected a total of 1,402 publications mentioning Ashwagandha from PubMed, covering the period from 1958 to August 2024. Out of these, six publications were withdrawn by the authors and were excluded from the subsequent text analysis. The remaining 1,396 publications formed the basis of our analysis. The dataset comprised 1,196 journal articles, including both comparative and evaluation studies; 183 reviews that summarized the findings of prior research on WS; 18 clinical trials and case reports, providing firsthand data on the effects of WS in human subjects; Five short news and letters offering brief insights or announcements related to WS. To maintain a high level of scientific rigor, we limited the scope of our analysis to articles indexed in PubMed, ensuring that the data came from sources with recognized scientific accuracy. The NLP analysis was performed using a large language model (LLM)–based system (GPT-4), employing the same prompts that were used during model selection. For cases where the model predicted potential toxicity, an additional set of prompts was applied to classify the type of toxicity (Supplementary Table 1).

**Figure 4:**
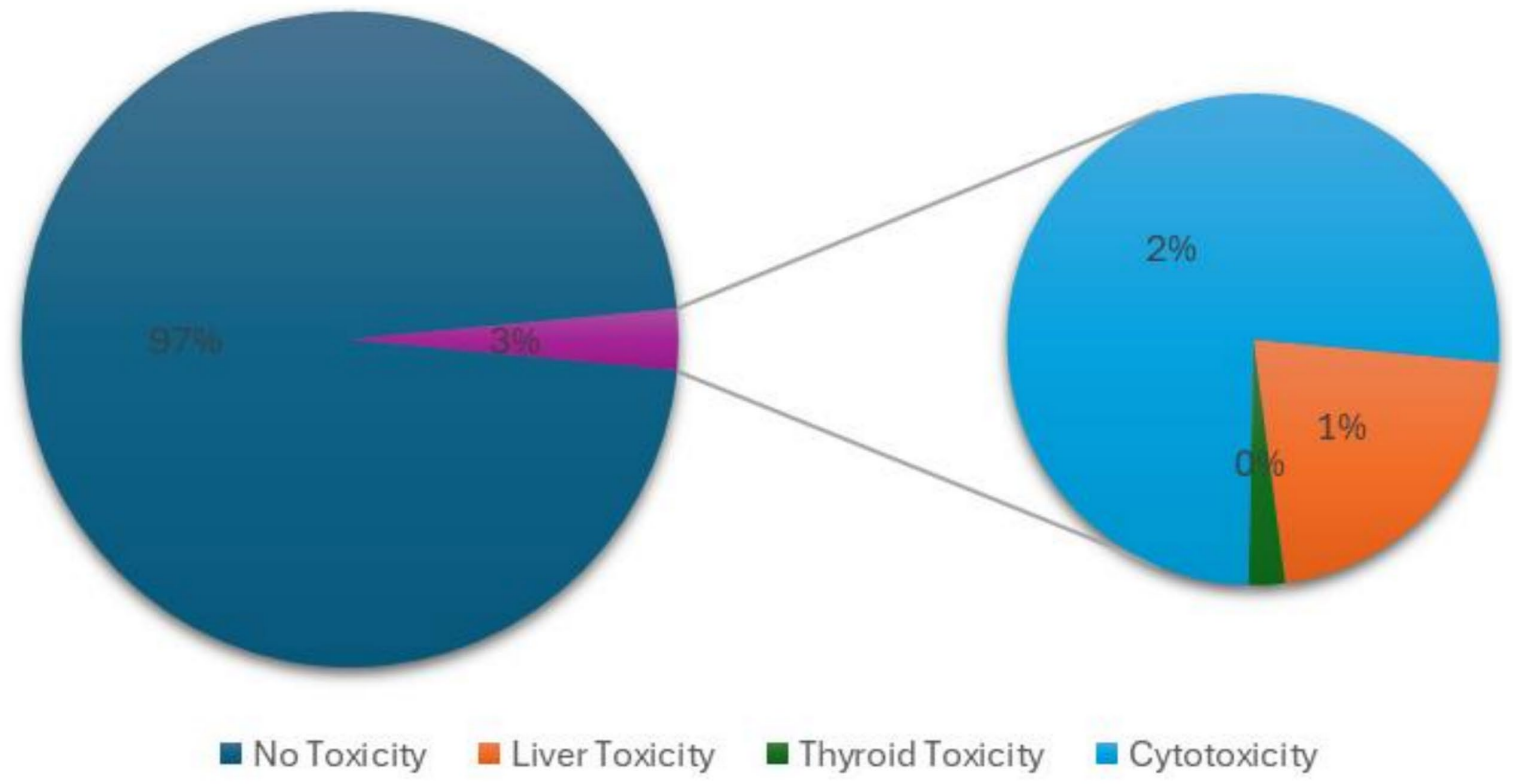
Low proportion of articles raise toxicity concerns for *Withania somnifera* (Ashwagandha). According to the NLP analysis of 1,396 articles on the plant’s safety, 97% reported no safety concerns, while 3% indicated some level of toxicity. Of these, 2% focused on cytotoxicity, primarily related to anti-cancer activity, and 1% discussed liver toxicity.

According to the NLP analysis, 97% of the articles did not mention any toxic concerns for Ashwagandha (Figure 4). Of the 37 articles that did address potential toxicity, 32 articles dealt with cytotoxicity, specifically targeting cancerous cells; Nine articles mentioned potential liver toxicity, with five of these also discussing cytotoxicity; One article referred to a possible adverse effect on thyroid activity; No articles mentioned concerns for fetal toxicity and endocrine disruption. These findings suggest that while toxicity concerns for Ashwagandha are rare, the most frequently mentioned toxic effects relate to cytotoxicity in cancer treatment contexts, with some reports of liver toxicity and very limited mention of thyroid concerns (Supplementary Table 4).

#### Cytotoxicity-related articles are mostly linked to anti-cancer activity

As part of the NLP analysis, 32 publications specifically reported cytotoxic effects of Ashwagandha (Supplementary Table 4). Importantly, the majority of these studies focused on cytotoxicity in the context of cancer research, highlighting the potential of Ashwagandha and its bioactive compounds as anti-cancer agents rather than indicating general toxicity.

Several studies detailed selective cytotoxic activity against various cancer cell lines. For example, Halder et al. and Iguchi et al. demonstrated cytotoxic effects against melanoma and gingival carcinoma cells, respectively (1, 10), while Yadav et al. reported growth inhibition across five cancer cell lines (11). Pretorius et al. observed that Ashwagandha extracts were non-toxic at low concentrations but could exert cytotoxic effects at higher doses, particularly against MRC-5 cells (12). Other studies, including Liu et al. and Choudhary et al., highlighted cytotoxicity against lung cancer cells (13, 14), and Senthil et al. reported anticancer activity of leaf extracts against human gastric adenocarcinoma cells (15). Similarly, Siddique et al. described strong cytotoxic activity against liver and breast cancer cell lines (16), and Al-Fatimi et al. reported notable anti-breast cancer effects of a fungus growing on Ashwagandha root (17).

The cytotoxic effects are largely attributed to withanolides, particularly Withaferin A, a key bioactive compound. Studies by Devi et al., Xia et al., and Misra et al. demonstrated Withaferin A’s capacity to induce apoptosis and enhance cancer therapy (18–20). Mechanistic investigations, including work by Jilani et al. and Lv & Wang, revealed disruption of cancer cell division and inhibition of G2/M checkpoint proteins, further supporting selective cytotoxicity toward malignant cells (21–23). Comprehensive reviews (Kumar et al., Albahri et al.) provide an overarching summary of Ashwagandha’s anti-cancer potential (24, 25).

In summary, cytotoxicity observed in these studies is largely targeted to cancerous cells and does not indicate safety concerns for general consumption of Ashwagandha. Rather, these findings underscore its therapeutic potential in oncology.

#### Liver toxicity reports are rare and context-dependent

Our NLP analysis identified nine publications reporting potential liver toxicity associated with Ashwagandha (Supplementary Table 4). While Ashwagandha is generally considered safe, these studies highlight rare, context-specific cases of hepatotoxicity. Many reports involved confounding factors such as pre-existing liver conditions, concomitant use of other supplements, or high dosages, limiting the generalizability of these findings.

For example, case series from India indicate that liver injury following Ashwagandha use is uncommon and often occurs in individuals with underlying health conditions or concurrent supplement intake (4). Another study noted that improper preparation or excessive consumption could contribute to hepatocellular stress, but routine use remains low-risk (26). Systematic reviews of herb-induced liver injury (HILI) similarly classify Ashwagandha-related hepatotoxicity as rare and context-dependent (27).

Clinical monitoring studies observed only mild, reversible liver enzyme elevations in a small number of participants taking Ashwagandha root extracts, which normalized upon discontinuation (28). Animal studies indicate that liver damage occurs only at extremely high doses, far above typical human consumption (29). Individual case reports, including instances in healthy adults, suggest that hepatotoxicity is uncommon and generally reversible (30–32).

Overall, the literature indicates that while isolated liver toxicity cases exist, Ashwagandha’s hepatotoxic risk for the general population at recommended dosages is minimal. These findings reinforce the importance of proper dosing and consideration of pre-existing conditions in supplement use.

#### Only one isolated case of thyroid toxicity was reported

Out of nearly 1,400 PubMed-indexed articles analyzed in our NLP study, only one article raised concerns about thyroid toxicity linked to Ashwagandha. This study, *Thyroid Dysfunction Induced by Ashwagandha: A Case Review*, detailed a single case of thyroid dysfunction in an individual who developed abnormal thyroid activity after taking Ashwagandha supplements (33). The symptoms subsided once the individual stopped taking the supplement, suggesting a potential link between Ashwagandha and thyroid function in this particular case.

However, it is important to note that this study represents just one isolated case report out of nearly 1,400 articles. Given that none of the other analyzed publications mentioned thyroid toxicity, the evidence for a widespread concern is virtually negligible. The case highlighted in the study involved a unique scenario, and the authors themselves acknowledged the need for more research to determine whether the findings are an anomaly or indicative of a broader risk.

Considering the vast body of research that does not report any thyroid-related issues, the likelihood of Ashwagandha causing thyroid toxicity appears to be extremely low, particularly for individuals without pre-existing thyroid conditions.

#### Comparative toxicity analysis demonstrate that Ashwagandha has similar or even lower toxicity reports than other edible plants and herbal supplements

Since it is challenging to draw definitive conclusions solely from isolated reports, we expanded our analysis by comparing the literature on Ashwagandha’s toxicity with other edible plants and herbal supplements. For this purpose, we prompted the NLP models on a dataset of 331 edible plants and 171 herbal supplements. This dataset was based on the larger pool presented in the materials and methods section, containing 676 edible plants and 227 herbal supplements, but was further restricted to botanicals with sufficient literature to support a robust analysis.

**Figure 5:**
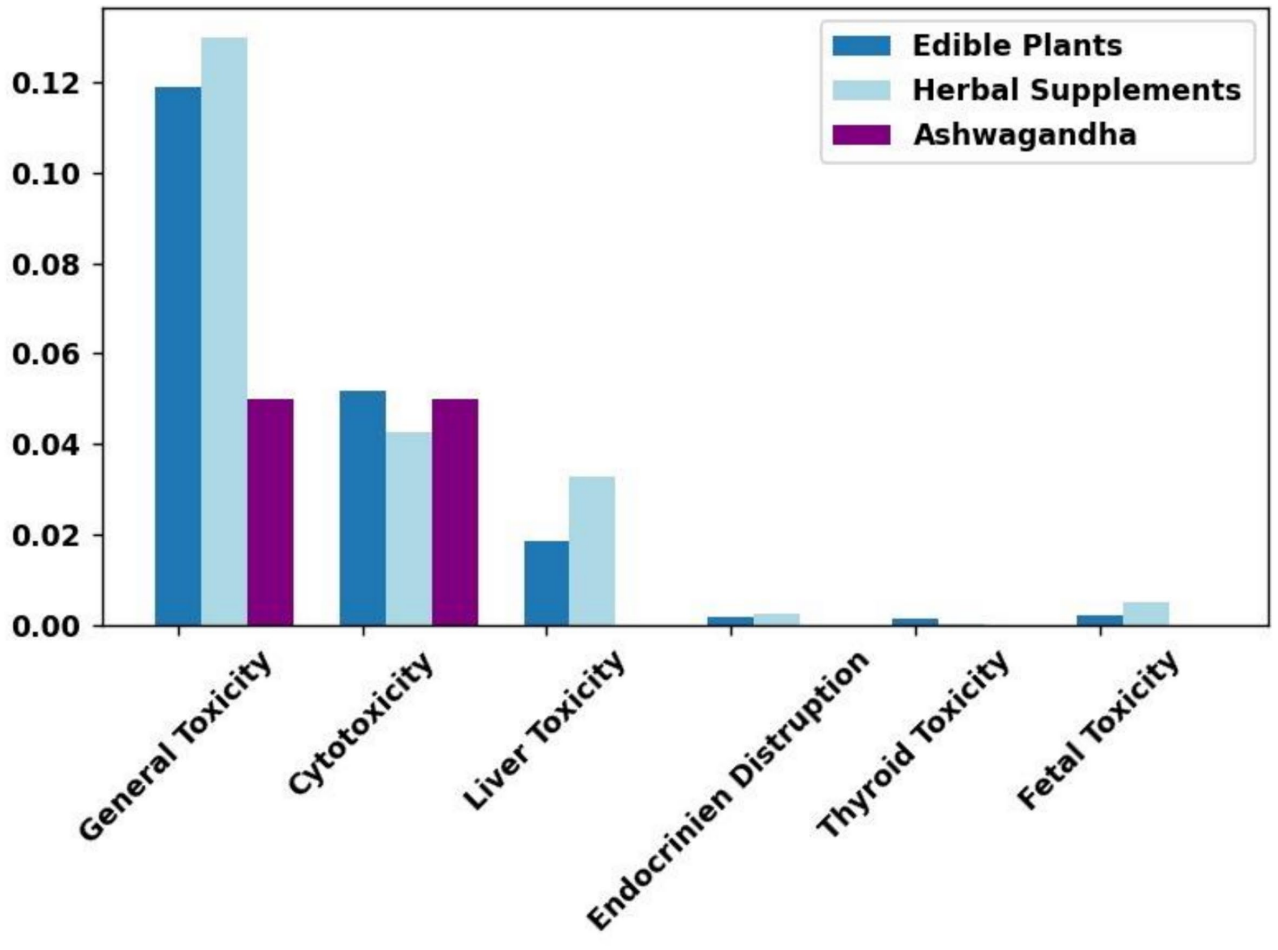
*Withania somnifera* (Ashwagandha) has similar or even lower toxicity reports than other edible plants and herbal supplements. A meta-data NLP analysis of the literature, comparing the frequency of toxicity concerns for Ashwagandha to two datasets: 331 edible plants and 171 herbal supplements. As shown, various types of toxicity were examined, including general toxicity, cytotoxicity, liver toxicity, endocrine disruption, thyroid toxicity, and fetal toxicity. For all toxicity types except cytotoxicity (mainly linked to anti-cancer activity), Ashwagandha performed better than the average edible plant or herbal supplement. Cytotoxicity results for Ashwagandha were similar to the average levels observed in the edible plant and herbal supplement datasets.

For each plant, the NLP models analyzed articles for various toxicity parameters, including general toxicity (including cytotoxicity), cytotoxicity, liver toxicity, endocrine disruption, thyroid toxicity, and fetal toxicity. For each toxicity type, we computed an average score for each plant. The results showed that Ashwagandha was below the average of both edible plants and herbal supplements across all categories of toxicity, except for cytotoxicity (Figure 5).

In terms of cytotoxicity, Ashwagandha’s results fell between the averages for edible plants and herbal supplements, indicating that its cytotoxicity is not unusually high but rather consistent with its known use in anti-cancer contexts. Importantly, none of these differences were statistically significant.

Additionally, a similar study presented in the article *Herbal Supplements and Hepatotoxicity* reviewed various herbal supplements for Herbal-Induced Liver Injury (HILI) (34). The review was conducted by expert reviewers, who categorized supplements as hepatotoxic based on the thoroughness of case reports. These reports had to include specific details such as serum biochemistry, liver biopsy findings, and well-documented clinical courses of injury. In their analysis, Ashwagandha was classified as negative for HILI, meaning no cases met the criteria for hepatotoxicity.

**Figure 6:**
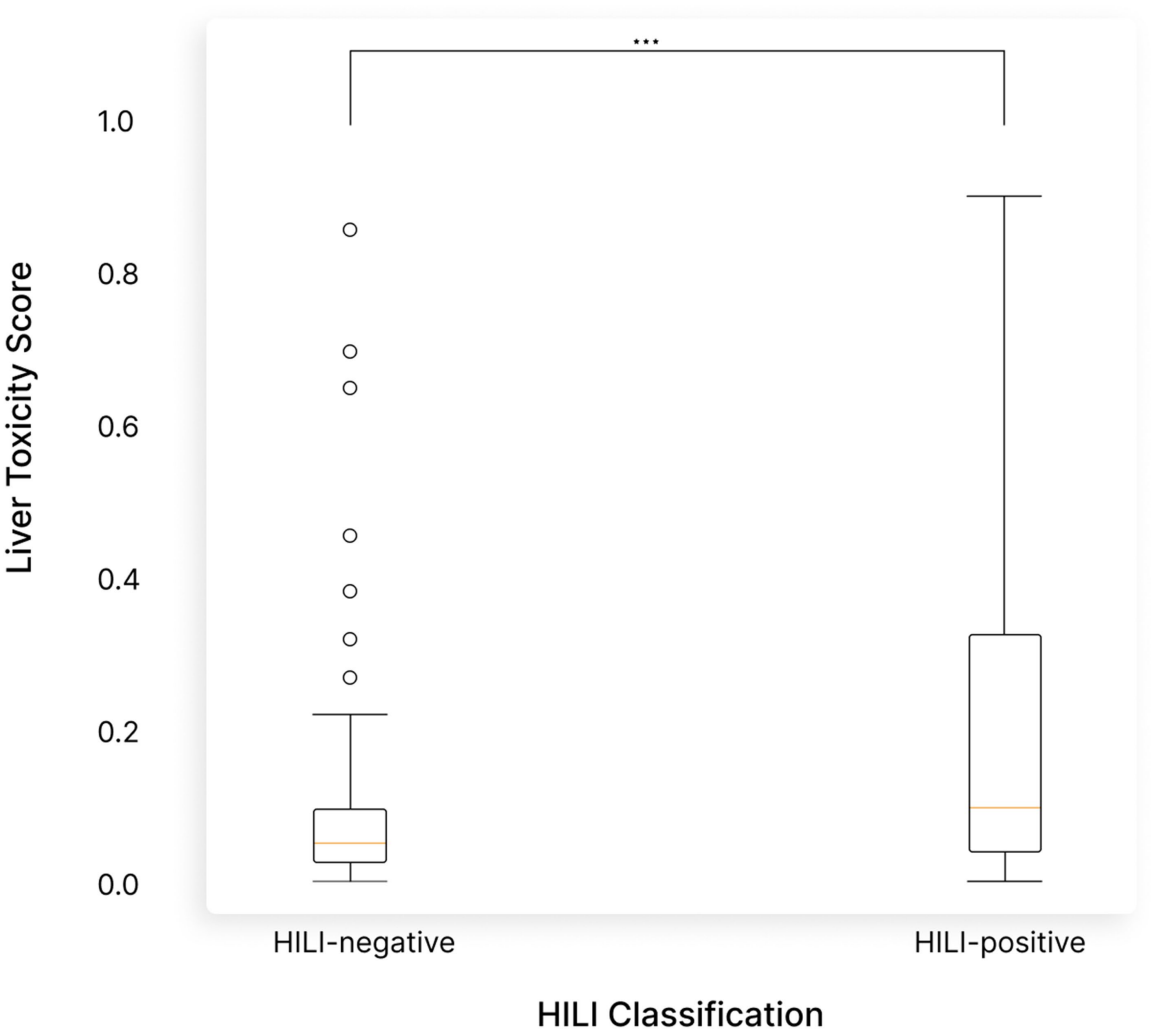
A comparison of liver toxicity current model scores with the classifications made in the MDPI article (42). Herbs classified as HILI-positive (Herbal-Induced Liver Injury) in the article had significantly higher liver toxicity scores in our analysis (*p*-value = 8.38e-05), validating the accuracy of our model. In contrast, Ashwagandha’s liver toxicity score is aligned with herbs classified as HILI-negative, supporting the conclusion that Ashwagandha poses minimal risk for liver toxicity.

We compared our liver toxicity scores to the classifications made in the MDPI article, specifically contrasting herbs they classified as HILI-positive versus HILI-negative. The herbs classified as HILI-positive had significantly higher liver toxicity scores in our analysis (*p*-value = 8.38e-05), further validating our approach (Figure 6). In contrast, Ashwagandha’s liver toxicity score was consistent with herbs classified as HILI-negative, supporting the conclusion that Ashwagandha is not a significant liver toxicity risk (Figure 5).

The results of our NLP analysis indicate that Ashwagandha’s toxicity profile is comparable to other edible plants and herbal supplements, with no statistically significant differences across various toxicity parameters. Ashwagandha consistently ranked below average for general toxicity, liver toxicity, endocrine disruption, thyroid toxicity, and fetal toxicity. While its cytotoxicity was slightly higher, this aligns with its known anti-cancer properties and does not raise concerns for general safety. Furthermore, our comparison with HILI-positive herbs reinforced that Ashwagandha poses minimal risk of liver toxicity, as confirmed by its negative classification for HILI in the literature.

### Structural toxicity predictions demonstrate a low toxicity profile for the root of Ashwagandha

The aim of this section was to evaluate the liver and reproductive toxicity of the molecules present in Ashwagandha, with a specific focus on its root. To achieve this, we predicted the toxicity profiles of the molecules and classified them based on their likelihood of being found in the root versus other parts of the plant. The safety profile of the root was then compared to the rest of the plant, as well as to a broader dataset of edible plants and herbal supplements.

#### Ashwagandha root is predicted to be rich in withanolides and withanolides derivatives

As outlined in the methods, we predicted the presence of molecules in different parts of Ashwagandha using their SMILES representations. Of the 338 molecules identified in Ashwagandha, our model predicted that 79 are likely to be found in the root with a confidence threshold of 0.7 (Supplementary Table 5).

**Figure 7:**
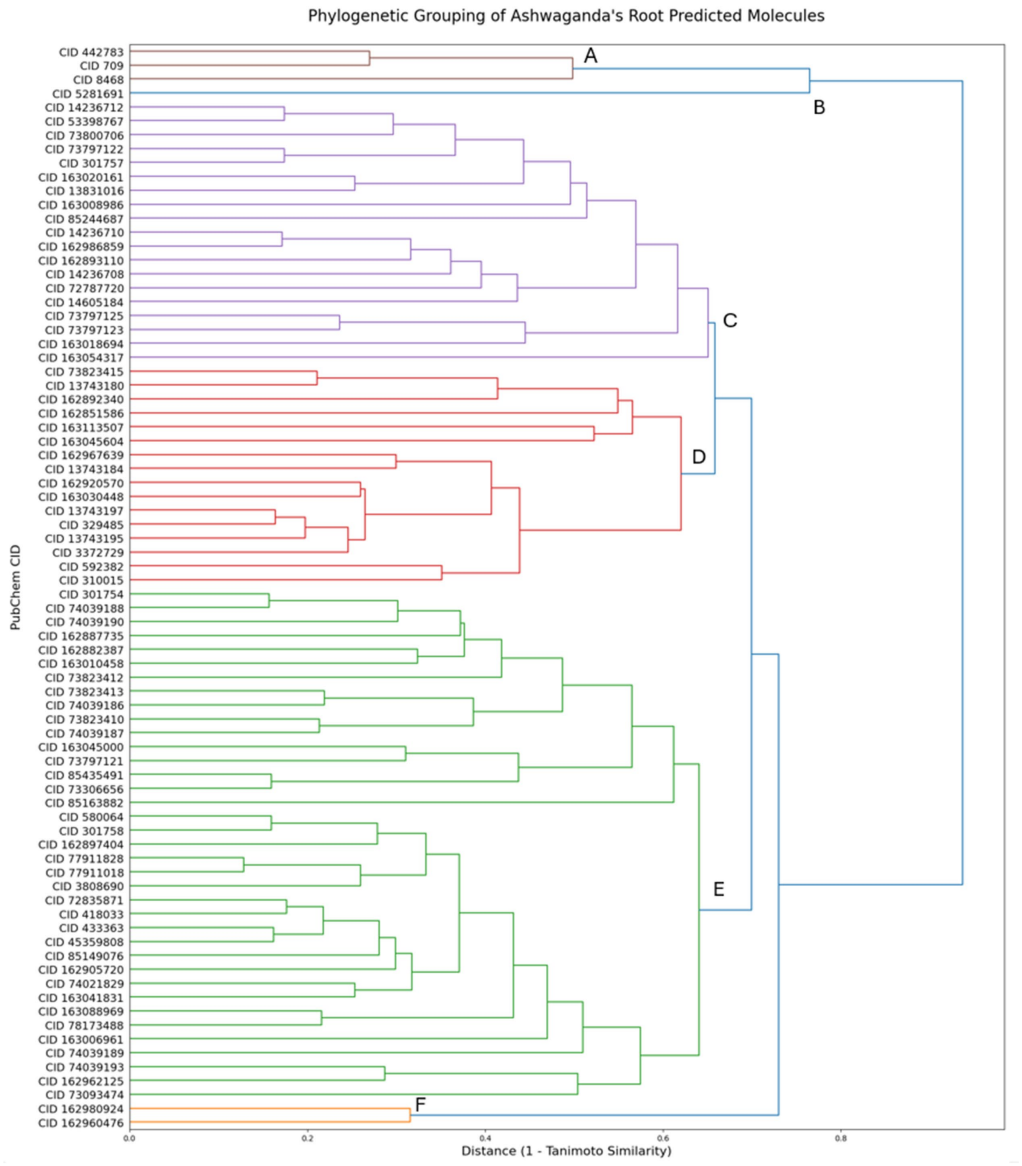
Phylogenetic tree clustering the 79 molecules predicted in the root of *Withania somnifera* (Ashwagandha) into six groups. The Tanimoto similarity index and Morgan fingerprint were used to compute the structural similarity between the molecules, resulting in the identification of six molecular clusters (A-F, see also Table 1). These clusters highlight the structural relationships between molecules, with the largest clusters corresponding to withanolides and withanolide derivatives, alongside smaller clusters of steroid lactones, flavonols, and curcuminoids. Molecules are displayed by PubChem CID.

To further explore the relationships between these molecules, we used the Tanimoto similarity index and Morgan fingerprints to construct a phylogenetic tree, which allowed us to cluster the molecules into six distinct groups based on their structural similarities. By Tanimoto similarity, the distance between clusters represents the average distance between the molecules on the clusters. Each of these groups, marked as A-F in Figure 7, was then classified according to their molecular class using the Coconut database, as shown in Table 1. Clusters D and F correspond to withanolides and their derivatives. Withanolides are a group of naturally occurring steroidal lactones predominantly found in the *Withania* genus, in particular in *Withania somnifera*. They are well known for their diverse bioactivities, including anti-inflammatory, anticancer, and neuroprotective properties.

**Table 1:** Description of the molecules found in each cluster based on the approach outlined in Figure 7.

Cluster	PubChem CID	Chemical class
Brown (A)	CID 8468, CID 445858, CID 442783	M-methoxybenzoic acids and derivatives, Hydroxycinnamic acids, Curcuminoids
Blue (B)	CID 5281691	Flavonols
Purple (C)	CID 592382, CID 310015, CID 3372729, CID 13743195, CID 329485, CID 13743197, CID 162920570, CID 163030448, CID 162967639, CID 13743184, CID 163045604, CID 163113507, CID 162851586, CID 162892340, CID 73823415, CID 13743180	Steroid lactones
Red (D)	CID 592382, CID 310015, CID 3372729, CID 13743195, CID 329485, CID 13743197, CID 162920570, CID 163030448, CID 162967639, CID 13743184, CID 163045604, CID 163113507, CID 162851586, CID 162892340, CID 73823415, CID 13743180	Steroid lactones, Withanolides and derivatives
Green (E)	CID 73093474, CID 162962125, CID 74039193, CID 74039189, CID 73190083, CID 163006961, CID 78173488, CID 163088969, CID 163041831, CID 74021829, CID 162905720, CID 85149076, CID 433363, CID 45359808, CID 72835871, CID 418033, CID 3808690, CID 77911018, CID 77911828, CID 162897404, CID 301758, CID 580064, CID 85163882, CID 85435491, CID 73306656, CID 73797121, CID 163045000, CID 74039187, CID 73823410, CID 73823413, CID 74039186, CID 73823412, CID 163010458, CID 162882387, CID 162887735, CID 74039190, CID 74039188, CID 301754	Withanolides, Withanolides glycosides and derivatives
Orange (F)	CID 162980924, CID 162960476	Withanolides and derivatives

Tanimoto similarity index and Morgan fingerprints were used to group molecules predicted to be present in the root of *Withania somnifera* (Ashwagandha). The table includes the PubChem CID for each molecule, and their corresponding molecular groups (see also Supplementary Table 5).

We found that the largest clusters corresponded to withanolides and their derivatives, which are well-known for their bioactive properties, particularly in stress response and anti-cancer activities. Additionally, we identified steroid lactones, flavonols, and curcuminoids in smaller clusters, all of which contribute to the diverse bioactivity associated with Ashwagandha’s therapeutic uses. These classifications help to provide a clearer understanding of the molecular diversity in Ashwagandha’s root, highlighting its potential as a source of various bioactive compounds with different structural properties.

#### None of Ashwagandha liver toxic molecules are predicted to be in the root

Using the approach outlined earlier, we scanned all 338 identified molecules from Ashwagandha to assess their potential for liver toxicity. Of these, only two molecules, L-Ala (PubChem CID 602) and Tropine (PubChem CID 8424), were predicted to exhibit liver toxicity, and even these predictions were made with medium confidence (0.7 and 0.72 respectively). L-Ala (L-Alanine) is a naturally occurring amino acid, typically found in proteins and known to play a role in energy metabolism. While generally considered safe, some studies suggest that in excess or under certain metabolic conditions, it could contribute to liver strain (35). Tropine, a tropane alkaloid, is found in several plant species and is known for its anticholinergic properties. Some alkaloids of this class have been associated with toxicity, particularly when consumed in large quantities, potentially leading to adverse effects on the liver (36). Notably, neither of these molecules was predicted to be present in the root, based on the threshold we selected (0.7 and above) (Supplementary Table 5).

**Figure 8:**
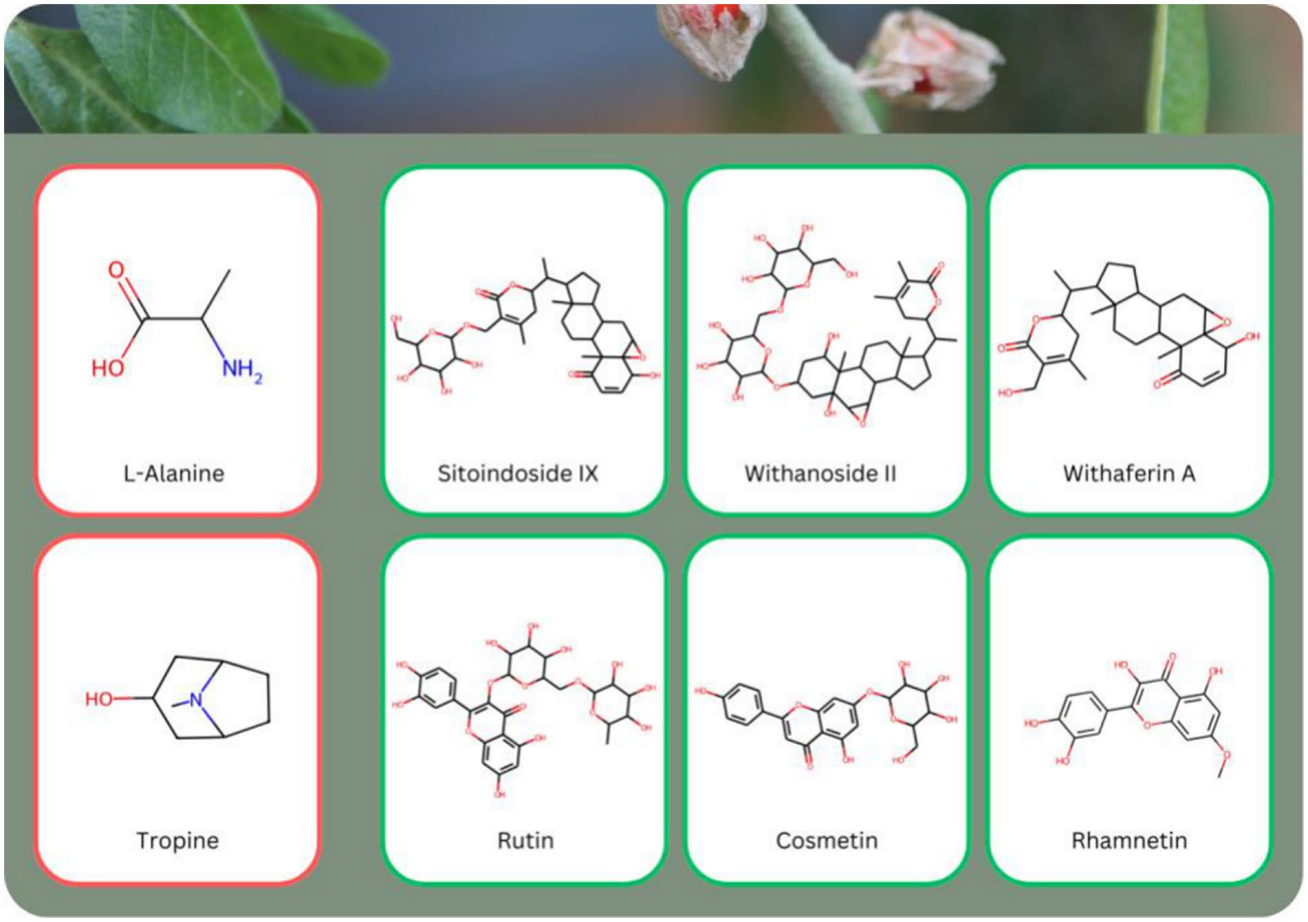
Examples of eight molecules known to be present in *Withania somnifera* (Ashwagandha) with varying predicted liver toxicity levels. Two molecules, L-Ala and Tropin, were predicted to exhibit some level of liver toxicity (red frame), while the remaining six molecules, including Withanolides such as Sitoindoside IX, Withanoside II, Withaferin A, and Flavonoids such as Rutin, Cosmetin, and Rhamnetin, were predicted to be safe for the liver (green frame). None of the toxic molecules were predicted to be present in the root of Ashwagandha (see also Supplementary Table 5).

In contrast, 143 molecules from Ashwagandha were predicted to be non-toxic with very high confidence, as their liver toxicity confidence was below 0.2. This group included several key bioactive compounds such as Withanolides, including: *Sitoindoside IX* (PubChem CID 78173488), *Withanoside II* (PubChem CID 163025782), *Withaferin A* (PubChem CID 580064), and flavonoids, including: *Rutin* (PubChem CID 5293655), *Cosmetin* (PubChem CID 5385553), *Rhamnetin* (PubChem CID 5281691) that are depicted in Figure 8.

**Figure 9:**
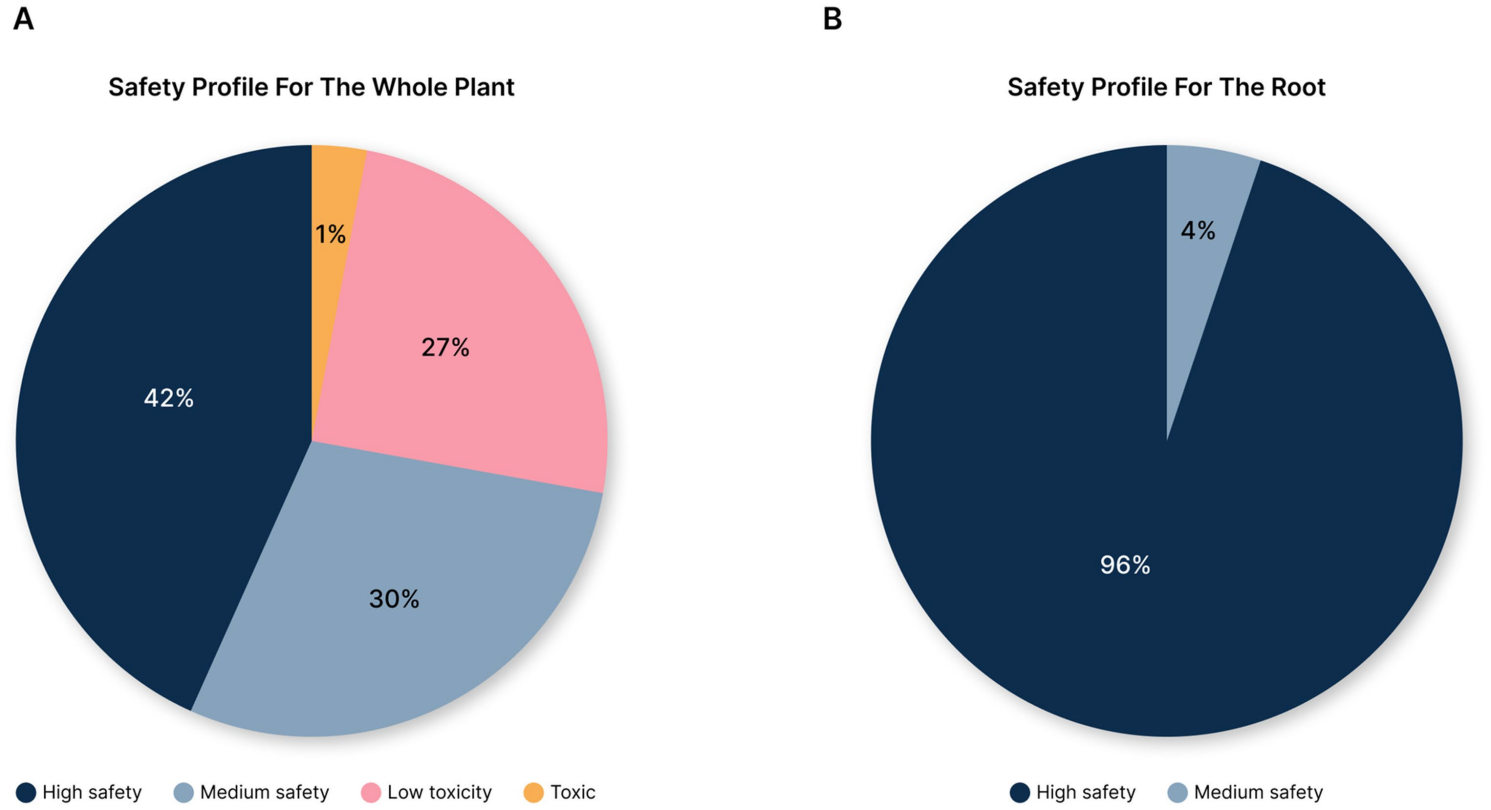
Liver toxicity safety profile of *Withania somnifera* (Ashwagandha): comparative analysis of whole plant and root. **(A)** Pie chart illustrating the liver toxicity safety profile for the whole plant of Ashwagandha, showing the proportion of molecules in each toxicity category. 42% of the molecules were classified as having high safety (liver toxicity confidence < 0.2), while 30% were in the medium safety category (liver toxicity confidence < 0.5). 27% of the molecules were predicted to have low liver toxicity (liver toxicity confidence between 0.5 and 0.7), and only two molecules were predicted to be liver toxic (liver toxicity confidence above 0.7). **(B)** Pie chart illustrating the liver toxicity safety profile for the root of Ashwagandha. No molecules were predicted to have liver toxicity. 96% of the molecules fell into the high safety category (liver toxicity confidence < 0.2), confirming the root’s extremely safe profile in terms of liver toxicity.

These findings indicate that the safety profiles of the molecules differ significantly between the root and the whole plant (Figures 9A,B). As mentioned, for the whole plant, only two molecules (L-Ala and Tropin) are predicted to be liver toxic. An additional 90 molecules (27% of the total molecules) are predicted to exhibit low liver toxicity, with confidence levels between 0.5 and 0.7. By contrast, for the root, all of the molecules are predicted to be safe for the liver, with toxicity confidence levels below 0.5. Even more striking, 96% of the molecules in the root were predicted to be extra safe, with liver toxicity confidence scores below 0.2. Overall, very few molecules in Ashwagandha are predicted to pose a liver toxicity risk, and none of these are predicted to be present in the root. These findings suggest that the root of Ashwagandha is particularly safe for liver health, further supporting its traditional use in herbal medicine.

**Figure 10:**
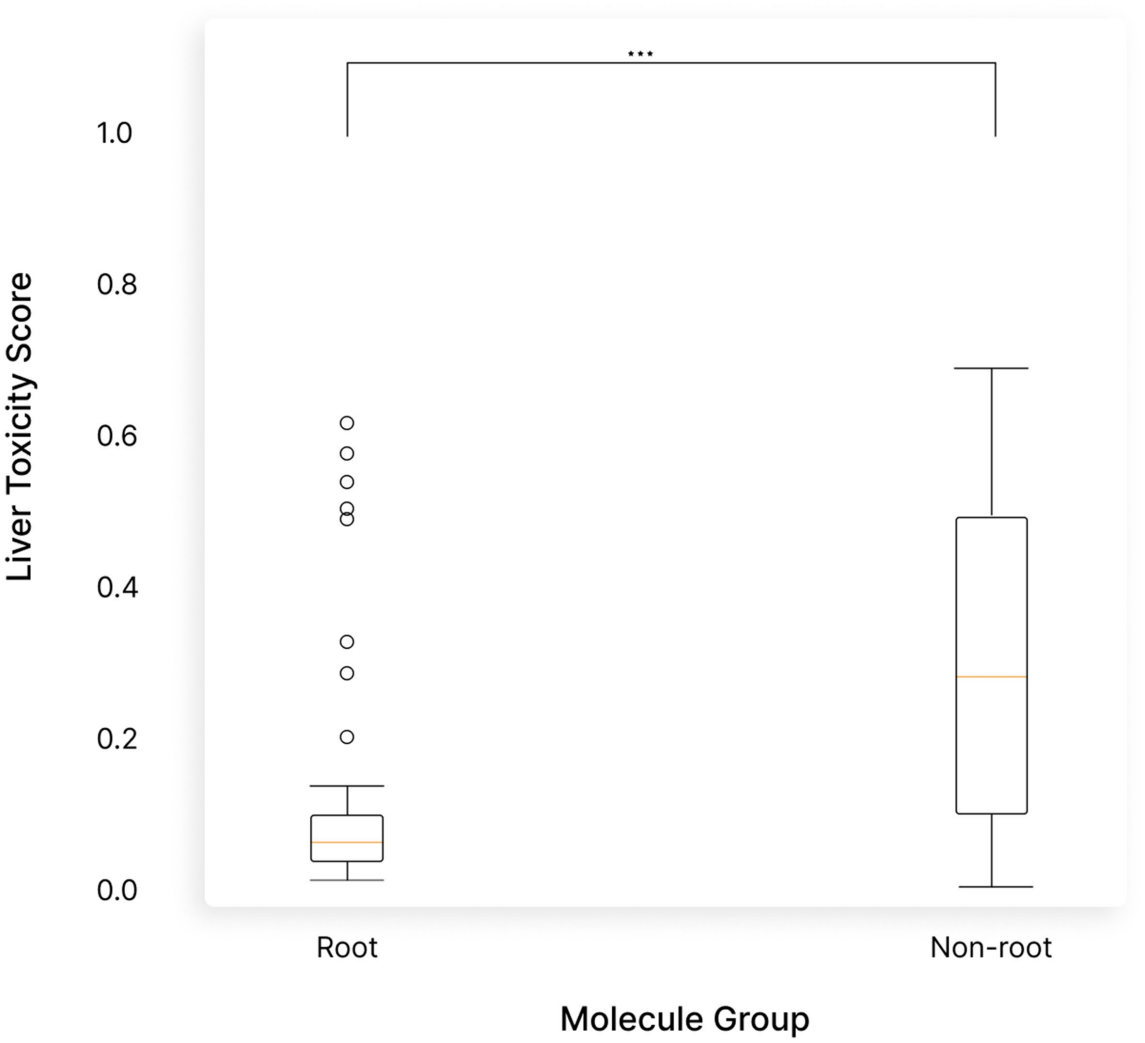
Molecules from the root of *Withania somnifera* (Ashwagandha) were significantly safer for the liver compared to those predicted to be in non-root parts. Boxplots comparing liver toxicity predictions showed a strong statistical difference, with root molecules having a significantly lower toxicity (*p*-value = 2.24e-12). This indicates that root-derived molecules are much safer for the liver.

Next, we analyzed whether the observed differences between the predicted liver toxicity of the root and non root molecules is statistically significant. For this, a non-parametric Wilcoxon test was performed. Root molecules appeared to be significantly safer for the liver than their non root counterparts with a *p*-value of 2.24e-12 (Figure 10).

#### A comparative analysis with edible plants and herbal supplements further strengthened the liver safety of Ashwagandha root

To further validate Ashwagandha’s safety, we compared its molecular safety profile to a set of 676 edible plants and 227 herbal supplements. For this analysis, we computed a toxicity score that accounted for the proportion of molecules with predicted liver toxicity (liver toxicity confidence above 0.5) and weighted this based on the confidence of the predictions.

**Figure 11:**
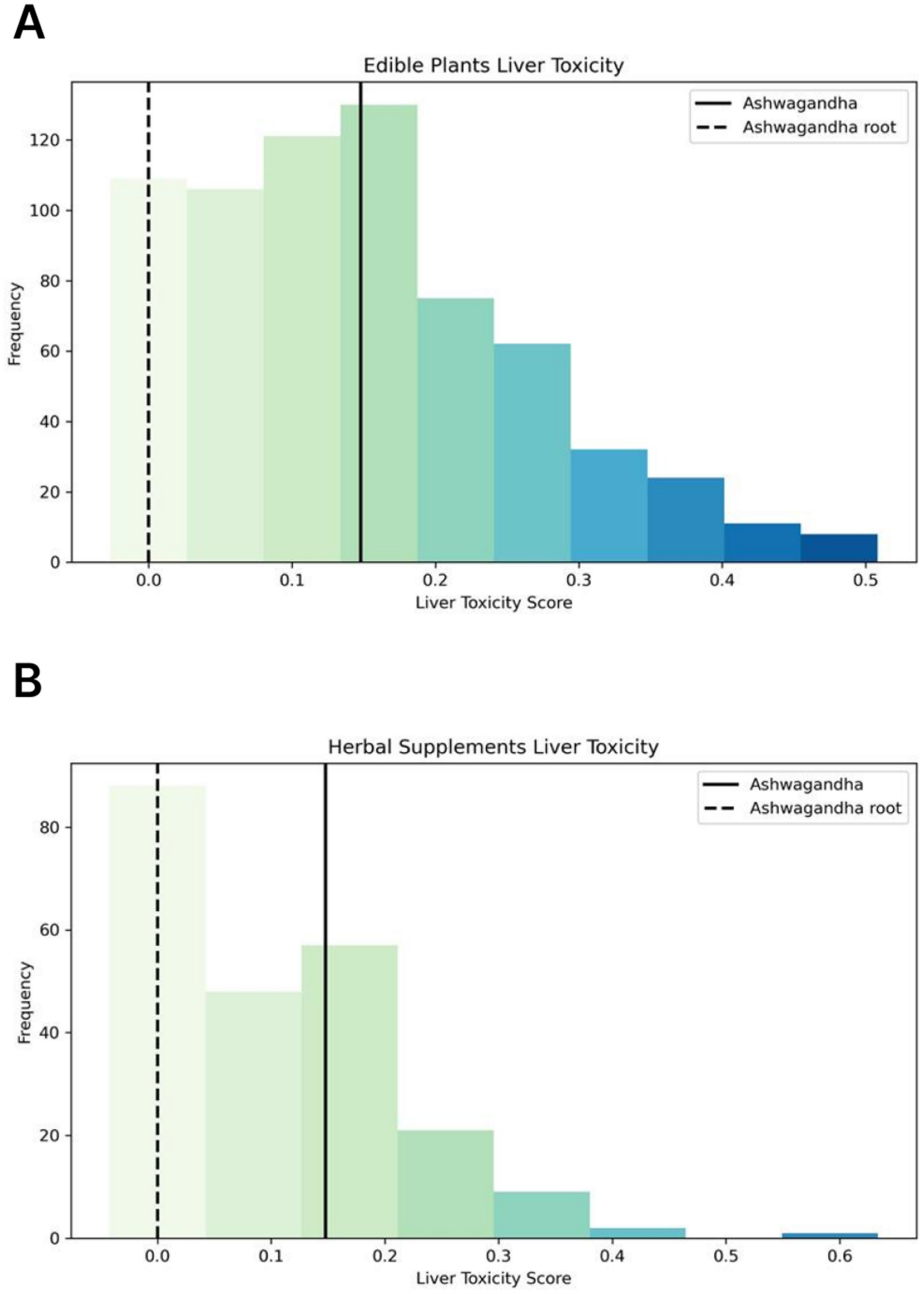
Comparative analysis of liver toxicity scores. **(A)** Histogram shows the distribution of liver toxicity scores based on the molecular composition of edible plants. The liver toxicity scores for Ashwagandha are represented by a straight line for the whole plant and a dashed line for the root only, highlighting the superior safety profile of Ashwagandha root in comparison to other edible plants. **(B)** Histogram showing the distribution of liver toxicity scores based on the molecular composition of 227 herbal supplements. The liver toxicity scores for Ashwagandha are represented by a straight line for the whole plant and a dashed line for the root only, emphasizing the superior safety profile of Ashwagandha root compared to other herbal supplements.

When compared to the broader set of edible plants and supplements, Ashwagandha emerged as one of the plants with a strong safety profile, performing better than the average in our dataset. This was true when considering all molecules from Ashwagandha, but the root, in particular, displayed an even stronger safety profile (Figures 11A,B). These findings align with the results obtained from our previous NLP analysis, further reinforcing Ashwagandha’s reputation as a safe herbal supplement.

Our approach was validated through the successful identification of known toxic molecules in certain herbs, with our model’s predictions supported by literature evidence. For instance, in Comfrey, one of the molecules flagged by our model as toxic was a protoberberine alkaloid (PubChem CID 440229) that was also highlighted in the literature (37). The toxicological effects of Comfrey have been well-documented, particularly in studies on genotoxicity and carcinogenicity. Our model identified three toxic molecules in Ephedra, including two benzenes and synephrine. Synephrine, in particular, has been linked to toxic effects, especially when used in combination with other substances such as ephedrine and caffeine (38). This finding is in line with concerns about the hepatotoxicity of weight-loss products containing Ephedra. Known for its hepatotoxic potential, Kava was another herb where our model successfully identified toxic molecules. Interestingly, the same toxic molecule found in Comfrey was also present in Kava. According to Olsen et al., the toxicity in Kava is also believed to be related to alkaloids (39).

This comparative analysis underscores Ashwagandha’s favorable safety profile, particularly when focusing on the root, and supports the effectiveness of our model in identifying toxic molecules in other herbs. By successfully predicting known toxic components in plants such as Comfrey, Ephedra, and Kava, our approach demonstrates its reliability and reinforces Ashwagandha’s standing as a safe herbal supplement.

#### Ashwagandha root molecules are predicted to have a high reproductive safety

In addition to assessing liver toxicity, we carried out a similar analysis to evaluate the reproductive toxicity of molecules in Ashwagandha. By reproductive toxicity, we refer to potential activity in hormonal disruption, thyroid toxicity, and fetal toxicity. Our analysis revealed a significant difference in the safety profiles between the root and the aerial parts of the plant (Supplementary Table 5).

Regarding the safety of the root, none of the 79 molecules predicted to be present in the root were identified as having reproductive toxicity. In fact, nearly all (78 out of 79) had a toxicity score below 0.2, meaning their safety confidence was above 0.8, indicating that the root of Ashwagandha is extremely safe in terms of reproductive toxicity.

**Figure 12:**
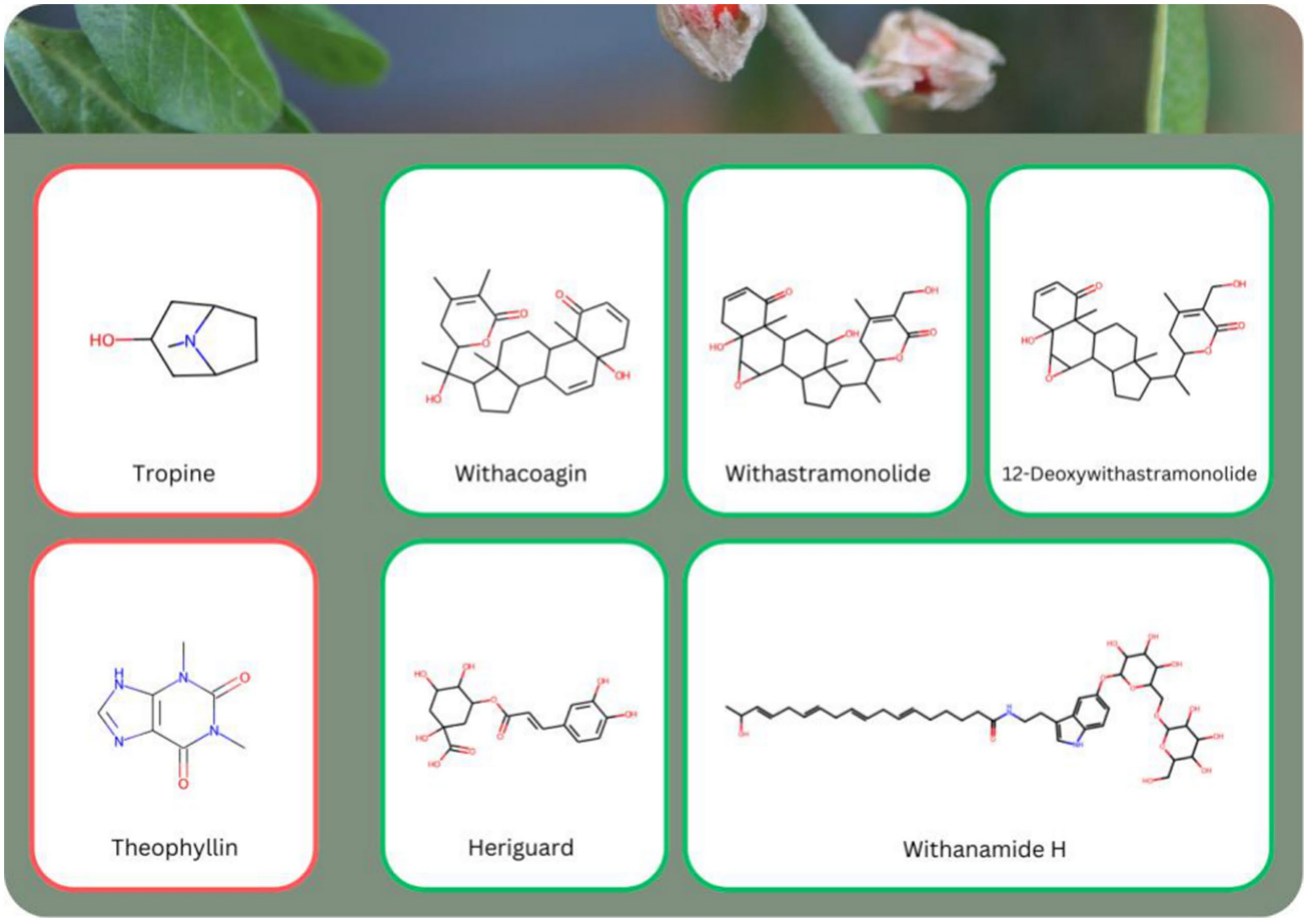
Examples of seven molecules with varying predicted reproductive toxicity levels. Tropine and Theophyllin (red frame) were predicted to show some reproductive toxicity, while the following molecules were predicted to be safe for reproductive health (green frame): Withacoagin, Withastramonolide, 12-Deoxywithastramonolide, Heriguard and Withanamide H. The reproductive toxic molecules are not predicted to be present in the root of Ashwagandha (see also Supplementary Table 5).

In contrast, two molecules in the aerial parts of Ashwagandha were predicted to exhibit reproductive toxicity (Figure 12)—Tropin (PubChem CID 8424), with a toxicity score of 0.91, is a tropane alkaloid known for its anticholinergic properties and potential toxicity. Tropane alkaloids have been linked to toxicity concerns, especially when consumed in higher doses, with effects ranging from hormonal disruption to nervous system impacts (40). Theophyllin (PubChem CID 2153), with a toxicity score of 0.85, is a xanthine derivative commonly found in tea leaves. While theophyllin is used therapeutically to treat respiratory diseases, it has been associated with side effects, including potential reproductive toxicity at high concentrations (41).

Additionally, 47 molecules in the aerial parts were identified with a low toxicity potential (toxicity confidence between 0.5 and 0.7). Despite this, the aerial parts also contained 152 molecules that were non-toxic with high confidence, including notable bioactives such as the Withanolides Withacoagin (PubChem CID 14236708), Withastramonolide (PubChem CID 73800706), and 12-Deoxywithastramonolide (PubChem CID 53398767); and the Organooxygen Compounds Heriguard (PubChem CID 348159) and Withanamide H (PubChem CID 72951313) depicted in Figure 12 and Supplementary Table 5.

**Figure 13:**
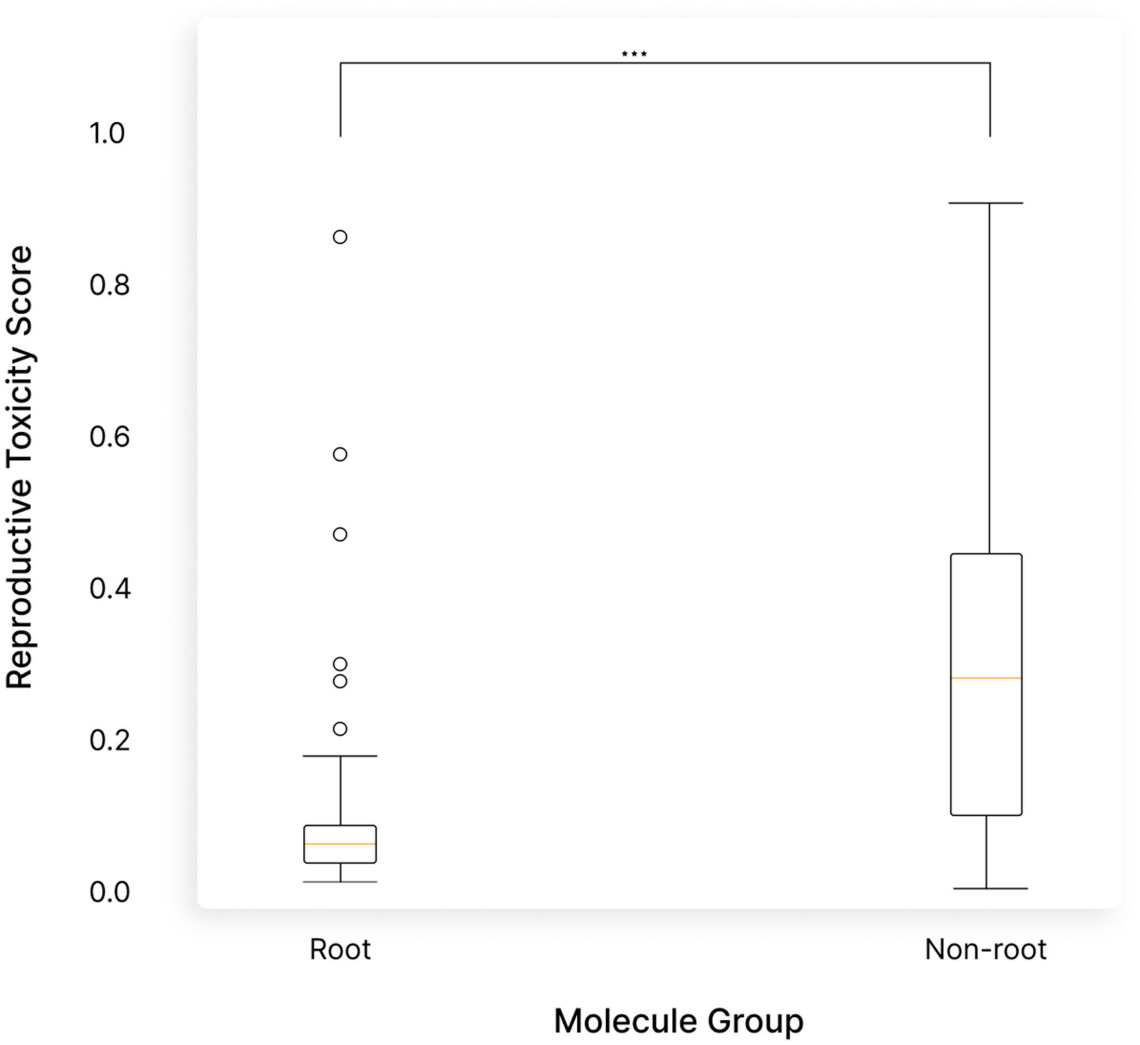
*Withania somnifera* (Ashwagandha) root molecules are significantly safer than those in the non-root parts. A Boxplot comparing the predicted reproductive toxicity of molecules in the root versus the non-root parts of the plant confirmed by Wilcoxon test (*p*-value = 2.31e-15).

To quantify the differences in safety profiles between the root and aerial parts, we conducted a Wilcoxon test. The test confirmed that the differences in toxicity predictions between the root and aerial molecules were statistically significant (*p*-value = 2.31e-15), further underscoring the safety of the root compared to the rest of the plant (Figure 13).

#### When conductive a massive comparative analysis, Ashwagandha root appears to be one of the safest option for reproductive health

To provide further context for Ashwagandha’s reproductive toxicity profile, we compared its safety to our extended dataset of edible plants and herbal supplements. This analysis offered valuable insights into how Ashwagandha ranks among commonly consumed botanicals in terms of reproductive safety, considering hormonal disruption, thyroid toxicity, and fetal toxicity.

**Figure 14:**
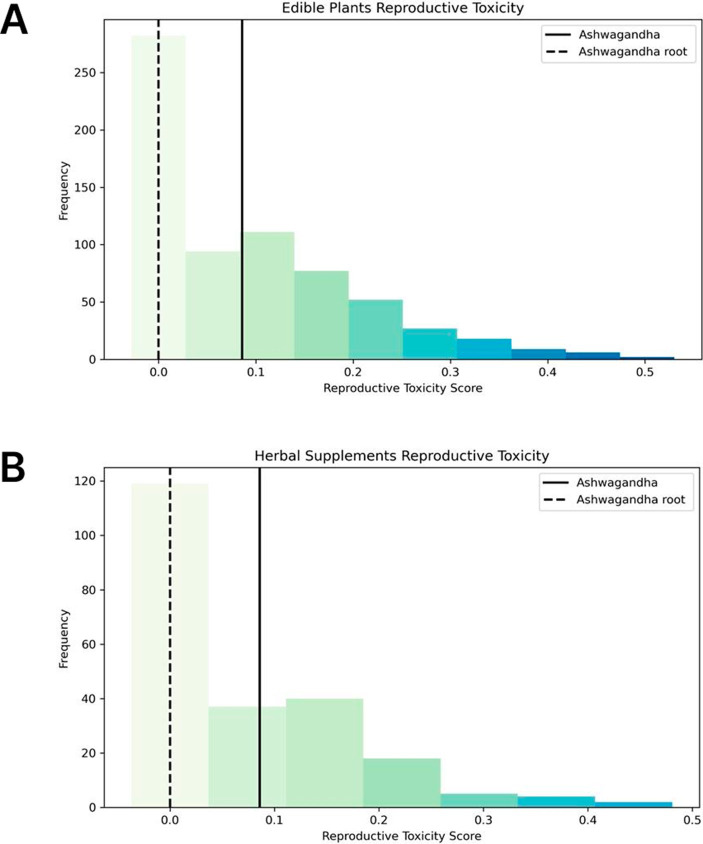
Comparative analysis of reproductive toxic scores. **(A)** Histogram showing the distribution of reproductive toxicity scores based on the molecular composition of 676 edible plants. The reproductive toxicity scores for Ashwagandha are represented by a straight line for the whole plant and a dashed line for the root only, highlighting the superior safety profile of Ashwagandha root in comparison to other edible plants. **(B)** Histogram showing the distribution of reproductive toxicity scores based on the molecular composition of 227 herbal supplements. The reproductive toxicity scores for Ashwagandha are represented by a straight line for the whole plant and a dashed line for the root only, highlighting the superior safety profile of Ashwagandha root in comparison to other herbal supplements.

When evaluating the whole plant, Ashwagandha’s reproductive safety profile was found to be comparable to the average safety profiles of both edible plants and herbal supplements. This means that while the whole plant does not stand out as particularly concerning in terms of reproductive toxicity, it falls within the expected range of safety found across a variety of other plants and supplements used in traditional and modern herbal medicine (Figures 14A,B).

However, when focusing specifically on the root of Ashwagandha, the results were markedly different. The root was found to be among the safest in the entire dataset, outperforming the vast majority of the edible plants and herbal supplements in terms of reproductive safety. This is particularly significant as the root contains no molecules predicted to pose reproductive toxicity risks, reinforcing its historical and traditional use in Ayurvedic medicine for its safety and efficacy (Figures 14A,B).

The safety score for Ashwagandha’s root reflected high confidence in the absence of reproductive toxicity, positioning it as an exceptionally safe option among herbal remedies. This stark difference between the safety profile of the root and that of the whole plant further underscores the importance of focusing on the specific parts of plants when assessing safety and toxicity, as the root’s lack of toxic molecules differentiates it from the aerial parts, which did contain some molecules with potential reproductive toxicity.

Our findings echo the results of earlier analyses and further validate Ashwagandha’s long-standing reputation for being a safe and reliable herbal supplement, particularly when derived from the root. In conclusion, Ashwagandha’s root exhibits a superior reproductive safety profile, making it one of the safest options available among both edible plants and widely used herbal supplements.

## Discussion

### Ashwagandha root demonstrates a high safety profile

This study provides a comprehensive evaluation of the safety profile of *Withania somnifera* (Ashwagandha) using advanced NLP-driven meta-analysis alongside QSAR-based molecular predictions. By integrating these methodologies, we assessed the liver and reproductive toxicity of Ashwagandha, focusing primarily on the root. The findings reaffirmed the longstanding perception of Ashwagandha root as a safe ingredient for supplementation.

Liver toxicity predictions indicated minimal risk, with none of the root molecules showing high likelihood of hepatotoxicity. This finding aligns with the plant’s traditional use and the widespread use and the widespread clinical perception of its safety (42). Rare reports of liver toxicity have been described, but the underlying mechanisms remain largely hypothetical. Proposed explanations include idiosyncratic immune-mediated reactions, the formation of reactive metabolites in sensitive individuals, or interactions with concomitant medications leading to altered hepatic metabolism. Importantly, these cases are extremely uncommon relative to the widespread use of Ashwagandha, and no consistent pattern of hepatotoxicity has been established. Moreover, most reports describe reversible events that resolve upon discontinuation, suggesting a non-intrinsic, individual-specific susceptibility rather than a general toxic effect of withanolides. These rare case reports do not contradict the high safety profile that was obtained according to our computational predictions.

Reproductive toxicity analysis also confirmed that Ashwagandha’s root posed no significant risk in terms of hormonal disruption, thyroid toxicity, or fetal toxicity. Notably, none of the molecules found in the root were flagged for reproductive toxicity. This is in accordance with previous studies that have highlighted benefits of the root extract for the hormonal system, as well as experiments on animal models that have demonstrated its fetal safety (43–45).

A comparative analysis with edible plants and herbal supplements we conducted reinforced Ashwagandha’s exceptional safety profile. The root, in particular, outperformed many other botanicals, highlighting its suitability for inclusion in a wide range of health products. Altogether, these results emphasize Ashwagandha’s strong safety profile, validating both its traditional use in Ayurvedic medicine and its increasing presence in modern supplements.

### The root of Ashwagandha poses significantly less concerns than its aerial counterparts

Our study found significant differences between the root and aerial parts of the plant in terms of predicted safety. The root, in particular, demonstrated an exceptionally safe profile. None of the 79 molecules found in the root were predicted to cause liver toxicity. For example, molecules like Withanolide A (with a liver toxicity confidence of 0.05) and 12-Deoxywithastramonolide (with a liver toxicity confidence of 0.042) showed very low toxicity, reinforcing the safety of the root for liver health. In fact, 96% of the molecules in the root were predicted to have extremely low toxicity (with a confidence score below 0.2), further highlighting its safety.

In contrast, the non-root parts of the plant contained two molecules—L-Ala and Tropin—that were predicted to have medium-level liver toxicity, with confidence scores above 0.7. Additionally, 90 molecules (about 27% of the non-root molecules) showed a potential for low to moderate liver toxicity, with confidence levels ranging from 0.5 to 0.7.

In terms of reproductive toxicity, the root exhibited a similarly safe profile in terms of reproductive toxicity, with none of the 79 root molecules predicted to have any toxic effects. Almost all root molecules (78 out of 79) had toxicity scores under 0.2, indicating high safety with respect to hormonal disruption, thyroid toxicity, and fetal toxicity. Key molecules such as Withanolides (*Withacoagin* and *Withanoside V*) and flavonoids (*Rutin* and *Withanamide H*) demonstrated very low reproductive toxicity potential.

On the other hand, the non-root parts posed a higher risk, with Tropin (reproductive toxicity confidence: 0.91) and Theophyllin (reproductive toxicity confidence: 0.85) being flagged for potential reproductive toxicity. This is in line with the literature that highlighted fetal toxicity for theophyllin (46). Additionally, 47 molecules from the non-root parts had low to moderate toxicity predictions (confidence between 0.5 and 0.7).

These differences between the root and non-root parts of Ashwagandha highlight the importance of focusing on the root for medicinal and dietary applications. The root consistently showed a superior safety profile in both liver and reproductive toxicity compared to the rest of the plant. This distinction aligns with traditional uses of Ashwagandha, which emphasize the root as the primary source of its therapeutic benefits.

### Withanolides have a high safety profile, making them a great option for safe and effective therapeutics

As shown by the phylogenetic analysis (Figure 7.), Ashwagandha’s root is rich in withanolides and their derivatives. Withanolides and their derivatives have been extensively investigated in the literature, with a broad range of reported therapeutic effects. These include anti-inflammatory, anticancer, immunomodulatory, neuroprotective, and adaptogenic activities, making them one of the most well-characterized classes of natural products in terms of pharmacological potential. For example, withaferin A, known for its potent cytotoxic activity against cancer cells Devi et al., Xing et al. and Misra et al., was predicted to have high liver and reproductive safety according to our analysis (18, 20, 47). This is particularly important given its reported efficacy in anti-cancer treatments, making it a promising option that combines therapeutic benefits with a favorable safety profile. Other withanolides, which have also been reported for their anti-cancer properties, were similarly predicted with high confidence to be present in Ashwagandha root and exhibited a high safety profile for both liver and reproductive toxicity (48, 49). These findings further emphasize the potential of Ashwagandha root in safe and effective therapeutic applications, particularly in oncology.

### A groundbreaking AI approach for safety evaluation

Our AI-driven methodology represents a significant leap forward in the field of safety evaluation for herbal supplements and natural products. By combining cutting-edge Natural Language Processing (NLP) models with advanced Quantitative Structure–Activity Relationship (QSAR) predictions, we have developed a powerful and highly accurate system for assessing the toxicity of plant molecules. This approach not only surpasses traditional methods but also sets a new standard for safety evaluation.

Unlike conventional safety assessments, which often rely on labor-intensive and time-consuming laboratory testing, our AI platform offers a rapid and scalable solution. It can process vast amounts of data from scientific literature, as well as predict the toxicity of thousands of molecules based on their structure, allowing for the assessment of plants and supplements that may not have been thoroughly studied. Our system leverages a proprietary database containing molecular data from over 60,000 organisms, which provides a robust foundation for generating accurate and reliable predictions.

The key advantage of this AI-based approach lies in its ability to predict both liver and reproductive toxicity at a molecular level. This precision enables us to evaluate specific plant parts, such as the root of Ashwagandha, with unprecedented granularity. The differentiation between safe and potentially harmful plant parts in our study underscores the strength of our methodology and its potential to transform how safety evaluations are conducted.

By accurately predicting toxicity profiles, our AI model can streamline the R&D process for manufacturers, reducing the need for extensive animal testing and costly *in vitro* experiments. This technology offers a future where safety evaluations are faster, more comprehensive, and more predictive, allowing companies to develop safer, more effective products for consumers. As a result, our approach has the potential to become the gold standard in the industry, providing confidence in the safety of herbal supplements and setting a new benchmark for regulatory compliance and product development.

### Ashwagandha root presents an unparalleled opportunity for the development of safe herbal supplements

The traditional use of Ashwagandha, particularly its root, has long been celebrated in Ayurvedic medicine for its adaptogenic properties, promoting resilience against stress, improving cognitive function, and supporting overall vitality. Modern research has confirmed many of these traditional claims, with studies validating Ashwagandha’s anti-inflammatory, neuroprotective, and stress-relieving effects (1, 50, 51). The findings of this study further support these traditional uses by highlighting the superior safety profile of the root, especially in terms of liver and reproductive toxicity. Unlike the non-root parts, which carry a higher toxicity risk, the root was found to be remarkably safe, with none of the predicted molecules showing significant toxicity. This distinction is crucial for the herbal supplement industry, where consumer demand for safe, natural products is growing. By focusing on the root, manufacturers can align with both traditional wisdom and modern scientific validation, offering consumers products that are not only effective but also safe. As regulatory scrutiny on herbal supplements increases, the clear safety advantage of Ashwagandha root provides an opportunity for innovation, allowing companies to confidently develop new products that meet both safety standards and consumer expectations.

Recent studies have suggested the use of Ashwagandha leaves due to their potential biological activities, such as anti-inflammatory and immunomodulatory effects (52, 53). However, while these properties are intriguing, our analysis strongly suggests that it is safer to focus on the root rather than the leaves. The non-root parts, including the leaves, contain molecules like Tropin and Theophyllin, which were predicted to have significant toxicity concerns, particularly in terms of reproductive toxicity (54). In contrast, the root is not only free of these toxic molecules but also rich in withanolides and their derivatives—the most beneficial and safe compounds found in Ashwagandha. These molecules, such as Withanolide A and Withanoside V, are well-known for their adaptogenic, anti-inflammatory, and neuroprotective effects and were predicted with high confidence to be safe for both liver and reproductive health (55, 56). By focusing on the root, manufacturers can ensure a safer product that aligns with both traditional usage and modern scientific evidence, avoiding the potential risks associated with using the leaves.

### Limitations

A potential limitation regarding reproducibility is access to the proprietary MeNow database. Due to commercial constraints, this database is not openly available for independent use. However, this does not represent a major obstacle for reproducibility, as we have taken extensive steps to ensure transparency. Specifically, our methodology, selection criteria, and use of public databases are described in detail, and we provide in both the main tables and the Supplementary material the full list of compounds considered together with the relevant information needed for verification. This enables other researchers to reproduce the key steps of our analysis, even without direct access to the internal database.

## Conclusion

Through a combined AI-driven meta-analysis and QSAR-based molecular assessment, this study provides strong evidence suggesting that *Withania somnifera* (Ashwagandha), particularly its root, exhibits a favorable safety profile compared to other herbal supplements and edible plants. Both literature-based and molecular-level analyses consistently indicated a low potential for liver and reproductive toxicity in the root, while the non-root parts displayed comparatively higher, but still limited, toxicity potential. These findings are consistent with the traditional Ayurvedic preference for root-based formulations and support its continued use as a generally safe component in herbal preparations. Moreover, this research demonstrates the utility of AI methodologies in toxicological evaluation, offering a scalable, evidence-based approach for assessing the safety of natural products. Future clinical and experimental studies will be valuable to confirm and refine these computational insights, further strengthening the foundation for safe and effective use of Ashwagandha in modern health formulations.

## Data Availability

Publicly available datasets were analyzed in this study. This data can be found at: https://coconut.naturalproducts.net/ and http://sideeffects.embl.de/.

## Footnotes

^1^ http://sideeffects.embl.de/

^2^ https://www.menow.ai
