# Supplementary material for: Comprehensive safety evaluation of Withania somnifera (Ashwagandha): an AI-driven meta-analysis and quantitative structure–activity relationship based toxicity assessment
Source: Front Nutr. 2025 Nov 24;12:1658265. doi: 10.3389/fnut.2025.1658265 (PMC12682666; doi:10.3389/fnut.2025.1658265)
Supplement: Supplementary file 1 [file Data_Sheet_1.PDF]

Supplementary table 1 – Prompts and models settings

The following prompt was used to check all NLP models mentioned:

“I have an article of a scientific paper,it's about a plant, can

you tell me if the plant is toxic or not? if it's toxic, only answer me with

'1', if it's not, only answer me with '0'. "

The article is:{abstract}”

Where { abstract } is the article’s abstract tested by prompting

The following prompt was used on GPT 4 (OpenAI) in order to check more toxicities for articles that were found to be toxic:

“I have an abstract of a scientific paper,it's about a plant,

according to the paper, the plant is toxic, I need to know what type of

toxicity it has. I'll ask you to give me an answer to several of them

Answer me in the form of '1' and '0' separated by a comma for each type

I'm asking you for,by the order I'll write them, '1' means positive for that toxicity and '0' means negative

The types I need you to look for are: toxicity for cancer cells (cytotoxicity),

liver toxicity, endocrinien disruption, thyroid toxicity and fetal toxicity

The abstract is:{abstract}"

Where { abstract } is the article’s abstract tested by prompting

Settings for NLP models:

**GPT 3.5 (OpenAI) and GPT 4 (OpenAI) Settings**	
temperature	1.0
top_p	1.0
n	1
presence_penaly	0
frequency_penalty	0
stream	false
max_tokens	Not set

SciBERT Settings	
num_train_epochs	3
learning_rate	2e-5
eval_strategy	epoch
load_best_model_at_end	true

Settings for QSAR models:

The models used was scikit-learn's RandomForestClassifier.

If a parameter is not mentioned, default settings were used. (see scikit-learn's documentation for details)

QSAR model for liver toxicity Settings	
max_depth	8
random_state	0

QSAR model for part of plant Settings	
max_depth	10
random_state	0
n_estimators	100
min_samples_split	5
