# Supplementary material for: Comprehensive safety evaluation of Withania somnifera (Ashwagandha): an AI-driven meta-analysis and quantitative structure–activity relationship based toxicity assessment
Source: Front Nutr. 2025 Nov 24;12:1658265. doi: 10.3389/fnut.2025.1658265 (PMC12682666; doi:10.3389/fnut.2025.1658265)
Supplement: Supplementary file 2 [file Data_Sheet_2.PDF]

Supplementary table 2 – Abstracts discussing toxicological assessment used for toxicity evaluation. Manual toxicity annotation, as well as the results from the three NLP models are presented. 1 indicates an abstract describing toxicity found in the plant in the report, 0 – no toxic indication was found in the report.

Abstract	Manual toxicity annotation	Toxicity according to SciBERT	Toxicity according to GPT 3.5 (OpenAI)	Toxicity according to GPT 4 (OpenAI)
Kava Kava is an herbal supplement used as an alternative to antianxiety drugs. Although some reports suggest an association of Kava Kava with hepatotoxicity , it continues to be used in the United States due to lack of toxicity characterization. In these studies F344/N rats and B6C3F1 mice were administered Kava Kava extract orally by gavage in corn oil for two weeks, thirteen weeks or two years. Results from prechronic studies administered Kava Kava at 0.125 to 2 g/kg body weight revealed dose-related increases in liver weights and incidences of hepatocellular hypertrophy. In the chronic studies, there were dose-related increases in the incidences of hepatocellular hypertrophy in rats and mice administered Kava Kava for up to 1 g/kg body weight. This was accompanied by significant increases in incidences of centrilobular fatty change. There was no treatment- related increase in carcinogenic activity in the livers of male or female rats in the chronic studies. Male mice showed a significant dose-related increase in the incidence of hepatoblastomas. In female mice, there was a significant increase in the combined incidence of hepatocellular adenoma and carcinoma in the low and mid dose groups but not in the high dose group. These findings were accompanied by several nonneoplastic hepatic lesions.	1	0	1	1
Millions of Americans regularly use herbal supplements, but many are unaware of the potential hidden dangers. Numerous supplements have been associated with hepatotoxicity and, indeed dietary/herbal supplements represent an increasingly common source of acute liver injury. We	1	1	1	1

report a case of acute liver failure requiring liver transplantation associated with the use of Garcinia cambogia, a supplement widely promoted for weight loss. When patients resent with acute hepatitis or liver failure from an unknown etiology, a careful history of supplement use should be performed.				
Açai, the fruit of a palm native to the Amazonian basin, is widely distributed in northern South America, where it has considerable economic importance. Whereas individual polyphenolics compounds in açai have been extensively evaluated, studies of the intact fruit and its biological properties are lacking. Therefore, the present study was undertaken to investigate the in vivo genotoxicity of açai and its possible antigenotoxicity on doxorubicin (DXR)-induced DNA damage. The açai pulp doses selected were 3.33, 10.0 and 16.67 g/kg b.w. administered by gavage alone or prior to DXR (16 mg/kg b.w.) administered by intraperitoneal injection. Swiss albino mice were distributed in eight groups for acute treatment with açai pulp (24 h) and eight groups for subacute treatment (daily for 14 consecutive days) before euthanasia. The negative control groups were treated in a similar way. The results of chemical analysis suggested the presence of carotenoids, anthocyanins, phenolic, and flavonoids in açai pulp. The endpoints analyzed were micronucleus induction in bone marrow and peripheral blood cells polychromatic erythrocytes, and DNA damage in peripheral blood, liver and kidney cells assessed using the alkaline (pH >13) comet assay. There were no statistically significant differences (p > 0.05) between the negative control and the groups treated with the three doses of açai pulp alone in all endpoints analyzed, demonstrating the absence of genotoxic effects. The protective effects of açai pulp were observed in both acute and subacute treatments, when administered prior to DXR. In general, subacute treatment provided greater efficiency in protecting against DXR-induced DNA damage in liver and kidney cells. These protective effects can be explained as the	0	1	0	0

result of the phytochemicals present in açai pulp. These results will be applied to the developmental of food with functional characteristics, as well as to explore the characteristics of açai as a health promoter.				
Yaji, otherwise 'suya sauce', used in serving the meat delicacy called 'Suya', is a complex mixture of spices and additives whose active ingredients on individual basis, are known to have side effects if consumed in excess. The growing concern is that the excessive consumption of Yaji signifies an excessive consumption of a combination of these constituents amongst which are ginger, cloves, red pepper and black pepper; which are mixed without a standardized format. This study was therefore designed to correlate the individual with the combined effects of the excessive consumption of these spices on the histology of the liver of adult rabbits. Involved in this study were 12 rabbits that were divided into six groups of two (2) each (A, B, C, D, E and F). Those in groups B, C, D, E and F, constituted the test groups whereas group A served as the control. For 21 days, test group B were fed with a combination of Ginger (3g), Clove (3g), red Pepper (3g) and Black Pepper (3g) per day while test groups C, D, E and F were fed with ginger (3g), Clove (3g), Red pepper (3g) and Black Pepper (3g) per day respectively. The histological observations show that the excessive consumption of these spices can cause necrosis of liver hepatocytes and therefore acute hepatitis.	1	1	1	1
Dried flower bud of Syzygium aromaticum (clove) is rich in eugenol, an antioxidant and antiinflammatory compound that can protect liver against injury. Clove, besides eugenol, also contains other pharmacologically active phytochemicals such as β-sitosterol and ascorbic acid. This study reports the effect of eugenol-rich fraction (ERF) of clove on liver cirrhosis induced by thioacetamide.	0	1	0	0
BACKGROUND: Docosanyl ferulate (DF) is a behaviourally active GABA(A) receptor complex (GABA(A)R) agonist, recently isolated from the standardized methanolic extract of Withania somnifera Dunal (WSE)	0	0	0	0

root. Previous studies have shown that WSE prevents both ethanol- and morphine-dependent acquisition and expression of conditioned place preference (CPP) and stimulation of dopamine release in the nucleus accumbens shell (AcbSh). AIMS: The study aimed at determining (a) whether DF contributes to WSE's ability to affect the acquisition and expression of ethanol- and morphine-elicited CPP and, given that phosphorylation of extracellular signal-regulated kinase (pERK) in the AcbSh is involved in associative learning and motivated behaviours, (b) whether WSE and DF may affect ethanol- and morphine-induced ERKs phosphorylation in the AcbSh. METHODS: In adult male CD1 mice, DF's effects on the acquisition and expression of ethanol- and morphine-elicited CPP were evaluated by a classical place conditioning paradigm, whereas the effects of WSE and DF on ethanol- and morphine-elicited pERK in the AcbSh were evaluated by immunohistochemistry. RESULTS AND CONCLUSIONS: The study shows that DF, differently from WSE, affects only the acquisition but not the expression of ethanol- and morphine-induced CPP. Moreover, the study shows that both WSE and DF can prevent ethanol- and morphine-elicited pERK expression in the AcbSh. Overall, these results highlight subtle but critical differences for the role of GABA(A)Rs in the mechanism by which WSE affects these ethanol- and morphine-dependent behavioural and molecular/cellular responses and support the suggestion of WSE and DF for the control of different components of drug addiction.				
Withania somnifera glycowithanolides (WSG) were investigated for their preventive effect on the animal model of tardive dyskinesia (TD), induced by once daily administration of the neuroleptic, haloperidol (1.5 mg/kg, i.p.), for 28 days. Involuntary orofacial movements (chewing movements, tongue protusion and buccal tremors) were assessed as TD parameters. WSG (100 and 200 mg, p.o.), administered concomitantly with haloperidol for 28 days, inhibited the induction of the neuroleptic	0	0	1	0

TD. Haloperidol-induced TD was also attenuated by the antioxidant, vitamin E (400 and 800 mg/kg, p.o.), but remained unaffected by the GABA-mimetic antiepileptic agent, sodium valproate (200 and 400 mg/kg, p.o.), both agents being administered for 28 days like WSG. The results indicate that the reported antioxidant effect of WSG, rather than its GABA-mimetic action, may be responsible for the prevention of haloperidol-induced TD.				
A post-synaptic neurotoxic phospholipase A(2) (PLA(2)) has been purified from Indian cobra Naja naja venom. It was associated with a peptide in the venom. The association was disrupted using 8 M urea. It is denoted to be a basic protein by its behavior on both ion exchange chromatography and electrophoresis. It is toxic to mice, LD(50) 1.9 mg/kg body weight (ip). It is proved to be post-synaptic PLA(2) by chymographic experiment using frog nerve-muscle preparation. A glycoprotein, (WSG) was isolated from a folk medicinal plant Withania somnifera. The WSG inhibited the phospholipase A(2) activity of NN-XIa-PLA(2,) isolated from the cobra venom, completely at a mole-to-mole ratio of 1:2 (NN-XIa-PLA(2): WSG) but failed to neutralize the toxicity of the molecule. However, it reduced the toxicity as well as prolonged the death time of the experimental mice approximately 10 times when compared to venom alone. The WSG also inhibited several other PLA(2) isoforms from the venom to varying extent. The interaction of the WSG with the PLA(2) is confirmed by fluorescence quenching and gel-permeation chromatography. Chemical modification of the active histidine residue of PLA(2) using p-brophenacyl bromide resulted in the loss of both catalytic activity as well as neurotoxicity of the molecule. These findings suggest that the venom PLA(2) has multiple sites on it; perhaps some of them are overlapping. Application of the plant extract on snakebite wound confirms the medicinal value associated with the plant.	0	0	1	0

BACKGROUND: Withania somnifera (WS), also known as Ashwagandha, is commonly used in Ayurveda and other traditional medicine systems. WS has seen an increase in worldwide usage due to its reputation as an adaptogen. This popularity has elicited increased scientific study of its biological effects, including a potential application for neuropsychiatric and neurodegenerative disorders. OBJECTIVE: This review aims to provide a comprehensive summary of preclinical and clinical studies examining the neuropsychiatric effects of WS, specifically its application in stress, anxiety, depression, and insomnia. METHODS: Reports of human trials and animal studies of WS were collected primarily from the PubMed, Scopus, and Google Scholar databases. RESULTS: WS root and leaf extracts exhibited noteworthy anti-stress and anti-anxiety activity in animal and human studies. WS also improved symptoms of depression and insomnia, though fewer studies investigated these applications. WS may alleviate these conditions predominantly through modulation of the hypothalamic-pituitary-adrenal and sympathetic-adrenal-medullary axes, as well as through GABAergic and serotonergic pathways. While some studies link specific withanolide components to its neuropsychiatric benefits, there is evidence for the presence of additional, as yet unidentified, active compounds in WS. CONCLUSION: While benefits were seen in the reviewed studies, significant variability in the WS extracts examined prevents a consensus on the optimum WS preparation or dosage for treating neuropsychiatric conditions. WS generally appears safe for human use; however, it will be important to investigate potential herb-drug interactions involving WS if used alongside pharmaceutical interventions. Further elucidation of active compounds of WS is also needed.	0	0	0	0
ETHNOPHARMACOLOGICAL RELEVANCE: Ashwagandha-Withania somnifera (L.) Dunal, well known for its multipotent therapeutic properties has been used in Ayurveda for 3000 years. The plant with	0	0	0	0

more than 50 active phytoconstituents is recognised for its anti-cancerous, anti-diabetic, anti-inflammatory, anti-microbial, and neurotherapeutic properties demonstrated in in vitro studies and chemically induced rodent models. Genetically targeted Parkinson's, Alzheimer's and other neurodegenerative disease models have been created in Drosophila and have been used to get mechanistic insight into the in vivo cellular events, and genetic pathways that underlie respective neurodegenerative condition. But hitherto, there aren't enough attempts made to capitalize the genetic potential of these disease models to validate the therapeutic efficacy of different reagents used in traditional medicine, in the context of specific disease-causing genetic mutations. AIM OF THE STUDY: Drugs discovered using in vitro platforms might fail in several instances of clinical trials because of the genetic heterogeneity and variability in the physiological context found among the patients. Drosophila by virtue of its genetically regulated experimental potential forms an ideal in vivo model to validate the candidate reagents discovered in in vitro screens for their efficacy under specific genetic situations. Here we have used genetically induced alpha-synucleinopathy and tauopathy transgenic fly models to study the efficacy of Ashwagandha treatment, assessing cellular and behavioural parameters. METHODS: We have expressed the disease-causing human gene mutations in specific cell types of Drosophila using GAL4/UAS targeted expression system to create disease models. Human alpha-synuclein mutant (A30P) was expressed in dopaminergic neurons using Ddc-GAL4 driver strain to induce dopaminergic neurodegeneration and assayed for motor dysfunction. Human Tau(E14), mutant protein was expressed in photoreceptor neurons using GMR-GAL4 driver to induce photoreceptor degeneration. Microtubular destability and mitotic arrest in the dividing photoreceptor precursor cells were studied using alphaPH3 antibody. Lysosomal dysregulation caused				

necrotic black spots were induced by Tau(E14) with GMR-GAL4 driver, in a white mutant background. These flies mimicking neurodegenerative conditions were supplemented with different concentrations of Ashwagandha aqueous root extract mixed with regular fly food. The treated flies were analysed for cellular and behaviour parameters. RESULTS: Lifespan assay shows that, Ashwagandha-root extract imparts an extended lifespan in male Drosophila flies which are intrinsically less stress resistant. Motor dysfunction caused due to human alpha-synuclein mutant protein expressed in dopaminergic neurons is greatly brought down. Further, Ashwagandha extract treatment significantly reduces Tau(E14) induced microtubular destability, mitotic arrest and neuronal death in photoreceptor neurons. Our experiment with tauopathy model in white mutant background exemplify that, Ashwagandha-root extract treatment can bring down lysosomal dysregulation induced necrosis of photoreceptor neurons. CONCLUSION: We have carried out a multifaceted study which elucidates that Ashwagandha can serve as a comprehensive, phytotherapeutic formulation to combat neurodegeneration, targeting multiple causative genetically defective conditions.				
Background: Cynara scolymus and Cichorium intybus are popular herbal remedy in folk medicine for liver disorders. Although many experimental studies carried out, scientifically reliable data needed to verify minimum effective dosage and efficacy of these medicinal plants. Objective: In present investigation, the effects of C. scolymus leaf and C. intybus root extracts at different doses were tested against CCl4 induced rats liver toxicity. Methods: The C. scolymus leaf and C. intybus root extracts at the doses of 300, 600 and 900 and 150, 300 and 450 mg/kg/day were prepared respectively. Liver intoxication was induced in 7 groups of rats by intraperitoneal injection of 1 ml/kg of 1:1 CCl4 in olive oil for two successive days. One group kept as control	1	0	1	0

and six different doses of plant extracts were administered to six groups simultaneously with CCl4 administration. The serum levels of ALT, AST and ALP, liver tissue glutathione and catalase activity as well as liver tissue microvesicular steatosis (MVS) and pericentral coagulation necrosis (PCN) were determined after three days. Results: The serum ALT, AST and ALP and liver tissue MVS were significantly reduced in both the C. scolymus and C. intybus groups at the doses of 900 and 450 mg/kg/day respectively while liver tissue PCN significantly reduced in C. scolymus 900 mg/kg/day group only as compared to control group. Conclusion: In present study administration of the C. scolymus leaf (900 mg/kg/day) and C. intybus root (450 mg/kg/day) extracts ameliorated CCl4 induced rat serum liver enzyme changes and liver tissue histopathological damage.				
Liver injury effects of green tea-based products have been reported in sporadic case reports. However, no study has examined systematically such adverse effects in an unbiased manner. We examined the potential effects of a high, sustained oral dose of green tea extract (GTE) on liver injury measures in a randomized, placebo-controlled, double-blinded phase II clinical trial, which enrolled 1,075 women with the original aim to assess the effect of daily GTE consumption for 12 months on biomarkers of breast cancer risk. The current analysis examined the effect of GTE consumption on liver injury in 1,021 participants (513 in GTE and 508 in placebo arm) with normal baseline levels of liver enzymes. Among women in the GTE arm, alanine aminotransferase (ALT) increased by 5.4 U/L [95% confidence interval (CI), 3.6-7.1] and aspartate aminotransferase increased by 3.8 U/L (95% CI, 2.5-5.1), which were significantly higher than those among women in the placebo arm (both P < 0.001). Overall, 26 (5.1%) women in GTE developed moderate or more severe abnormalities in any liver function measure during the intervention period, yielding an OR of 7.0 (95% CI, 2.4-20.3) for developing liver function	1	1	1	1

abnormalities as compared with those in the placebo arm. ALT returned to normal after dechallenge and increased again after one or more rechallenges with GTE. The rise-fall pattern of liver enzyme values following the challenge-dechallenge cycles of GTE consumption strongly implicates the effect of high-dose GTE on liver enzyme elevations.				
Seven new withanolides (1−7) and four known ones (8−11) were isolated from the calyxes and fruits of Nicandra physaloides, an edible and medicinal plant. Their structures were identified by extensive spectroscopic analyses or comparison with literature data. The antioxidant effects and hepatoprotective mechanisms of compounds 1–11 on hydrogen peroxide (H2O2)-induced oxidative stress injury were investigated. Among them, compound 11 showed significant activity with EC50 value of 83.78 ± 0.30 μM. Further, 11 was found to markedly increase cellular survivals and activities of SOD, CAT and GSH, and to reduce the accumulation of reactive oxygen species (ROS) in H2O2-induced LO2 cells. In addition, compound 11 significantly activated Nrf2 nuclear translocation and enhanced the expression of target gene HO-1, suggesting its preliminary mechanism of protective effects. In summary, withanolide 11 might be used as a lead compound for developing a hepatopathy medicine owing to its antioxidative and hepatoprotective effects.	0	1	0	0
Withania somnifera, commonly known as Ashwagandha, is a medicinal plant used for thousands of years for various remedies. Extracts of Ashwagandha contain more than 200 metabolites, with withanone (win) being one of the major ones responsible for many of its medicinal properties. Recently, several cases of liver toxicity resulting from commercially available Ashwagandha products have been reported. The first report of Ashwagandha-related liver damage was from Japan, which was quickly resolved after drug-withdrawal. Later, similar cases of liver toxicity due to Ashwagandha consumption were reported from the USA and Iceland. Towards	1	1	1	1

understanding the liver toxicity of Ashwagandha extracts, we studied win, a representative withanolide having toxicophores or structural alerts that are commonly associated with adverse drug reactions. We found that win can form non-labile adducts with the nucleosides dG, dA, and dC. Using various biochemical assays, we showed that win forms adducts in DNA and interfere with its biological property. Win also forms adducts with amines and this process is reversible. Based on the data presented here we concluded that win is detoxified by GSH but under limiting GSH levels it can cause DNA damage. The work presented here provides a potential mechanism for the reported Ashwagandha-mediated liver damage.				
Bisphenol A (BPA) safety aspects on human health are debated extensively for long time. In the present study, we have studied the toxicity induced by BPA at no observed adverse effect level (NOAEL) using HepG2 cells. We report that BPA at 100 nM induced cytotoxicity to HepG2 cells as determined by MTT assay at 0–72 h. The toxicity was result of reduced oxygen consumption and reduced mitochondrial membrane potential associated with decreased ATP production. The BPA treatment resulted in increase of malondialdehyde (MDA) content with decreased glutathione and other antioxidant enzymes. BPA derived toxicity is a concern to human health and alternative non-toxic natural products/derivatives or adjuvants that serve as antidote will be relevant. In this context, Ashwagandha (Withania somnifera) a widely used herb to treat arthritis, rheumatism and to improve longevity for time immemorial is investigated for its antidote effect. Ashwagandha supercritical CO2 extract derived Withanolides (ADW) at 100 µg/ml protect HepG2 cells from BPA induced toxicity by suppressing mitochondrial damage and increased ATP production. Further, cellular MDA content was significantly suppressed with increased non-enzymic and antioxidant enzyme activities. These findings derived from the present	0	0	1	0

study suggest the beneficial effect of ADW in mitigating BPA induced mitochondrial toxicity in HepG2 cells.				
The liver is the center for drug and xenobiotic metabolism, which is influenced most with medication/xenobiotic-mediated toxic activity. Drug-induced hepatotoxicity is common and its actual frequency is hard to determine due to underreporting, difficulties in detection or diagnosis, and incomplete observation of exposure. The death rate is high, up to about 10% for drug-induced liver damage. Endorsed medications represented >50% of instances of intense liver failure in a study from the Acute Liver Failure Study Group of the patients admitted in 17 US healing facilities. Albeit different studies are accessible uncovering the mechanistic aspects of medication prompted hepatotoxicity, we are in the dilemma about the virtual story. The expanding prevalence and effectiveness of Ayurveda and natural products in the treatment of various disorders led the investigators to look into their potential in countering drug-induced liver toxicity. Several natural products have been reported to date to mitigate the drug-induced toxicity. The dietary nature and less adverse reactions of the natural products provide them an extra edge over other candidates of supplementary medication. In this paper, we have discussed the mechanism involved in drug-induced liver toxicity and the potential of herbal antioxidants as supplementary medication.	0	1	1	0
This study aimed to investigate the possible protective role of clove oil against acrylamide induced oxidative damage and impairment of liver, kidney, and testicular functions in albino rats. The apparent oxidative damage was associated with evident hepatic, renal, and tes ticular dysfunction, which was confirmed in histopathological lesions, and increased serum aspartate aminotransferase and alanine ami notransferase activities. Acrylamide decreased serum total protein and albumin contents; increased urea and creatinine contents. Acryla mide also reduced testosterone concentration.	0	1	1	0

Treatment of acrylamide intoxicated rats with clove oil minimized liver, kidney, and tes ticular histopathological changes and normalized their functions. Our findings demonstrate that acrylamide is not only associated with hepatotoxicity but also nephrotoxicity and testicular toxicity. Clove oil administration provided substantial organ protection against he patic, renal, and testicular dysfunction induced by acrylamide, which was possibly mediated through their antioxidant activities.				
A common spice, Syzygium aromaticum is widely known as clove; this is the flower bud of a tree that belongs to the Myrtaceae family. With its origin in Indonesia, it has found application in medicine in Asian and western countries. It has taken a prominent place in alternative medicine and as a food flavoring agent. The clove buds contain several compounds of interest, such as eugenol, eugenyl acetate, trans-caryophyllene, β-caryophyllene, polyphenols, tannins, and triterpenoids. These rich bioactives make it a sought-after home remedy for illness from dental complications to inflammation of several kinds. The active compounds have been effectively extracted using solvents such as water, ethanol, and methanol. These compounds contribute to the fragrance and antioxidant, antibacterial, and antifungal properties, which underpin its enormous applications in the food and flavoring industries. Despite these beneficial properties, they may elicit some adverse reactions when administered at higher concentrations. Clove buds and their extracts containing active compounds, or the standalone compounds such as eugenol and oleoresins, have been approved by the Food and Drug Administration (FDA) as a food additive. The phenolics-rich fraction of clove has been reported to show no adverse effects on Wistar rats at 1000 mg/kg body weight/day. A few research reports are indicative of the extract affecting the reproductive indices in animal models. However, the studies related to the toxic exposure of clove extracts are limited due to the highly variable nature of the	0	1	1	1

sources and their constituents extracted thereafter. This work discusses the toxicity of different types of clove extracts.				
Euterpe oleracea Mart., Arecaceae, fruit (açaí) presents considerable potential for the development of new medicines due to its phytochemical composition and antioxidant activity. More recently, special attention has been given to the pharmacological potential of the fruit's oil. This study analysed the histological and histochemical effects of different dosages of açaí oil on rat's liver and thyroid cells, in order to evaluate its cytotoxic potential after administration for consecutive days. Male Wistar rats were treated with the açaí oil by gavage at doses of 30, 100 and 300 mg/kg, for 14 days, within a 24 h interval. Liver and thyroid fragments were collected for histology (hematoxylin and eosin) and histochemistry analysis (blue of Nilo (lipids), Baker (lipids), bromophenol blue (protein), PAS (polysaccharides)). The results showed that animals exposed to açaí oil presented alterations in the liver cells, where the integrity of the liver tissue was increasingly lost as the açaí oil doses increased. Nuclear pyknosis was observed in several hepatocytes, evidencing the occurrence of cell death. Alteration in the amount of lipids, polysaccharides, vacuoles in the cytoplasm, and proliferation of Kupffer cells were observed in histochemical analyzes. As for the thyroid of the treated rats, alterations were observed in the size of the follicular lumen and also in the connective tissue found between the follicles. Under the experimental conditions employed in the present study, the cytotoxicity observed in this work is worrying, specially considering the liver, when frequent or continuous damage could lead pathological disorders in this organ.	1	1	1	1
Maneb (MB) and paraquat (PQ) are environmental toxins that have been experimentally used to induce selective damage of dopaminergic neurons leading to the development of Parkinson's disease (PD). Although the mechanism of this selective neuronal toxicity in not fully understood, oxidative stress has been	0	0	0	0

linked to the pathogenesis of PD. The present study investigates the mechanisms of neuroprotection elicited by Withania somnifera (Ws), a herb traditionally recognized by the Indian system of medicine, Ayurveda. An ethanolic root extract of Ws was co-treated with the MB-PQ induced mouse model of PD and was shown to significantly rescue canonical indicators of PD including compromised locomotor activity, reduced dopamine in the substantia nigra and various aspects of oxidative damage. In particular, Ws reduced the expression of iNOS, a measure of oxidative stress. Ws also significantly improved the MB + PQ mediated induction of a pro-apoptotic state by reducing Bax and inducing Bcl-2 protein expression, respectively. Finally, Ws reduced expression of the pro-inflammatory marker of astrocyte activation, GFAP. Altogether, the present study suggests that Ws treatment provides nigrostriatal dopaminergic neuroprotection against MB-PQ induced Parkinsonism by the modulation of oxidative stress and apoptotic machinery possibly accounting for the behavioural effects.				
Atractylis gummifera L. (Asteraceae) is a thistle located in the Mediterranean regions. Despite the plant's well-known toxicity, its ingestion continues to be a common cause of poisoning. The toxicity of Atractylis gummifera resides in atractyloside and carboxyatractyloside, two diterpenoid glucosides capable of inhibiting mitochondrial oxidative phosphorylation. Both constituents interact with a mitochondrial protein, the adenine nucleotide translocator, responsible for the ATP/ADP antiport and involved in mitochondrial membrane permeabilization. Poisoned patients manifest characteristic symptoms such as nausea, vomiting, epigastric and abdominal pain, diarrhoea, anxiety, headache and convulsions, often followed by coma. No specific pharmacological treatment for Atractylis gummifera intoxication is yet available and all the current therapeutic approaches are only symptomatic. In vitro experiments	1	1	1	1

showed that some compounds such as verapamil, or dithiothreitol could protect against the toxic effects of atractyloside, but only if administered before atractyloside exposure. New therapeutic approaches could come from immunotherapy research: some studies have already tried to produce polyclonal Fab fragments against the toxic components of Atractylis gummifera.				
Introduction. In Mediterranean countries, intoxication by Atractylis gummifera L. is frequent and characterized principally by hepatorenal injury, often fatal. Its toxicity after a cutaneous application is unknown. We report a case of poisoning by A. gummifera L. induced by repeated cutaneous application. Case report. A 30-month-old boy was admitted in our pediatric intensive care unit in coma (Glasgow Coma Scale 8). Investigations showed hepatic cellular injury, cholestasis, decreased prothrombin level, and increased creatinine. History from the parents revealed repeated and occlusive cutaneous application of A. gummifera L. on a skin burn. Qualitative analysis of urine confirmed the diagnosis of A. gummifera poisoning. The child was discharged after 16 days of hospitalization with residual renal insufficiency. Discussion. Poisoning by A. gummifera L. after cutaneous application has not previously been reported in the literature. The prevention of this poisoning, particularly frequent in Mediterranean countries, is mainly based on the education of the public concerning the dangers of this plant.	1	1	1	1
Atractylis gummifera L. belongs to the family Asteraceae is widely used in traditional Moroccan medicine for its therapeutic effects (diuretic, purgative, emetic, abortive), but it causes serious and fatal poisonings, hence the objective of this work is to describe the current state of intoxication caused by A. gummifera in the Mediterranean and to summarize the toxicological studies carried out on this plant. The working methodology we adopted consisted in collecting data published in Arabic, French and English in specialized articles, books and on websites.	1	1	1	1

Research results showed that the Centre Anti Poison and Pharmacovigilance of Morocco declared A. gummifera was in second place in the occurrence of poisonings in between January 1980 and December 2008. The synthesis of experimental work on plant toxicology showed that the lethal dose of A. gummifera varies according to the animal model used (rat or mouse), the route of administration (intraperitoneal, oral or intravenous) and the part of the plant used. The root has been found to be the most toxic part of the plant. The toxicity of A. gummifera is due to atractyloside and gummiferine, which are inhibitors of oxidative phosphorylation that prevent the formation of ATP from ADP in intracellular organelles. This study shows the interest in raising public awareness of the toxicity of A. gummifera and in rationalizing its use in traditional medicine.				
Atractylis gummifera is a toxic plant widely used in Mediterranean traditional medicine against colds, dizziness, and headaches, as an antisyphilitic, against boils, as a purgative, emetic and deworming. All studies reported on this plant have been carried out either on the plant and its traditional uses, or on cases of poisoning by this plant. However, few pharmacological studies have readjusted the traditional uses of this plant.	1	0	1	1
In Morocco, acute Atractylis gummifera L. poisoning represents the leading cause of death by plant poisoning especially for children. All cases received in the Moroccan poison control centre from January 1981 to December 2009 (n = 467) were included in a retrospective study of the characteristics and risk factors of A. gummifera L. poisoning The most vulnerable age group was children (63.4% of cases). Most cases were due to accidental exposure (75.5%), but some were from therapeutic use (18.1%) or attempted abortion (7.4%). Patients presented with moderate poison severity signs (grade 2) in 22.3% of cases or severe signs (grade 3) in 21.0%. The mortality rate was 39.2%. The majority of deaths (81.1%) occurred in children aged <	1	1	1	1

15 years following accidental exposure. Multivariate logistic regression analysis revealed that risk factors for mortality were coma (OR = 20.5); hepatitis (OR = 52.7) and rural residence (OR = 7.26), while gastric decontamination was a protector factor (OR = 0.26).				
Atractyloside poisoning is an infrequent but often fatal form of herbal poisoning, which occurs worldwide but especially in Africa and the Mediterranean regions. The primary mechanism of atractyloside poisoning is known to be inhibition of the mitochondrial ADP transporter. Poisoning in humans may present with either acute hepatic or renal pathology and it is possible that there is a second, different mechanism of toxicity to the hepatocyte. Atractyloside in large amounts gives rise to massive necrosis, but in vitro studies have shown that at lower doses cells progress to apoptosis. Simple methods for the detection of atractyloside poisoning are at present restricted to thin-layer chromatography in urine and are useful only in the case of severe poisoning. Immunoassays, high-performance liquid chromatography, nuclear magnetic resonance, and a recently developed high-performance liquid chromatogrpahy/mass spectrometry method have yet to be applied to clinical diagnoses. There is at present no treatment, but a fuller understanding of the mechanisms of toxicity may lead to the application of a number of compounds that are effective in vitro.	1	1	1	1
Withania somnifera (L.) Dunal known as Ashwagandha is commonly used in traditional Indian medicine system. It possesses immense therapeutic value against a large number of ailments such as mental diseases, asthma, inflammation, arthritis, rheumatism, tuberculosis, and a variety of other diseases including cancer. The therapeutic potential of W. somnifera is due to the presence of secondary metabolites mainly, tropane alkaloids and withanolides (steroidal lactones). The growing realization of commercial value of the plant has initiated a new demand for in	0	0	0	0

vitro propagation of elite chemotypes of Withania. Micropropagation which is an important tool for rapid multiplication requires optimization of number of factors such as nutrient medium, status of medium (solid and liquid), type of explant, and plant growth regulators. Similarly, an efficient and reproducible in vitro regeneration system which is a prerequisite for the development of genetic transformation protocol requires precise manipulation of various intrinsic and extrinsic factors.				
In this report we describe a young, previously healthy woman who developed severe acute hepatitis after consumption of chaparral tablets, a commonly used herbal product. In this case, the elimination-rechallenge event and the exclusion of other possible aetiologic factors strongly supported true causality between the herbal product and the liver damage. Primary liver biopsy showed severe toxic hepatitis consistent with previous reports of chaparral-induced liver damage. Later, 6 months after the liver function tests had normalized, permanent hepatic fibrosis could still be seen.	1	1	1	1
Two patients with hepatic injury after ingestion of chaparral leaf are presented. The first patient, a 71-yr-old man, developed biopsy-proven hepatitis 3 months after ingesting chaparral leaf daily. His illness resolved with discontinuation of the herb and later recurred with rechallenge. The second patient is a 42-yr-old woman who developed hepatitis 2 months after chaparral leaf ingestion and recovered completely after discontinuation of the compound. Both patients have remained well with abstinence from chaparral. These reports provide evidence of the hepatotoxicity of this herb and stress the need for awareness of the potential harm from such nonprescription remedies.	1	1	1	1
Although Sophorae radix (SR) has been traditionally used as a treatment for various clinical symptoms, a comprehensive investigation of its safety has not yet been carried out. Therefore, we present an evaluation of the toxicity of the SR extract that was performed according to the	1	1	1	1

Organization for Economic Cooperation and Development test guidelines for subchronic toxicity and genotoxicity. In an oral subchronic study for 13 weeks, the repeated treatment of rats with 429 or 1500 mg/kg of the SR extract induced a dose-related change in body weight. In particular, the SR extract was observed to exert a significant increase in liver weight along with an increase in serum alkaline phosphatase and alanine transaminase. A small but statistically significant reductions in red blood cell, hemoglobin, and hematocrit levels in the SR extract-treated rats suggest the possibility that anemia, accompanied by liver injury, was at least partially induced. These findings indicate the no-observed-adverse-effect-level for the SR extract was considered to be 10 mg/kg/d. And, the data obtained from the chromosome aberration assay showed that SR extract might be considered to be a weak clastogen although no significant micronucleus induction was observed in vivo. Despite the benefits that SR extract can exhibit, this study indicates that SR extract may possess hepatotoxic and genotoxic potential.				
The three purgative Cheng-Chi-Tang decoctions (CCTDs) including Ta-Cheng-Chi-Tang (TCCT), Xiao-Chen-Chi-Tang (XCCT), and Tiao-Wei-Chen-Chi-Tang (TWCCT) are used for treating gastrointestinal disorders, including liver diseases in traditional Chinese medicine. However, the underlying mechanisms as liver disease remedies are far from fully clarified. The objective of the study is to investigate and compare the antioxidant activity of the three purgative CCTDs in order to delineate their hepatic protective potential and mechanism. Antioxidant activity measured with the 1,1-diphenyl-2-picrylhydrazyl (DPPH) radical scavenging test indicated XCCT as the most potent preparation (IC50 8.94 µg/ml). In tert-butylhydroperoxide (TBH, 50 mM)-induced lipid peroxidation in ICR mice liver homogenates, XCCT also showed stronger and dose-dependent inhibitory activity against TBH-induced malondialdehyde (MDA, a marker of lipid peroxidation)	1	0	0	0

production (IC50 53.66 µg/ml). In addition, XCCT showed dose-dependent protective effect against TBH-induced cytotoxicity in normal human Chung liver cells Furthermore, in carbon tetrachloride (CCl4)-induced acute liver injury model, mice pretreated with 0.2 g/kg and 0.4 g/kg of XCCT extracts showed a decrease of 59.8 and 43.1% in serum glutamic oxaloactetic transaminase (GOT) level, 51.4 and 52% in glutamic pyruvate transaminase (GPT) level, along with a reduction of 31 and 15% in MDA level, respectively, similar to the effects exerted by silymarin. XCCT pretreated mice also showed milder necrotic changes in the microscopic picture of the liver. The results suggest that XCCT has significant antioxidant activity and hepatic protection potential.				
Herbal medication has gathered increasing recognition in recent years with regard to both treatment options and health hazards. Pyrrolizidine alkaloids have been associated with substantial toxicity after their ingestion as tea and in the setting of contaminated cereals have led to endemic outbreaks in Jamaica, India and Afghanistan. In Western Europe, comfrey has been applied for inflammatory disorders such as arthritis, thrombophlebitis and gout and as a treatment for diarrhoea. Only recently was the use of comfrey leaves recognized as a substantial health hazard with hepatic toxicity in humans and carcinogenic potential in rodents. These effects are most likely due to various hepatotoxic pyrrolizidine alkaloids such as lasiocarpine and symphytine, and their related N-oxides. The mechanisms by which toxicity and mutagenicity are conveyed are still not fully understood, but seem to be mediated through a toxic mechanism related to the biotransformation of alkaloids by hepatic microsomal enzymes. This produces highly reactive pyrroles which act as powerful alkylating agents. The main liver injury caused by comfrey (Symphytum officinale) is veno-occlusive disease, a non-thrombotic obliteration of small hepatic veins leading to cirrhosis and eventually liver failure. Patients may present with either acute or	1	1	1	1

chronic clinical signs with portal hypertension, hepatomegaly and abdominal pain as the main features.				
Three groups of young adult rats were fed pyrrolizidine alkaloids derived from Russian comfrey to study the effects of the herb on the liver. Group I animals received a single dose of 200 mg/kg body wt, Group II 100 mg/kg three times a week for 3 weeks and Group III 50 mg/kg three times a week for 3 weeks. All rats showed light and electron-microscopic evidence of liver damage, the severity of which was dose dependent. There was swelling of hepatocytes and hemorrhagic necrosis of perivenular cells. There was a concomitant loss of sinusoidal lining cells with disruption of sinusoidal wall and the sinusoids were filled with cellular debris, hepatocyte organelles and red blood cells. Extravasation of red blood cells was evident. Terminal hepatic venules were narrowed by intimal proliferation, and in Group II and III, reticulin fibres radiated from these vessels. These appearances have been described in veno-occlusive disease due to pyrrolizidine alkaloids from other plant sources such as Senecio and Crotalaria. The safety of comfrey, a widely used herb, in relation to human consumption requires further investigation.	1	1	1	1
Five cases of liver injury attributed to ashwagandha-containing supplements were identified; three were collected in Iceland during 2017-2018 and two from the Drug-Induced Liver Injury Network (DILIN) in 2016. Other causes for liver injury were excluded. Causality was assessed using the DILIN structured expert opinion causality approach.	1	0	1	1
Liver is an essential metabolic organ. It can be damaged due to prolonged use and higher doses of drugs, exposure to some chemicals, toxins, or infectious agents. Herbal plants as ashwagandha (Withania somnifera) may have free radical scavenging activity thereby can be used for the prevention and treatment of liver damage. Objective: To observe the effect of ashwagandha (Withania somnifera) root extract on gentamicin induced changes of some liver marker enzymes e,g serum	0	0	0	0

aspartate amino transferase (AST) and alanine amino transferase (ALT) in Wistar albino rats. Methods: This experimental study was carried out in the Department of Physiology, Sir Salimullah Medical College (SSMC), Dhaka from 1st July 2010 to 30th June 2011. A total number of 35 Wistar albino rats, aged 90 to 120 days, weighing between 150 to 200 grams were selected for the study. After acclimatization for 14 days, they were divided into control group (Group A) and experimental group (Group B). Control group was again subdivided into group A1 (baseline control, consisted of 10 rats) and group A2 (gentamicin treated control group, consisted of 10 rats). Again, experimental group (Group B-ashwagandha pretreated and gentamicin treated group) consisted of 15 rats. All groups of animals received basal diet for 22 consecutive days. In addition to this, group A2 also received gentamicin subcutaneously (100mg /kg body weight/day) for the last eight (15th to 22nd day) consecutive days. Again, group B received ashwagandha root extract (500mg/kg body weight/day, orally) for 22 consecutive days and gentamicin subcutaneously (100mg/kg body weight /day) for last eight (15th to 22nd day) days. All the animals were sacrificed on 23rd day. Then blood and liver samples were collected. For assessment of liver function, serum AST, ALT and bilirubin levels were estimated. All these tests were done by standard Laboratory technique. The statistical analysis was done by one way ANOVA and Bonferroni test as applicable. Results: The mean serum levels of AST and ALT were significantly ($p<0.001$) higher in gentamicin treated control group and in ashwagandha pretreated and gentamicin treated group in comparison to those of baseline control group. . Again, these levels were significantly ($p<0.001$) lower in ashwagandha pretreated and gentamicin treated group than those of gentamicin treated control group. Conclusion: Ashwagandha (Withania somnifera) root extract restored serum AST, ALT towards normal levels in gentamicin intoxicated rats which may be due to its free radical				

scavenging activity. Therefore it may have hepatoprotective effect.				
This study was achieved to the safety evaluation of the ashwagandha (Withania somnifera) and total phenolic acid and total flavonoids compounds were determined, as well as ABTS and DPPH radical scavenging activities assay were studied. In addition, the biological experimental and histological assay was an examination of hepatitis rats. The results observed that the ashwagandha root extract was safety due to do not evidence of toxic effect or mortality in mice. This safety from the ashwagandha root extract caused the extract had contained high amounts of phenolic and flavonoids compounds which directly role of in free radical scavenging, thus the extract from ashwagandha root is a source of antioxidant activity. At the end of biological experimental after eight weeks the results were found that the effects of ashwagandha roots extract on carbon tetrachloride CCl_4-treated induced alterations in serum hepatic enzymes were decreased than control CCl_4 positive and give nearly equal control negative at level 400mg/kg body weight for a rat. Moreover, the results found that the effect ashwagandha roots extract on antioxidant enzymes hepatic as glutathione (GSH), superoxide dismutase (SOD), malondialdehyde (MDA), glutathione peroxidase (GPx), and catalase (CAT) parameters showed that the ashwagandha roots extract was improvement the antioxidant enzymes. Furthermore, different concentrations extract alleviated histopathological changes in rats' liver treated with CCl_4. Ashwagandha root extracts confirmed that the protection of the rats' liver CCl_4- induced hepatotoxicity. This influence may be due to activated, the antioxidant activities of these extracts. In recommending that the obviously results supported that the possible anti hepatic damage effect of ashwagandha root extract against CCl_4-induced hepatic damage. This anti hepatic damage influence may be due to activated, the antioxidant activities	0	0	0	0
The study findings demonstrated that the herbal tea of M. stenopetala and M. spicata	0	0	0	0

leaves blend could be relatively safe/low toxic to pregnant rats and developing fetuses. The no-observed-adverse-effect level (NOAEL) of herbal tea for maternal toxicity, fetotoxicity, and teratogenicity in rats is estimated to be > 2237.44 mg/kg/day.				
The experiments were undertaken to evaluate the effects of herbal medicine, Bojungiggitang and Gwibitang in pregnant rats and their fetuses. Female Sprague-Dawley rats were orally administered with the Bojungiggitang and Gwibitang at dose of 5ml/kg/day for 20 days. Pregnant rats were sacrificed at 20th day of gestation, and the internal and reproductive organs. Approximately live fetuses in the 20th day of gestation were randomly selected and fixed in 95% ethanol. To observe skeletal malformations, fetuses were stained with alcian blue and alizarin red S. Maternal body weights of Bojungiggitang and Gwibitang treated group has a tendency to increase compared to that of control group. There were no significant differences in internal and reproductive organs. There were no significant changes between two groups in blood chemistry and hematological values. There were no significant changes in number of corpus luteum, implantation and live fetuses. But Bojungiggitang and Gwibitang administered group showed higher implantation rate than the control group. Also, Bojungiggitang and Gwibitang administered groups showed lower early resorption rate than the control group. And Gwibitang had the higher value in all the other groups in all items. From the sex ratio, the number of females were larger than the number of males in the control group, and more males than females in Gwibitang administered group. Neonatal body weight and the number of fetus of Bojungiggitang and Gwibitang group were higher than that of control group. The fetuses of dams treated with Bojungiggitang and Gwibitang did not show external malformation. Vertebral and sternal variations were observed in Bojungiggitang and Gwibitang administered group compared to the control group.	0	0	0	0

Those variations were insignificant. There were no significant changes in number of ribs, cervical, thoracic, lumbar, sacral and caudal vertebras. From these results, it can be concluded that Bojungiggitang and Gwibitang showed no toxic effects on maternal body weight and the number of live fetuses. There were no significant changes in organ weight, hematological data, and reproductive organs. Although skeletal variations were shown in vertebra and sternum, Bojungiggitang, Gwibitang were shown insignificant changes in bone malformation.				
Twenty of the most commonly used Chinese herbal medicines prescribed for pregnancy were selected and the crude extract was administered to pregnant mice at clinical doses during five different gestational stages, namely post-implantation, gastrulation, organogenesis, maturation and whole gestation periods. Maternal effects on side effects, weight loss, litter reduction, implantation failure and fetal resorption and perinatal effects on growth restriction, developmental delay, congenital malformations and post-natal mortality were determined. Adverse pregnancy outcomes were commonly observed after maternal exposure to the herbal medicines, particularly during early pregnancy. Major events included maternal and perinatal mortality were recorded. Maternal weight gain, embryo growth and post-natal weight gain were significantly decreased. Fetal resorption and skeletal malformations were significantly increased.	1	0	1	1
Artemisinin compounds are important for treating multidrug-resistant malaria; however, the possible resorption and abnormalities observed in animal reproduction studies may contraindicate artemisinin use during the first trimester. To evaluate whether artemisinin interferes with developmental outcomes at different periods of pregnancy, Wistar rats were treated by gavage with increasing doses of 7, 35 and 70 mg/kg/day from gestational day [GD] 7 to 13 or 14 to 20. Viable embryos and post-implantation losses, and progestagens and testosterone levels, were	1	1	1	1

monitored in the former treatment group and pregnancy and outcomes data, post-implantation losses and male and female developmental endpoints of the offspring were evaluated in the latter treatment group. Results indicate toxicity for both periods of treatment, with lower sensitivity at later stages of pregnancy. The results showed that dosing with 35 or 75 mg/kg of artemisinin caused high percentages of post-implantation losses that correlated with a trend to lower maternal progestagens and a significant maternal testosterone decrease. These findings demonstrate that oral administration of artemisinin can adversely effect post-implantation development and pregnancy in the rat.				
Arsenic is a harmful and toxic substance to the growth and development of plants. Salicylic acid (SA) acts as a signaling molecule, plays pivotal roles in the overall growth and development of plants under various environmental stresses. Artemisinin extracted from the leaves of A. annua helps in malarial treatment. The present investigation is aimed to find out the possible ameliorative role of exogenously-applied salicylic acid (SA) on two varieties of Artemisia annua L., namely ‘CIM-Arogya’ and ‘Jeevan Raksha’ under arsenic (As) stress conditions. For this, growth, physiological and biochemical characterization, and artemisinin production was assessed. The various treatments applied on the plants were Control, 10−6 M SA, 10−5 M SA, 45 mg kg−1As, 45 mg kg−1 As + 10−6 M SA, and 45 mg kg−1 As + 10−5 M SA. Arsenic at 45 mg kg−1 of soil, reducing the overall performance of both varieties at 90 and 120 DAP. However, the levels of antioxidants were enhanced in As-stressed plants, and the supplementation of SA further increased these antioxidants in SA-treated plants. It has been observed that minimum reduction in growth and yield occurs with enhanced production of artemisinin in the case of ‘CIM-Arogya’ compared to ‘Jeevan Raksha’ under As stress (45 mg kg−1 of soil). Leaf-applied SA significantly increased the	0	0	1	0

content (49.0% & 43.4%) and yield (53.3% & 46.3%) of artemisinin in both tolerant and sensitive varieties as compared to their respective controls. Thus, the variety 'CIM-Arogya' showed tolerant behavior over 'Jeevan Raksha' and is much adapted to higher As stress.				
A common spice, Syzygium aromaticum is widely known as clove; this is the flower bud of a tree that belongs to the Myrtaceae family. With its origin in Indonesia, it has found application in medicine in Asian and western countries. It has taken a prominent place in alternative medicine and as a food flavoring agent. The clove buds contain several compounds of interest, such as eugenol, eugenyl acetate, trans-caryophyllene, β-caryophyllene, polyphenols, tannins, and triterpenoids. These rich bioactives make it a sought-after home remedy for illness from dental complications to inflammation of several kinds. The active compounds have been effectively extracted using solvents such as water, ethanol, and methanol. These compounds contribute to the fragrance and antioxidant, antibacterial, and antifungal properties, which underpin its enormous applications in the food and flavoring industries. Despite these beneficial properties, they may elicit some adverse reactions when administered at higher concentrations. Clove buds and their extracts containing active compounds, or the standalone compounds such as eugenol and oleoresins, have been approved by the Food and Drug Administration (FDA) as a food additive. The phenolics-rich fraction of clove has been reported to show no adverse effects on Wistar rats at 1000 mg/kg body weight/day. A few research reports are indicative of the extract affecting the reproductive indices in animal models. However, the studies related to the toxic exposure of clove extracts are limited due to the highly variable nature of the sources and their constituents extracted thereafter. This work discusses the toxicity of different types of clove extracts.	0	1	1	1
Despite the various reports on the toxicity of clove oil and its major component	0	1	0	0

eugenol, systematic evaluations on the safety of polyphenolic extracts of clove buds have not been reported. Considering the health beneficial pharmacological effects and recent use of clove polyphenols as dietary supplements, the present study investigated the safety of a standardized polyphenolic extract of clove buds (Clovinol), as assessed by oral acute (5 g/kg b.wt. for 14 days) and subchronic (0.25, 0.5 and 1 g/kg b.wt. for 90 days) toxicity studies on Wistar rats and mutagenicity studies employing Salmonella typhimurium strains. Administration of Clovinol did not result in any toxicologically significant changes in clinical/behavioural observations, ophthalmic examinations, body weights, organ weights, feed consumption, urinalysis, hematology and clinical biochemistry parameters when compared to the untreated control group of animals, indicating the no observed-adverse-effect level (NOAEL) as 1000 mg/kg b.wt./day; the highest dose tested. Terminal necropsy did not reveal any treatment-related histopathology changes. Clovinol did not show genotoxicity when tested on TA-98, TA-100 and TA-102 with or without metabolic activation; rather exhibited significant antimutagenic potential against the known mutagens, sodium azide, NPD and tobacco as well as against 2-acetamidoflourene, which needed metabolic activation for mutagenicity.				
Different mechanisms of action have been proposed to explain cytotoxicity of eugenol: (a) oxidation of eugenol by peroxidases generates quinone methide intermediate which is toxic to hepatocytes; (b) high affinity of eugenol to plasma membranes due to its highly lipophilic nature results in cell damage; (c) the influence of eugenol on uncoupling of oxidative phosphorylation in mitochondria; (d) the prooxidant activity of eugenol triggers the formation of oxygen-free radicals that contribute to tissue damage; and (e) protein deactivation and consequently toxicity due to binding of lysine to eugenol. Nevertheless, more detailed in vitro and in vivo studies are still	1	1	1	1

required to clearly reveal the mechanism of action of clove's toxicity on human health.				
Comfrey has been consumed by humans as a vegetable and a tea and used as an herbal medicine for more than 2000 years. Comfrey, however, produces hepatotoxicity in livestock and humans and carcinogenicity in experimental animals. Comfrey contains as many as 14 pyrrolizidine alkaloids (PA), including 7-acetylintermedine, 7-acetyllycopsamine, echimidine, intermedine, lasiocarpine, lycopsamine, myoscorpine, symlandine, symphytine, and symviridine. The mechanisms underlying comfrey-induced genotoxicity and carcinogenicity are still not fully understood. The available evidence suggests that the active metabolites of PA in comfrey interact with DNA in liver endothelial cells and hepatocytes, resulting in DNA damage, mutation induction, and cancer development. Genotoxicities attributed to comfrey and riddelliine (a representative genotoxic PA and a proven rodent mutagen and carcinogen) are discussed in this review. Both of these compounds induced similar profiles of 6,7-dihydro-7-hydroxy-1-hydroxymethyl-5H-pyrrolizine (DHP)-derived DNA adducts and similar mutation spectra. Further, the two agents share common mechanisms of drug metabolism and carcinogenesis. Overall, comfrey is mutagenic in liver, and PA contained in comfrey appear to be responsible for comfrey-induced toxicity and tumor induction.	1	1	1	1
Corydalis yanhusuo W. T. Wang (Papaveraceae) is a traditional Chinese herbal medicine that has long been used to treat several conditions and is widely distributed in Asian countries. This review focuses on the traditional uses, botany, phytochemistry, pharmacology, pharmacokinetics, and toxicology of C. yanhusuo. The literature on C. yanhusuo was reviewed using several resources, including classic books on Chinese herbal medicine and scientific databases, namely, PubMed, Springer, Web of Science, Science Direct, and China National Knowledge Infrastructure. Based on information from	0	1	0	1

these databases regarding the chemical components of C. yanhusuo, we evaluated the underlying interaction network between chemical components, biological targets, and associated diseases using Cytoscape software. To date, more than 160 compounds have been isolated and identified from C. yanhusuo, including alkaloids, organic acids, volatile oils, amino acids, nucleosides, alcohols, and sugars. The crude extracts and purified compounds of this plant have analgesic, antiarrhythmic, and antipeptic ulcer properties, along with hypnotic effects. However, studies on the pharmacokinetics of C. yanhusuo extracts remain limited. C. yanhusuo has therapeutic potential in diseases such as cancer and depression, probably due to glaucine and corydaline. Our network pharmacology analysis revealed interactions between 20 compounds, 54 corresponding targets, and 4 health conditions. We found that leonticine, tetrahydroberberine, and corydalmine may regulate the expression of PTGS2, PTGS1, KCNH2, SCN5A, RXRA, CAMKK2, NCOA2, and ESR1, representing a potential treatment strategy against pain, gastric ulcers, inflammation, and cardiac arrhythmias. Additionally, this article discusses the future directions of research on C. yanhusuo.				
Ambinine, the major alkaloid of the tuber of Corydalis ambigua var. amurensis, has protective effects on H9C2 myocardial cells. In the present paper, we observed that ambinine demonstrates activities of both anticoagulation and thrombolysis in vitro by significantly degrading the blood clot and delaying the plasma recalcification time (PRT) in a dose-dependent manner (0.5–2 mg/mL). We further studied its safety profile of acute and subacute toxicity by repeated-dose intravenous injection. The median lethal dosage (LD50) of mice given by oral and intravenous administration of ambinine were approximate 800, 41.60 mg/kg, respectively. The acute toxicity research results suggested that compared with an intravenous administration, the oral route is safer to administer ambinine as the promising lead compound for thrombosis.	1	1	1	1

In subacute toxicity research, when mice were given ambinine at doses of 1.40 and 2.10 mg/kg for 7 days by injection, significant alteration of the relative kidney weight, the relative liver weight and serum biochemistry parameters and marked histopathological changes of them were found.				
Corydalis is an herbal plant found in Asian countries. Research has demonstrated multiple health benefits. It has also been implicated in drug-induced liver injury. Cannabis dispensaries market a sleep aid which has corydalis as an active ingredient. We present 2 cases of corydalis-induced hepatotoxicity. An asymptomatic female patient exhibited a rise and fall of her transaminases coinciding with the consumption and rechallenge of this sleep aid. A man with symptoms consistent with liver dysfunction began taking the same sleep aid. With discontinuance, his liver function returned to normal. These 2 clinical cases provide evidence for corydalis-induced liver injury.	1	1	1	1
Echinacea exhibited excellent activities in resisting a variety of hepatopathy induced by different causes in preclinical experiments and clinical trials by regulating cell proliferation and apoptosis, antioxidant defense mechanism, voltage-gated sodium channels, lipid metabolism, circadian rhythm, p38 MAPK signaling pathway, JNK signaling pathway, Nrf2/HO-1 signaling pathway, PI3K/AKT signaling pathway, and Akt/GSK3 beta signaling pathways. The high efficacy of Echinacea is related to its immunomodulatory and anti-inflammatory activities. The main ingredients of Echinacea include caffeic acid derivatives, alkylamides, and polysaccharides, which have been well established in preclinical studies of liver diseases. Studies on acute and subacute toxicity show that Echinacea preparations are well-tolerated herbal medicines.	0	0	0	0
Echinacea preparations are widely used in the prevention or treatment of upper respiratory tract infections. The present study aimed to investigate the effect of a standardized Echinacea extract in	0	1	1	0

experimentally induced liver toxicity and whether this herb would have a modulating effect on the silymarin-induced hepatoprotection in rats. Liver damage was induced by the administration of carbon tetrachloride (CCl4). Echinacea extract (18 or 36 mg/kg) alone or combined with silymarin (25 mg/kg), silymarin only (25 mg/kg), or saline (control) was given once daily orally simultaneously with CCl4 and for 1 week thereafter. Serum alanine aminotransferase (ALT) or aspartate aminotransferase were not significantly changed by treatment with Echinacea, but alkaline phosphatase (ALP) in serum decreased by 23.6% by the extract at 36 mg/kg. Silymarin given in combination with either dose of Echinacea resulted in 34.9% and 57.8% reduction in AST, 42.7% and 58% reduction in ALT and 41% and 60% reduction in ALP, compared with CCl4 control group. Silymarin alone reduced ALT, AST, and ALP levels by 58.8%, 61.2%, and 62.2%, respectively. Histopathological examination revealed a mild decrease in degenerated hepatocytes after treatment with 36 mg/kg of Echinacea. Noticeable improvement in the liver damage was observed upon the addition of silymarin to Echinacea. A marked decrease of intracellular protein and glycogen staining was evident after the administration of CCl4. Slight improvement in protein and glycogen staining was noted after 36 mg/kg of Echinacea. Increased hepatic protein and glycogen staining intensity was observed after silymarin and Echinacea co-treatment compared with Echinacea-only treated groups. Silymarin only treatment resulted in more or less normal histopathological and histochemical findings. The present results suggest that administration of Echinacea extract reduces the hepatoprotective effect of silymarin. Such finding is likely to have important clinical significance in patients with hepatic disease on silymarin treatment.				
Echinacea purpurea, Echinacea angustifoli and Echinacea pallida are frequently used as medicinal plants. Besides asking for evidence on their efficacy, there is an	0	1	0	0

increasing interest for safety data. This review systematically presents the available literature on drug interactions, contraindications, adverse events, duration of use, and safety of use in pregnant and nursing women, and assesses the safety profile of corresponding Echinacea preparations. It is noteworthy that all safety data reported are as product specific as the pharmacological or efficacy data are. In pharmacokinetic herb-drug interaction studies performed in vivo, no significant inhibitions of human CYP2D6 and CYP3A4 isoforms have been found after the administration of standardized E. purpurea preparations. However, contradictory results exist in studies using liver microsomes. Adverse events reported during clinical trials following administration of Echinacea spp. mono-preparations were generally mild and mostly without causality. Due to published long term studies with continuous ingestion of different Echinacea preparations up to 6 month with no reported toxicological concerns, Echinacea can be recommended also for long-term use. Moreover, the contraindications in cases of autoimmune diseases and immune-suppression are questionable, since lipophilic Echinacea preparations containing alkamides suppress cellular immune responses, and beneficial effects in autoimmunity were reported. The same applies for the use during pregnancy. Although there has been some impact reported on embryonic angiogenesis in mice, no association with an increased risk for major or minor malformations during organogenesis was found in a literature review. Altogether, the different evaluated Echinacea preparations are well-tolerated herbal medicines in the management in children and adults alike.				
Ephedrine abuse has spread in many parts of the world and severely threatens human health. The mechanism of ephedrine-induced toxicity still remains unclear. This study was performed to investigate the effects of ephedrine treatment on the liver and explore the underlying mechanisms. Sprague Dawley rats were divided into	1	1	1	1

saline and ephedrine groups. Rats were treated with ephedrine at 20 mg/kg or 40 mg/kg (n = 10) by oral gavage daily for 7 days. Pathological changes were examined by hematoxylin and eosin staining and terminal deoxynucleotidyl transferase deoxyuridine triphosphate nick end labeling assay. Enzyme-linked immunosorbent assays were used to measure the liver functional markers, oxidative stress markers, and inflammatory cytokines. Real-time polymerase chain reaction and Western blot were used to measure gene and protein expression, respectively. Our data showed that ephedrine treatment increased hepatocellular cell apoptosis and impaired liver function. Moreover, ephedrine treatment increased oxidative stress and inflammatory responses, which may be due to the increase of transforming growth factor β (TGF-β)/Smad3 expression. Our study demonstrated that short-term treatment of ephedrine caused liver toxicity in rats through regulating TGF-β/Smad pathway.				
Cisplatin (CP) is a powerful anticancer agent used in the treatment of a diverse type of cancers. Oxidative stress is one of the most important side effects limiting the use of cisplatin. The protective effects of methanolic extract (ME) and ephedrine (EP), major compound, of Ephedra alata on CP-induced damages were here assessed. Treatment with CP-induced nephrotoxicity and hepatotoxicity characterized by biochemical alterations. In fact, using CP reduced significantly glutathione (GSH) levels, enzymatic activities of superoxide dismutase (SOD), catalase (CAT), glutathione-S-transferase (GST), and increased malondialdehyde (MDA) content. Nonetheless, CP-treatment induced DNA damage at renal, hepatic, and blood cells and increased interferon gamma (IFNγ) level in serum. Co-treatments of mice with ME normalized relative kidney/body weight, restored biochemical and oxidative stress parameters, reduced DNA damage and IFNγ level. In conclusion, ME exhibited the best protective effect against CP damage compared with ephedrine. This is could be	0	0	1	0

attributed to the presence of polysaccharides, organic acids, flavonoids, and tannins in addition to ephedrine alkaloids. These compounds were reported to play a major role in inhibiting and scavenging free radicals, providing an effective protection against CP- induced oxidative damage.				
Background: Ephedra sinica (ES) (Ma-huang) is a well-known plant due to its widespread therapeutic uses. However, many adverse effects such as hepatitis, nephritises, and cardiovascular toxicity have been reported for this plant. Few of these side effects are reversible whereas others are irreversible and may even lead to death. Aim of the Study: The aim of this study was to investigate the clinical uses and toxicity cases/consequences associated with the use of ES. The review will compare and evaluate the cases reported for ES and identify the causes which make the plant a poisonous one. Materials and Methods: An extensive literature review was conducted from 2004 to 2017, and research literature regarding the clinical cases were collected using databases and books such as Google Scholar, Science Direct, Research gate, PubMed, and Web of Science/Thomson Reuters whereas the keywords searched were "Ephedra sinica," clinical cases of Ephedra sinica, "Ma-hung poisonous," "Ma-hung toxicity reported cases and treatment," and "Ephedra Sinica toxicity reported cases and treatment." Results: eleven different cases were identified which met the eligibility criteria and were studied in detail to extract out the findings. It turned out that most of the patients and participants developed adverse side effects following the use of ES, and after a proper course of treatment or trail, some of the side effects were reversible, whereas others were found irreversible. The most common adverse effects and toxicity observed in patients were liver problems, renal failures, and cardiac toxicity which resulted in two mortalities whereas the other two got recovered. On an in-depth study, it was revealed that most of the toxicities in such cases were associated with the use of ES in	1	0	1	1

combination with other conventional drugs, long-term use, or use in patients with cardiac and renal problems without a medical supervision. Conclusion: Hence, it is concluded that ES may have reversible as well as irreversible adverse effects and may even result in death; however, the use of a plant with the proper knowledge and under sound medical supervision may reduce such exaggerations				
p-Synephrine is an adrenergic amine found in Citrus aurantium L. fruits and has been used for weight loss in dietary supplements. There are commercial products containing this substance associated to caffeine, salicin, and ephedrine. The aim of this study was to evaluate the acute toxicity of this mixture in mice of both sexes. The significative results observed after acute oral administration to male and female mice of 300, 350, and 400 mg/kg total of p-synephrine, ephedrine, salicin, plus caffeine in a 10:4:6:80 w/w ratio included a reduction in locomotor activity and ptosis in all treated groups for both sexes. Seizures were also observed in male (400 mg/kg) and female groups (350 and 400 mg/kg). Gasping and tearing were observed in males. Salivation (400 mg/kg), agitation (350 and 400 mg/kg), and piloerection (all treated groups) were significantly observed only in females. Deaths occurred in males at 350 and 400 mg/kg treated groups and the necropsy showed cardiopulmonary hemorrhage. A reduction in locomotor activity was confirmed through the spontaneous locomotor activity test, in which the number of crossings considerably decreased ($P < .01$) in all treated groups. The rotarod test showed a decrease in motor coordination at 400 mg/kg. Body temperature decreased significantly ($P < .01$) in all treated groups compared to controls. The results suggested clear signs of toxicity of p-synephrine, ephedrine, salicin, and caffeine association; this toxicity augments the attentiveness on commercial products containing this mixture, given the expressive number of adverse events related to its utilization.	1	1	1	1

BACKGROUND: Withania somnifera (family solanaceae) is a well-investigated medicinal plant which is also called Indian ginseng due to its wide spectrum of medicinal properties. The contents and activity of the plant may vary depending on the habitat and part of the plant and the solvent used for extraction. The present study deals with the comparative chemical analysis and in vitro antioxidant activity of methanolic fruits extracts and its subfractions (in ethyl acetate, butanol and water) of W. somnifera collected from two different geographical locations. METHODS: In the present study, Withania somnifera fruits were collected from two different geographical locations (Uttarakhand and Rajasthan). The different fruit extracts were prepared and studied for total phenolic contents and total flavone contents. The in vitro antioxidant activity was assessed by DPPH free radical scavenging assay and peroxide scavenging assay. RESULTS: Methanol extract of W. somnifera Uttarakhand and ethyl acetate subfraction of W. somnifera Rajasthan showed the highest amount of Total Phenolic Contents (TPC). In W. somnifera Uttarakhand, ethyl acetate extract showed the highest amount of Total flavonoids while in W. somnifera Rajasthan, methanol extract was found to be the richest in flavonoids. Methanolic extract of W. somnifera Uttarakhand showed the highest free radical scavenging activity while in W. somnifera Rajasthan, the highest antioxidant activity was shown by the methanolic extract followed by butanolic extract, water extract and then ethyl acetate. In the peroxide scavenging assay of antioxidant activity, water extract of W. somnifera Uttarakhand showed the highest activity, while in W. somnifera Rajasthan, ethyl acetate extract showed highest scavenging activity. CONCLUSION: It was concluded that the geographical location exerts a vital effect on the presence of active constituents and also on the antioxidant potential of W. somnifera.	0	0	0	0
Background. Herbal preparations are available widely and regarded generally by the public as harmless remedies for a	1	1	1	1

variety of medical ailments. We report a case of acute hepatitis associated with the use of kava kava, derived from the root of the pepper plant, Piper methysticum. It is used in the United States as an antianxiety and sedative agent. Case report. A previously healthy 14-year-old female was admitted to the hospital with hepatic failure. Initial therapy, including plasmapheresis, was unsuccessful and she deteriorated. She ultimately required a liver transplant and now remains well. The liver biopsy showed hepatocellular necrosis consistent with chemical hepatitis. A work-up for alternative causes of liver failure was negative. The patient gave a history of taking a kava kava–containing product for four months. The use of kava kava and liver failure, is supported by kava kava use, a negative work-up for alternative causes of liver failure, and histological changes in the liver. Conclusions. Health care professionals need to be aware of the possibility of kava kava–induced hepatotoxicity. The toxicity of these alternative remedies emphasizes the importance of surveillance programs and quality control in the manufacture of these products. Clinicians must remain aware of the toxic potential of herbal products and always inquire about their intake in cases of unexplained liver injury.				
Kava is a traditional beverage of various Pacific Basin countries. Kava has been introduced into the mainstream U.S. market principally as an anti-anxiety preparation. The effects of the long-term consumption of kava have not been documented adequately. Preliminary studies suggest possible serious organ system effects. The potential carcinogenicity of kava and its principal constituents are unknown. As such, kava extract was nominated for the chronic tumorigenicity bioassay conducted by the National Toxicology Program (NTP). At present toxicological evaluation of kava extract is being conducted by the NTP. The present review focuses on the recent findings on kava toxicity and the mechanisms by which kava induces hepatotoxicity.	1	1	1	1

Licorice is used as a medicinal plant, and several studies have shown that licorice has beneficial effects. The objective of this study was to evaluate the safety of nonpolar licorice extract using toxicity experiments. Nonpolar extract from the root of Glycyrrhiza uralensis (NERG) was analyzed by high-performance liquid chromatography (HPLC). Antioxidant ability was determined by method of TPC and DPPH. Blood pressure was monitored by using blood pressure meter. In the acute study, a single dose (2,000 mg/kg) was orally administered to mice. In the subchronic study, mice were treated with extract at doses (50, 100, 500, and 1,000 mg/kg) for 120 days. Significantly difference was not shown at blood pressure, hematological, and biochemical parameters, and histopathology on mice. The results suggested that at acute and subchronic toxicity, each levels of nonpolar licorice extract administration in experiments did not cause toxicity effects or death on mice.	0	0	0	0
Neem (Azadirachta indica A. Juss) is one of the tropical plants found in Indonesia that has been used to prevent and treat various diseases. This study aimed to investigate the effect of the ethanol extract of neem leaves on the concentration of aspartate aminotransferase (AST), alanine aminotransferase (ALT), urea, and creatinine in male rats. Twenty-four male Wistar rats were randomly divided into four groups (T0, T1, T2, and T3) with 6 rats in each group. T0 is the control group, and T1, T2, and T3 are the treatment groups that were administered 100, 200, and 300 mg/kg body weight of neem leaf ethanolic extracts for 48 days, respectively. On day 49, blood samples were collected to measure the concentration of AST, ALT, creatinine, and urea followed by an evaluation of liver and kidney histology. The results showed that the ethanolic extract of neem leaves did not affect the concentration of AST, ALT, and creatinine, The ethanol leaves reduced extract on the urea concentration, no abnormal changes were observed in the liver and kidney	0	1	0	0

organs. In the future, it is required to carry out a comprehensive safety evaluation of the neem leaf ethanol extract for herbal medicines.				
Noni juice (Morinda citrifolia) has been approved for use as a safe food within the European Union, following a review of safety. Since approval, three cases of acute hepatitis in Austrian noni juice consumers have been published, where a causal link is suggested between the liver dysfunction and ingestion of anthraquinones from the plant. Measurements of liver function in a human clinical safety study of TAHITIAN NONI® Juice, as well as subacute and subchronic animal toxicity tests revealed no evidence of adverse liver effects at doses many times higher than those reported in the case studies. Additionally, M. citrifolia anthraquinones occur in the fruit in quantities too small to be of any toxicological significance. Further, these do not have chemical structures capable of being reduced to reactive anthrone radicals, which were implicated in previous cases of herbal hepototoxicity. The available data reveals no evidence of liver toxicity.	0	1	0	0
AIM: NONI juice (Morinda citrifolia) is an increasingly popular wellness drink claimed to be beneficial for many illnesses. No overt toxicity has been reported to date. We present two cases of novel hepatotoxicity of NONI juice. Causality of liver injury by NONI juice was asses-sed. Routine laboratory tests and transjugular or percutaneous liver biopsy were performed. The first patient underwent successful liver transplantation while the second patient recovered spontaneously after cessation of NONI juice. A 29-year-old man with previous toxic hepatitis associated with small doses of paracetamol developed sub-acute hepatic failure following consumption of 1.5 L NONI juice over 3 wk necessitating urgent liver transplantation. A 62-year-old woman without evidence of previous liver disease developed an episode of self-limited acute hepatitis following consumption of 2 L NONI juice for over 3 mo. The most likely hepatotoxic components of Morinda citrifolia were anthraquinones. Physicians	1	1	1	1

should be aware of potential hepatotoxicity of NONI juice.				
Morinda citrifolia (noni) fruit juice has been approved as a safe food in many nations. A few cases of hepatitis in people who had been drinking noni juice have been reported, even though no causal link could be established between the liver injury and ingestion of the juice. To more fully evaluate the hepatotoxic potential of noni fruit juice, in vitro hepatotoxicity tests were conducted in human liver cells, HepG2 cell line. A subchronic oral toxicity test of noni fruit was also performed in Sprague-Dawley (SD) rats to provide benchmark data for understanding the safety of noni juice, without the potential confounding variables associated with many commercial noni juice products. Freeze-dried filtered noni fruit puree did not decrease HepG2 cell viability or induce neutral lipid accumulation and phospholipidosis. There were no histopathological changes or evidence of dose-responses in hematological and clinical chemistry measurements, including liver function tests. The no-observed-adverse-effect level (NOAEL) for freeze-dried noni fruit puree is greater than 6.86 g/kg body weight, equivalent to approximately 90 ml of noni fruit juice/kg. These findings corroborate previous conclusions that consumption of noni fruit juice is unlikely to induce adverse liver effect	0	0	0	0
This study evaluated the protective effects of Noni fruit juice on acute liver injury induced by carbon tetrachloride (CCl_4) in female Sprague-Dawley (SD) rats. Liver damage (micro-centrilobular necrosis) was observed in animals pretreated with 20% placebo (drinking water) + CCl_4. However, pretreatment with 20% Noni juice in drinking water + CCl_4 resulted in markedly decreased hepatotoxic lesions. Furthermore, serum alanine aminotransferase and aspartate aminotransferase levels were significantly lower in the Noni group than the placebo group. In a correlative time-dependent study, one dose of CCl_4 (0.25 mL/kg in corn oil, p.o.) in female SD rats, pretreated with	0	1	1	0

10% placebo for 12 days, caused sequential progressive hepatotoxic lesions over a 24 h period, while a protective effect from 10% Noni juice pretreatment was observed. These results suggest that Noni juice is effective in protecting the liver from extrinsic toxin exposure.				
Cancer is still considered a "hopeless case", besides all of the advancements in oncology research. On the other hand, the natural products, as effective lead molecules, have gained significant interest for research due to the absence of toxic and harmful side effects usually associated with conventional treatment methods. Medicinal properties of herbal plants are strongly evidenced in traditional medicine from ancient times. In the context above, withaferin A (WA) was identified as the active principle of the plant Withania somnifera, its molecule being reported to have excellent anticancer and tumour inhibition activities in various cell lines. Furthermore, the in silico approaches in the medicinal chemistry of WA revealed the biological targets and gave momentum for the research that leads to many amazing pharmacological activities of WA which are not yet explored. This includes a broad spectrum of anticancer actions manifested in different organs (breast, pancreas, colon), melanoma and B cell lymphoma, etc. This review is an extensive survey of the most recent anticancer studies reported for WA, along with its mechanism of action and details about its in vitro and/or in vivo behaviour.	0	0	1	0
popularity of Morinda Citrifolia Linn (Noni) as a dietary supplement, a food functional ingredient, or as a natural health enhancer is increasing throughout the world. Our study aims to investigate the concentrations of 10% and 50% of Morinda Citifolia Linn and usage time and its renal and hepatic toxicity. To this purpose, 42 male wistar rats were distributed into groups: treated for 15 days with concentration of 10% and 50and treated for 90 days with concentration of 10% and 50%. Noni juice has been prepared with fruit’s pulp and seeds at concentrations of 10% and 50% diluted in water. The groups 15 and 90 days presented	1	1	1	1

elevated creatinine, urea, AST and ALT, compared to respectively control groups (p<0,05), and the levels of these markers are higher in 90 days groups.The presence of steatosis and dermatological changes was observed in rats in 90-days and concentration 50%. The fruit juice of Morinda Citrifolia Linn at concentration of 50% causes damage to liver and kidney function, being able to induce development of non-alcoholic liver disease, regardless of usage time. The alterations of hepatic and renal markers are directly proportional to usage time and dose-concentration.				
Gliomas are the most frequent type of primary brain tumor in adults. Their highly proliferative nature, complex cellular composition, and ability to escape therapies have confronted investigators for years, hindering the advancement toward an effective treatment. Agents that are safe and can be administered as dietary supplements have always remained priority to be most feasible for cancer therapy. Withania somnifera (ashwagandha) is an essential ingredient of Ayurvedic preparations and is known to eliminate cancer cells derived from a variety of peripheral tissues. Although our previous studies have addressed the in vitro anti-proliferative and differentiation-inducing properties of ashwagandha on neuronal cell lines, in vivo studies validating the same are lacking. While exploring the mechanism of its action in vitro, we observed that the ashwagandha water extract (ASH-WEX) induced the G2/M phase blockade and caused the activation of multiple pro-apoptotic pathways, leading to suppression of cyclin D1, bcl-xl, and p-Akt, and reduced the expression of polysialylated form of neural cell adhesion molecule (PSA-NCAM) as well as the activity of matrix metalloproteinases. ASH-WEX reduced the intracranial tumor volumes in vivo and suppressed the tumor-promoting proteins p-nuclear factor kappa B (NF-kappaB), p-Akt, vascular endothelial growth factor (VEGF), heat shock protein 70 (HSP70), PSA-NCAM, and cyclin D1 in the rat model of orthotopic glioma allograft. Reduction in	0	0	0	0

glial fibrillary acidic protein (GFAP) and upregulation of mortalin and neural cell adhesion molecule (NCAM) expression specifically in tumor-bearing tissue further indicated the anti-glioma efficacy of ASH-WEX in vivo. Combining this enhanced understanding of the molecular mechanisms of ASH-WEX in glioma with in vivo model system offers new opportunities to develop therapeutic strategy for safe, specific, and effective formulations for treating brain tumors.				
Case Report: A 27-year-old Hispanic male developed hepatitis approximately 12 months after beginning therapy with chaparral capsules. Liver biopsy showed hepatocellular injury with necrosis and periportal inflammation. Liver function stabilized 6 weeks after hepatitis was first recognized.	1	0	1	1
45-year-old patient was sent to our department because of highly elevated transaminases and elevated lactate dehydrogenase. His medical history was unremarkable and he took no medication on regular basis. Physical examination did not detect any abnormalities. There was no evidence for viral hepatitis, Epstein–Barr virus or cytomegalovirus, autoimmune hepatitis, Budd–Chiari syndrome, haemochromatosis or Wilson's disease. During the interview he admitted that for ‘prophylactic reasons’ he had been drinking the juice of Noni (Morinda citrifolia), a Polynesian herbal remedy made from a tropical fruit, during the preceding 3 weeks. This gave rise to the suspicion of herbal toxicity, which was confirmed by a liver biopsy. After ceasing the ingestion of Noni, transaminase levels normalized quickly and were within normal ranges 1 month after the first presentation. To our knowledge, this is the first report of hepatotoxicity caused by this herbal remedy, which has been highly praised in the tabloid press.	1	1	1	1
Testosterone boosters are heavily marketed on social media and marketplaces to men with claims to significantly increase testosterone. Lax industry regulation has allowed sales of supplements to thrive in the absence of verification of their	0	0	0	0

purported benefits. Our primary objective was to systematically review all data published in the last two decades on testosterone boosters and determine their efficacy. Our outcome of interest was total testosterone increase versus placebo in four different populations: male athletes, men with late-onset hypogonadism infertile men and healthy men. Following search and screening, 52 studies were included in our review, relating to 27 proposed testosterone boosters: 10 studies of cholecalciferol; 5 zinc/magnesium; 4 Tribulus terrestris and creatine; 3 Eurycoma longifolia and Withania somnifera; 2 betaine, D-aspartic acid, Lepidium meyenii and isoflavones; while the remainder were single reports. Our findings indicate that most fail to increase total testosterone. The exceptions were beta-hydroxy beta-methylbutyrate and betaine, which can be considered effective for male athletes. Eurycoma longifolia, a blend of Punica granatum fruit rind and Theobroma cacao seed extracts (Tesnor()) and purified Shilajit extract (PrimaVie()) can be considered possibly effective for men with late-onset hypogonadism; Eurycoma longifolia and Withania somnifera possibly effective for healthy men; and a non-hormonal aromatase inhibitor (Novadex XT()) possibly effective for male athletes.				
