# Supplementary material for: Comprehensive safety evaluation of Withania somnifera (Ashwagandha): an AI-driven meta-analysis and quantitative structure–activity relationship based toxicity assessment
Source: Front Nutr. 2025 Nov 24;12:1658265. doi: 10.3389/fnut.2025.1658265 (PMC12682666; doi:10.3389/fnut.2025.1658265)
Supplement: Supplementary file 3 [file Data_Sheet_3.PDF]

Supplementary table 3 – Bootstrap Confidence Intervals

We compared each model with each other using bootstrap CI.

We used 10,000 bootstrapped samples, taken from our data points broken down to different groups, for each test, using 2.5th and 97.5 percentiles for an initial 95% confidence interval, if a model was significantly better than the other model, we would test 0.5th and 99.5th for 99% interval and if the model was still significantly better than the other, we would test for 0.05th and 99.95th percentile for 99.9% confidence interval.

In here there are tables that show our results for those were found to be statistically significant.

**GPT 4 (OpenAI) model accuracy and SciBERT model accuracy**		
**Confidence Interval**	**Confidence Interval Difference**	**Results**
95% confidence interval	(0.071, 0.357)	GPT 4 is significantly better
99% confidence interval	(0.029, 0.4)	GPT 4 is significantly better
99.9% confidence interval	(-1.1e-16, 0.444)	No significant difference

**GPT 4 (OpenAI) model F-Score and SciBERT model F-Score**		
**Confidence Interval**	**Confidence Interval Difference**	**Results**
95% confidence interval	(0.093, 0.31)	GPT 4 is significantly better
99% confidence interval	(0.061, 0.349)	GPT 4 is significantly better
99.9% confidence interval	(0.018, 0.375)	GPT 4 is significantly better
