# Supplementary material for: Comprehensive safety evaluation of Withania somnifera (Ashwagandha): an AI-driven meta-analysis and quantitative structure–activity relationship based toxicity assessment
Source: Front Nutr. 2025 Nov 24;12:1658265. doi: 10.3389/fnut.2025.1658265 (PMC12682666; doi:10.3389/fnut.2025.1658265)
Supplement: Supplementary file 4 [file Data_Sheet_4.PDF]

Supplementary table 4 - Withania somnifera NLP toxicity prediction

Title	Link	Type Publication	Date	Journal Title	Cytotoxicity	Liver toxicity	Thyroid toxicity
Withania somnifera (L.) Dunal (Ashwagandha): A comprehensive review on ethnopharmacology, pharmacotherapeutics, biomedicinal and toxicological aspects.	https://pubmed.ncbi.nlm.nih.gov/34649336	Journal Article, Review	2021	Biomedicine & pharmacotherapy = Biomedecine & pharmacotherapie	1	0	0
Withania somnifera Root Extract Has Potent Cytotoxic Effect against Human Malignant Melanoma Cells.	https://pubmed.ncbi.nlm.nih.gov/26334881	Journal Article, Research Support, Non-U.S. Gov't	2015	PloS one	1	0	0
Steroidal constituents isolated from the seeds of Withania somnifera.	https://pubmed.ncbi.nlm.nih.gov/31538506	Journal Article	2021	Natural product research	1	0	0
Antileishmanial and lung adenocarcinoma cell toxicity of Withania somnifera (Linn.) dunal root and fruit extracts.	https://pubmed.ncbi.nlm.nih.gov/34520289	Journal Article	2022	Natural product research	1	0	0
Comparing the cytotoxic potential of Withania somnifera water and methanol extracts.	https://pubmed.ncbi.nlm.nih.gov/20448853	Comparative Study, Journal Article, Research Support, Non-U.S. Gov't	2009	African journal of traditional, complementary, and alternative medicines : AJTCAM	1	0	0
In Vitro Anticancer Activity of the Root, Stem and Leaves of Withania Somnifera against Various Human Cancer Cell Lines.	https://pubmed.ncbi.nlm.nih.gov/21695006	Journal Article	2010	Indian journal of pharmaceutical sciences	1	0	0
Molecular authentication, metabolite profiling and in silico-in vitro cytotoxicity screening of endophytic Penicillium ramusculum from Withania somnifera for breast cancer therapeutics.	https://pubmed.ncbi.nlm.nih.gov/38344285	Journal Article	2024	3 Biotech	1	0	0
Chlorinated and diepoxy withanolides from Withania somnifera and their cytotoxic effects against human lung cancer cell line.	https://pubmed.ncbi.nlm.nih.gov/21044792	Journal Article, Research Support, Non-U.S. Gov't	2010	Phytochemistry	1	0	0
Production dynamics of Withaferin A in Withania somnifera (L.) Dunal complex.	https://pubmed.ncbi.nlm.nih.gov/19735044	Journal Article	2009	Natural product research	1	0	0
Selective reactivity of 2-mercaptoethanol with 5beta,6beta-epoxide in steroids from Withania somnifera.	https://pubmed.ncbi.nlm.nih.gov/18061225	Journal Article, Research Support, Non-U.S. Gov't	2008	Steroids	1	0	0
Withaferin A: a new radiosensitizer from the Indian medicinal plant Withania somnifera.	https://pubmed.ncbi.nlm.nih.gov/8609455	Journal Article, Research Support, Non-U.S. Gov't	1996	International journal of radiation biology	1	0	0
Cytotoxicity of plants used in traditional medicine in Yemen.	https://pubmed.ncbi.nlm.nih.gov/15890471	Journal Article	2005	Fitoterapia	1	0	0
DNA damage by Withanone as a potential cause of liver toxicity observed for herbal products of Withania somnifera (Ashwagandha).	https://pubmed.ncbi.nlm.nih.gov/34345852	Journal Article	2021	Current research in toxicology	1	1	0
A critical assessment of the whole plant-based phytotherapeutics from Withania somnifera (L.) Dunal with respect to safety and efficacy vis-a-vis leaf or root extract-based formulation.	https://pubmed.ncbi.nlm.nih.gov/37533233	Journal Article, Review	2023	Toxicology mechanisms and methods	1	0	0

Studies of medicinal plants of Sri Lanka. Part 14: Toxicity of some traditional medicinal herbs.	https://pubmed.ncbi.nlm.nih.gov/4058035	Journal Article, Research Support, Non-U.S. Gov't	1985	Journal of ethnopharmacology	1	1	0
Transcriptome analysis reveals in vitro cultured Withania somnifera leaf and root tissues as a promising source for targeted withanolide biosynthesis.	https://pubmed.ncbi.nlm.nih.gov/25608483	Journal Article, Research Support, Non-U.S. Gov't	2015	BMC genomics	1	0	0
5,6-de-epoxy-5-en-7-one-17-hydroxy withaferin A, a new cytotoxic steroid from Withania somnifera L. Dunal leaves.	https://pubmed.ncbi.nlm.nih.gov/24422976	Journal Article, Research Support, Non-U.S. Gov't	2014	Natural product research	1	0	0
Ninety-day repeated dose toxicity of Ashwagandha (Withania somnifera) root extract in Wistar rats.	https://pubmed.ncbi.nlm.nih.gov/37711361	Journal Article	2023	Toxicology reports	1	1	0
Withaferin A: A Dietary Supplement with Promising Potential as an Anti-Tumor Therapeutic for Cancer Treatment - Pharmacology and Mechanisms.	https://pubmed.ncbi.nlm.nih.gov/37753228	Journal Article, Review	2023	Drug design, development and therapy	1	0	0
Antiproliferative withanolides from several solanaceous species.	https://pubmed.ncbi.nlm.nih.gov/24871278	Journal Article, Research Support, N.I.H., Extramural, Research Support, Non-U.S. Gov't, Review	2014	Natural product research	1	0	0
Effect of Withaferin A on the development and decay of thermotolerance in B16F1 melanoma: a preliminary study.	https://pubmed.ncbi.nlm.nih.gov/19190033	Journal Article, Research Support, Non-U.S. Gov't	2009	Integrative cancer therapies	1	0	0
Anti-proliferative withanolides from the Solanaceae: a structure-activity study.	https://pubmed.ncbi.nlm.nih.gov/24098060	Journal Article	2012	Pure and applied chemistry. Chimie pure et appliquee	1	0	0
Determination of mycotoxins, alkaloids, phytochemicals, antioxidants and cytotoxicity in Asiatic ginseng (Ashwagandha, Dong quai, Panax ginseng).	https://pubmed.ncbi.nlm.nih.gov/28553006	Journal Article	2017	Chemicke zvesti	1	1	0
Withaferin A induces mitochondrial-dependent apoptosis in non-small cell lung cancer cells via generation of reactive oxygen species.	https://pubmed.ncbi.nlm.nih.gov/28365961	Journal Article	2017	Journal of B.U.ON. : official journal of the Balkan Union of Oncology	1	0	0
Potential Anti-Tumorigenic Properties of Diverse Medicinal Plants against the Majority of Common Types of Cancer.	https://pubmed.ncbi.nlm.nih.gov/38794144	Journal Article, Review	2024	Pharmaceuticals (Basel, Switzerland)	1	0	0
Withaferin a alters intermediate filament organization, cell shape and behavior.	https://pubmed.ncbi.nlm.nih.gov/22720028	Journal Article, Research Support, N.I.H., Extramural	2012	PloS one	1	0	0
Withaferin A-stimulated Ca2+ entry, ceramide formation and suicidal death of erythrocytes.	https://pubmed.ncbi.nlm.nih.gov/22989414	Journal Article, Research Support, Non-U.S. Gov't	2013	Toxicology in vitro : an international journal published in association with BIBRA	1	0	0
Withaferin A induces apoptosis by ROS-dependent mitochondrial dysfunction in human colorectal cancer cells.	https://pubmed.ncbi.nlm.nih.gov/29966656	Journal Article, Research Support, Non-U.S. Gov't	2018	Biochemical and biophysical research communications	1	0	0
Antiproliferation potential of withaferin A on human osteosarcoma cells via the inhibition of G2/M checkpoint proteins.	https://pubmed.ncbi.nlm.nih.gov/26170956	Journal Article	2015	Experimental and therapeutic medicine	1	0	0

Production and Structural Diversification of Withanolides by Aeroponic Cultivation of Plants of Solanaceae: Cytotoxic and Other Withanolides from Aeroponically Grown Physalis coztomatl.	https://pubmed.ncbi.nlm.nih.gov/35164184	Journal Article	2022	Molecules (Basel, Switzerland)	1	0	0
Painless Thyroiditis by Withania somnifera (Ashwagandha).	https://pubmed.ncbi.nlm.nih.gov/38559552	Case Reports	2024	Cureus	0	0	1
A comprehensive review on the hepatotoxicity of herbs used in the Indian (Ayush) systems of alternative medicine.	https://pubmed.ncbi.nlm.nih.gov/38640296	Journal Article, Review	2024	Medicine	0	1	0
MYCOLOGICAL ANALYSIS AND AFLATOXIN B(1) CONTAMINANT ESTIMATION OF HERBAL DRUG RAW MATERIALS.	https://pubmed.ncbi.nlm.nih.gov/28487902	Journal Article	2016	African journal of traditional, complementary, and alternative medicines : AJTCAM	0	1	0
Heart Toxicity Related to Herbs and Dietary Supplements: Online Table of Case Reports. Part 4 of 5.	https://pubmed.ncbi.nlm.nih.gov/28981338	Case Reports, Journal Article, Review	2018	Journal of dietary supplements	0	1	0
Herb-Induced Liver Injury by Ayurvedic Ashwagandha as Assessed for Causality by the Updated RUCAM: An Emerging Cause.	https://pubmed.ncbi.nlm.nih.gov/37631044	Case Reports	2023	Pharmaceuticals (Basel, Switzerland)	0	1	0
Ashwagandha-induced liver injury: A case series from Iceland and the US Drug-Induced Liver Injury Network.	https://pubmed.ncbi.nlm.nih.gov/31991029	Journal Article, Research Support, N.I.H., Extramural, Research Support, N.I.H., Intramural	2020	Liver international : official journal of the International Association for the Study of the Liver	0	1	0
