# Supplementary material for: Comprehensive safety evaluation of Withania somnifera (Ashwagandha): an AI-driven meta-analysis and quantitative structure–activity relationship based toxicity assessment
Source: Front Nutr. 2025 Nov 24;12:1658265. doi: 10.3389/fnut.2025.1658265 (PMC12682666; doi:10.3389/fnut.2025.1658265)
Supplement: Supplementary file 5 [file Data_Sheet_5.PDF]

Supplementary table 5 - Molecular Structure Prediction for *Withania somnifera* Molecules

PubChem ID	IUPAC Name	Common Name	Chemical Class	Root		Liver Toxicity		Reproductive Toxicity	
				Yes/No	Confidence	Yes/No	Confidence	Yes/No	Confidence
**CID 6385802**	2-amino-4-(hydroxyphosphonoyl)butanoic acid	Phosphinoalanine	Other	No	0.09	No	0.47	No	0.40
**CID 53921228**	tricos-10-enoic acid	tricos-10-enoic acid	Other	No	0.07	No	0.33	No	0.32
**CID 72809456**	6-[1-hydroxy-1-(9-hydroxy-9a,11a-dimethyl-7-{[3,4,5-trihydroxy-6-({[3,4,5-trihydroxy-6-(hydroxymethyl)oxan-2-yl]oxy}methyl)oxan-2-yl]oxy}-1H,2H,3H,3aH,3bH,4H,6H,7H,8H,9H,9aH,9bH,10H,11H,11aH-cyclopenta[a]phenanthren-1-yl)ethyl]-3,4-dimethyloxan-2-one		Withanolide glycosides and derivatives	No	0.53	No	0.01	No	0.05
**CID 53436342**	icos-8-enoic acid	icos-8-enoic acid	Other	No	0.00	No	0.33	No	0.32
**CID 71402235**	nonadec-11-enoic acid	nonadec-11-enoic acid	Long-chain fatty acids	No	0.00	No	0.33	No	0.32
**CID 65032**	tetracos-15-enoic acid	Nervonsaeure	Very long-chain fatty acids	No	0.07	No	0.33	No	0.32
**CID 78173488**	6-hydroxy-2,16-dimethyl-15-{1-[4-methyl-6-oxo-5-({[3,4,5-trihydroxy-6-(hydroxymethyl)oxan-2-yl]oxy}methyl)-3,6-dihydro-2H-pyran-2-yl]ethyl}-8-oxapentacyclo[9.7.0.$0^{2,7}$.$0^{7,9}$.$0^{12,16}$]octadec-4-en-3-one	Sitoindoside Ix	Withanolides and derivatives	Yes	0.80	No	0.02	No	0.02
**CID 175998**	2-(hydroxymethyl)-6-sulfanyloxane-3,4,5-triol	2-(hydroxymethyl)-6-sulfanyloxane-3,4,5-triol	Hexoses	No	0.45	No	0.31	No	0.42
**CID 163088969**	5,6-dihydroxy-2,16-dimethyl-15-{1-[4-methyl-6-oxo-5-({[3,4,5-trihydroxy-6-(hydroxymethyl)oxan-2-yl]oxy}methyl)-3,6-dihydro-2H-pyran-2-yl]ethyl}-8-oxapentacyclo[9.7.0.$0^{2,7}$.$0^{7,9}$.$0^{12,16}$]octadecan-3-one		Withanolides and derivatives	Yes	0.70	No	0.02	No	0.01
**CID 163025782**	6-[1-(5,9-dihydroxy-10,14-dimethyl-7-{[3,4,5-trihydroxy-6-({[3,4,5-trihydroxy-6-(hydroxymethyl)oxan-2-yl]oxy}methyl)oxan-2-yl]oxy}-3-oxapentacyclo[9.7.0.$0^{2,4}$.$0^{5,10}$.$0^{14,18}$]octadecan-15-yl)ethyl]-3,4-dimethyl-5,6-dihydro-2H-pyran-2-one	Withanoside Ii	Withanolide glycosides and derivatives	No	0.55	No	0.02	No	0.01
**CID 71318435**	heptadec-11-enoic acid	heptadec-11-enoic acid	Long-chain fatty acids	No	0.05	No	0.33	No	0.32
**CID 163045604**	6-{1-hydroxy-1-[4-hydroxy-11a-(hydroxymethyl)-9a-methyl-9-oxo-1H,2H,3H,3aH,3bH,4H,5H,9H,9aH,9bH,10H,11H,11aH-cyclopenta[a]phenanthren-1-yl]ethyl}-3-(hydroxymethyl)-4-methyl-5,6-dihydro-2H-pyran-2-one		Withanolides and derivatives	Yes	0.83	No	0.02	No	0.04
**CID 310015**	6-(1-{1,5,5a-trihydroxy-9a,11a-dimethyl-9-oxo-1H,2H,3H,3aH,3bH,4H,5H,5aH,6H,9H,9aH,9bH,10H,11H,11aH-cyclopenta[a]phenanthren-1-yl}-1-hydroxyethyl)-3,4-dimethyl-5,6-dihydro-2H-pyran-2-one		Withanolides and derivatives	Yes	0.79	No	0.02	No	0.03
**CID 53440328**	docos-15-enoic acid	docos-15-enoic acid	Very long-chain fatty acids	No	0.03	No	0.33	No	0.32
**CID 73836090**	octacos-11-enoic acid	octacos-11-enoic acid	Other	No	0.18	No	0.33	No	0.32
**CID 54010727**	icosa-7,10,13-trienoic acid	icosa-7,10,13-trienoic acid	Long-chain fatty acids	No	0.00	No	0.24	No	0.23
**CID 38626**	nonahexacontanoic acid	nonahexacontanoic acid	Very long-chain fatty acids	No	0.16	No	0.51	No	0.43

**CID 122544**	1-(5-ethyl-6-methylhept-3-en-2-yl)-9a,11a-dimethyl-1H,2H,3H,3aH,3bH,4H,6H,7H,8H,9H,9aH,9bH,10H,11H,11aH-cyclopenta[a]phenanthren-7-ol	Fucostanol	Stigmastanes and derivatives	No	0.38	No	0.10	No	0.11
**CID 3808690**	6-(1-{5,5a-dihydroxy-9a,11a-dimethyl-9-oxo-1H,2H,3H,3aH,3bH,4H,5H,5aH,6H,9H,9aH,9bH,10H,11H,11aH-cyclopenta[a]phenanthren-1-yl}ethyl)-3-(hydroxymethyl)-4-methyl-5,6-dihydro-2H-pyran-2-one		Withanolides and derivatives	Yes	0.82	No	0.03	No	0.02
**CID 78382117**	octacos-19-enoic acid	octacos-19-enoic acid	Very long-chain fatty acids	No	0.18	No	0.33	No	0.32
**CID 5282596**	hexatriacontanoic acid	hexatriacontanoic acid	Very long-chain fatty acids	No	0.20	No	0.51	No	0.43
**CID 78382121**	hexacosa-17,20-dienoic acid	hexacosa-17,20-dienoic acid	Very long-chain fatty acids	No	0.17	No	0.24	No	0.23
**CID 71359760**	docosa-7,15-dienoic acid	docosa-7,15-dienoic acid	Other	No	0.03	No	0.33	No	0.32
**CID 54090186**	docos-14-enoic acid	docos-14-enoic acid	Very long-chain fatty acids	No	0.03	No	0.33	No	0.32
**CID 10468**	pentacosanoic acid	pentacosanoic acid	Very long-chain fatty acids	No	0.12	No	0.51	No	0.43
**CID 301758**	15-[1-(4,5-dimethyl-6-oxo-3,6-dihydro-2H-pyran-2-yl)ethyl]-6-hydroxy-2,16-dimethyl-8-oxapentacyclo $[9.7.0.0^{2,7}.0^{7,9}.0^{12,16}]$octadec-4-en-3-one		Withanolides and derivatives	Yes	0.78	No	0.03	No	0.09
**CID 72731335**	icos-12-enoic acid	icos-12-enoic acid	Other	No	0.00	No	0.33	No	0.32
**CID 162869943**	hexacos-14-enoic acid	hexacos-14-enoic acid	Very long-chain fatty acids	No	0.14	No	0.33	No	0.32
**CID 163005928**	6-[1-(5,9-dihydroxy-10,14-dimethyl-7-{[3,4,5-trihydroxy-6-({[3,4,5-trihydroxy-6-(hydroxymethyl)oxan-2-yl]oxy}methyl)oxan-2-yl]oxy}-3-oxapentacyclo $[9.7.0.0^{2,4}.0^{5,10}.0^{14,18}]$octadecan-15-yl)ethyl]-3-(hydroxymethyl)-4-methyl-5,6-dihydro-2H-pyran-2-one		Withanolide glycosides and derivatives	No	0.57	No	0.03	No	0.03
**CID 522644**	dohexacontane	dohexacontane	Alkanes	No	0.48	No	0.57	No	0.58
**CID 73025149**	3-(hydroxymethyl)-4-methyl-6-[1-(3,6,14-trihydroxy-2,16-dimethyl-5-{[3,4,5-trihydroxy-6-(hydroxymethyl)oxan-2-yl]oxy}-8-oxapentacyclo$[9.7.0.0^{2,7}.0^{7,9}.0^{12,16}]$octadecan-15-yl)ethyl]-5,6-dihydro-2H-pyran-2-one		Withanolide glycosides and derivatives	No	0.62	No	0.03	No	0.02
**CID 3011**	icosa-8,11,14-trienoic acid	Dgla	Long-chain fatty acids	No	0.00	No	0.24	No	0.23
**CID 16196976**	6-[1-(9-hydroxy-9a,11a-dimethyl-7-{[3,4,5-trihydroxy-6-({[3,4,5-trihydroxy-6-(hydroxymethyl)oxan-2-yl]oxy}methyl)oxan-2-yl]oxy}-1H,2H,3H,3aH,3bH,4H,6H,7H,8H,9H,9aH,9bH,10H,11H,11aH-cyclopenta[a]phenanthren-1-yl)ethyl]-3,4-dimethyl-5,6-dihydro-2H-pyran-2-one	Withanoside V	Withanolide glycosides and derivatives	No	0.55	No	0.03	No	0.04
**CID 24318**	hexacontane	hexacontane	Alkanes	No	0.48	No	0.57	No	0.58
**CID 54146268**	docosa-7,10,13-trienoic acid	docosa-7,10,13-trienoic acid	Very long-chain fatty acids	No	0.02	No	0.24	No	0.23

**CID 162984193**	6-[1-(9-hydroxy-9a,11a-dimethyl-7-{[3,4,5-trihydroxy-6-({[3,4,5-trihydroxy-6-(hydroxymethyl)oxan-2-yl]oxy}methyl)oxan-2-yl]oxy}-1H,2H,3H,3aH,3bH,4H,6H,7H,8H,9H,9aH,9bH,10H,11H,11aH-cyclopenta[a]phenanthren-1-yl)ethyl]-4-methyl-3-({[3,4,5-trihydroxy-6-(hydroxymethyl)oxan-2-yl]oxy}methyl)-5,6-dihydro-2H-pyran-2-one		Withanolide glycosides and derivatives	No	0.52	No	0.03	No	0.03
**CID 85733700**	tricosa-14,17-dienoic acid	tricosa-14,17-dienoic acid	Very long-chain fatty acids	No	0.09	No	0.24	No	0.23
**CID 54149323**	tetracos-17-enoic acid	tetracos-17-enoic acid	Very long-chain fatty acids	No	0.07	No	0.33	No	0.32
**CID 37982**	hentriacontanoic acid	hentriacontanoic acid	Very long-chain fatty acids	No	0.20	No	0.51	No	0.43
**CID 151512**	octadec-12-enoic acid	octadec-12-enoic acid	Long-chain fatty acids	No	0.05	No	0.33	No	0.32
**CID 57353034**	triaconta-15,18,21,24-tetraenoic acid	triaconta-15,18,21,24-tetraenoic acid	Other	No	0.17	No	0.24	No	0.23
**CID 81067**	pentacontane	pentacontane	Alkanes	No	0.50	No	0.57	No	0.58
**CID 543268**	hexadec-7-enoic acid	hexadec-7-enoic acid	Long-chain fatty acids	No	0.07	No	0.33	No	0.32
**CID 162897404**	6-(1-{3,6-dihydroxy-2,16-dimethyl-8-oxapentacyclo $[9.7.0.0^{2,7}.0^{7,9}.0^{12,16}]$octadec-4-en-15-yl}ethyl)-3-(hydroxymethyl)-4-methyl-5,6-dihydro-2H-pyran-2-one		Withanolides and derivatives	Yes	0.84	No	0.03	No	0.05
**CID 71341107**	tetracos-11-enoic acid	tetracos-11-enoic acid	Other	No	0.07	No	0.33	No	0.32
**CID 72977891**	6-[1-(9-hydroxy-9a,11a-dimethyl-7-{[3,4,5-trihydroxy-6-(hydroxymethyl)oxan-2-yl]oxy}-1H,2H,3H,3aH,3bH,4H,6H,7H,8H,9H,9aH,9bH,10H,11H,11aH-cyclopenta[a]phenanthren-1-yl)ethyl]-4-methyl-3-({[3,4,5-trihydroxy-6-(hydroxymethyl)oxan-2-yl]oxy}methyl)-5,6-dihydro-2H-pyran-2-one	Withanoside X	Withanolide glycosides and derivatives	No	0.55	No	0.03	No	0.04
**CID 163077004**	6-[1-(4,9-dihydroxy-9a,11a-dimethyl-7-{[3,4,5-trihydroxy-6-({[3,4,5-trihydroxy-6-(hydroxymethyl)oxan-2-yl]oxy}methyl)oxan-2-yl]oxy}-1H,2H,3H,3aH,3bH,4H,6H,7H,8H,9H,9aH,9bH,10H,11H,11aH-cyclopenta[a]phenanthren-1-yl)ethyl]-3,4-dimethyl-5,6-dihydro-2H-pyran-2-one		Withanolide glycosides and derivatives	No	0.55	No	0.03	No	0.03
**CID 73721022**	pentacos-18-enoic acid	pentacos-18-enoic acid	Very long-chain fatty acids	No	0.11	No	0.33	No	0.32
**CID 71343178**	pentacos-16-enoic acid	pentacos-16-enoic acid	Very long-chain fatty acids	No	0.11	No	0.33	No	0.32
**CID 162853685**	triacont-11-enoic acid	triacont-11-enoic acid	Very long-chain fatty acids	No	0.20	No	0.33	No	0.32
**CID 12745**	octadec-10-enoic acid	octadec-10-enoic acid	Long-chain fatty acids	No	0.05	No	0.33	No	0.32
**CID 54252776**	triacont-21-enoic acid	triacont-21-enoic acid	Very long-chain fatty acids	No	0.20	No	0.33	No	0.32
**CID 162800954**	triaconta-18,21,24-trienoic acid	triaconta-18,21,24-trienoic acid	Other	No	0.19	No	0.24	No	0.23
**CID 85776436**	henicos-9-enoic acid	henicos-9-enoic acid	Long-chain fatty acids	No	0.03	No	0.33	No	0.32
**CID 54268499**	docosa-10,13,16-trienoic acid	docosa-10,13,16-trienoic acid	Very long-chain fatty acids	No	0.02	No	0.24	No	0.23

**CID 141738142**	tetratriaconta-20,23-dienoic acid	tetratriaconta-20,23-dienoic acid	Very long-chain fatty acids	No	0.26	No	0.24	No	0.23
**CID 53394634**	icos-14-enoic acid	icos-14-enoic acid	Long-chain fatty acids	No	0.00	No	0.33	No	0.32
**CID 54280271**	hexacos-17-enoic acid	hexacos-17-enoic acid	Very long-chain fatty acids	No	0.14	No	0.33	No	0.32
**CID 85344208**	6-[1-(9-hydroxy-9a,11a-dimethyl-7-{[3,4,5-trihydroxy-6-({[3,4,5-trihydroxy-6-(hydroxymethyl)oxan-2-yl]oxy}methyl)oxan-2-yl]oxy}-1H,2H,3H,3aH,3bH,4H,6H,7H,8H,9H,9aH,9bH,10H,11H,11aH-cyclopenta[a]phenanthren-1-yl)ethyl]-4-methyl-3-{[(3,4,5,6-tetrahydroxyoxan-2-yl)methoxy]methyl}-5,6-dihydro-2H-pyran-2-one	Withanoside Viii	Other	No	0.52	No	0.03	No	0.05
**CID 72732074**	icos-10-enoic acid	icos-10-enoic acid	Long-chain fatty acids	No	0.00	No	0.33	No	0.32
**CID 20245**	nonacosanoic acid	Nonacosylic Acid	Very long-chain fatty acids	No	0.20	No	0.51	No	0.43
**CID 163022240**	6-[1-(9-hydroxy-9a,11a-dimethyl-7-{[3,4,5-trihydroxy-6-(hydroxymethyl)oxan-2-yl]oxy}-1H,2H,3H,3aH,3bH,4H,6H,7H,8H,9H,9aH,9bH,10H,11H,11aH-cyclopenta[a]phenanthren-1-yl)ethyl]-3,4-dimethyl-5,6-dihydro-2H-pyran-2-one		Withanolide glycosides and derivatives	No	0.69	No	0.03	No	0.09
**CID 12388**	tridecane	Tridekan	Alkanes	No	0.18	No	0.57	No	0.58
**CID 5293655**	2-(3,4-dihydroxyphenyl)-5,7-dihydroxy-3-[(3,4,5-trihydroxy-6-{[(3,4,5-trihydroxy-6-methyloxan-2-yl)oxy]methyl}oxan-2-yl)oxy]-4H-chromen-4-one	Rutin	Flavonoid-3-O-glycosides	No	0.31	No	0.03	No	0.06
**CID 85163882**	15-[1-(4,5-dimethyl-6-oxooxan-2-yl)-1-hydroxyethyl]-6-hydroxy-5-methoxy-2,16-dimethyl-8-oxapentacyclo $[9.7.0.0^{2,7}.0^{7,9}.0^{12,16}]$octadecan-3-one		Withanolides and derivatives	Yes	0.74	No	0.03	No	0.08
**CID 8900**	heptane	heptane	Alkanes	No	0.31	No	0.57	No	0.58
**CID 73668170**	octacos-10-enoic acid	octacos-10-enoic acid	Very long-chain fatty acids	No	0.18	No	0.33	No	0.32
**CID 94485**	tetratriacontanoic acid	tetratriacontanoic acid	Very long-chain fatty acids	No	0.20	No	0.51	No	0.43
**CID 985**	hexadecanoic acid	Palmitate	Long-chain fatty acids	No	0.08	No	0.51	No	0.43
**CID 54380165**	nonadec-12-enoic acid	nonadec-12-enoic acid	Long-chain fatty acids	No	0.00	No	0.33	No	0.32
**CID 71343354**	tricos-15-enoic acid	tricos-15-enoic acid	Very long-chain fatty acids	No	0.07	No	0.33	No	0.32
**CID 10471**	triacontanoic acid	triacontanoic acid	Very long-chain fatty acids	No	0.20	No	0.51	No	0.43
**CID 45359708**	6-[1-hydroxy-1-(9-hydroxy-9a,11a-dimethyl-7-{[3,4,5-trihydroxy-6-({[3,4,5-trihydroxy-6-(hydroxymethyl)oxan-2-yl]oxy}methyl)oxan-2-yl]oxy}-1H,2H,3H,3aH,3bH,4H,6H,7H,8H,9H,9aH,9bH,10H,11H,11aH-cyclopenta[a]phenanthren-1-yl)ethyl]-3,4-dimethyl-5,6-dihydro-2H-pyran-2-one	Withanoside Vi	Withanolide glycosides and derivatives	No	0.55	No	0.03	No	0.04
**CID 12591**	nonadecanoic acid	Nonadecylic Acid	Long-chain fatty acids	No	0.01	No	0.51	No	0.43
**CID 53748576**	henicos-12-enoic acid	henicos-12-enoic acid	Long-chain fatty acids	No	0.03	No	0.33	No	0.32
**CID 23524**	heptacosanoic acid	heptacosanoic acid	Very long-chain fatty acids	No	0.18	No	0.51	No	0.43

**CID 16614**	octadeca-8,11-dienoic acid	octadeca-8,11-dienoic acid	Lineolic acids and derivatives	No	0.07	No	0.24	No	0.23
**CID 71406001**	hexacos-9-enoic acid	hexacos-9-enoic acid	Very long-chain fatty acids	No	0.14	No	0.33	No	0.32
**CID 348159**	3-{[3-(3,4-dihydroxyphenyl)prop-2-enoyl]oxy}-1,4,5-trihydroxycyclohexane-1-carboxylic acid	Heriguard	Quinic acids and derivatives	No	0.63	No	0.03	No	0.04
**CID 73721020**	tricos-16-enoic acid	tricos-16-enoic acid	Very long-chain fatty acids	No	0.07	No	0.33	No	0.32
**CID 129853947**	nonacos-22-enoic acid		Other	No	0.20	No	0.51	No	0.18
**CID 33604**	octadec-8-enoic acid	octadec-8-enoic acid	Long-chain fatty acids	No	0.05	No	0.33	No	0.32
**CID 78382126**	docosa-8,11,14-trienoic acid	docosa-8,11,14-trienoic acid	Very long-chain fatty acids	No	0.02	No	0.24	No	0.23
**CID 73306656**	15-[1-(4,5-dimethyl-6-oxo-3,6-dihydro-2H-pyran-2-yl) ethyl]-6,15-dihydroxy-2,16-dimethyl-8-oxapentacyclo [9.7.0.0^{2,7}.0^{7,9}.0^{12,16}]octadec-4-en-3-one	Tubocapsanolide F	Other	Yes	0.85	No	0.03	No	0.07
**CID 57294532**	docos-12-enoic acid	docos-12-enoic acid	Very long-chain fatty acids	No	0.03	No	0.33	No	0.32
**CID 151065**	icosa-8,11-dienoic acid	icosa-8,11-dienoic acid	Long-chain fatty acids	No	0.02	No	0.24	No	0.23
**CID 71331992**	docosa-10,13-dienoic acid	docosa-10,13-dienoic acid	Very long-chain fatty acids	No	0.09	No	0.24	No	0.23
**CID 71345359**	hexacosa-9,19-dienoic acid	hexacosa-9,19-dienoic acid	Other	No	0.17	No	0.33	No	0.32
**CID 54518967**	nonadec-9-enoic acid	nonadec-9-enoic acid	Long-chain fatty acids	No	0.00	No	0.33	No	0.32
**CID 92987**	1-(piperidin-2-yl)propan-2-one	Pelletierine	Piperidines	No	0.42	No	0.57	No	0.57
**CID 54530337**	icosa-7,13-dienoic acid		Other	No	0.02	No	0.41	No	0.11
**CID 71442740**	hexacos-19-enoic acid	hexacos-19-enoic acid	Other	No	0.14	No	0.33	No	0.32
**CID 54538289**	heptadec-8-enoic acid	heptadec-8-enoic acid	Long-chain fatty acids	No	0.05	No	0.33	No	0.32
**CID 72999858**	6-(1-{7,9-dihydroxy-9a,11a-dimethyl-1H,2H,3H,3aH,3bH, 4H,6H,7H,8H,9H,9aH,9bH,10H,11H,11aH-cyclopenta[a] phenanthren-1-yl}ethyl)-3-(hydroxymethyl)-4-methyl-5,6-dihydro-2H-pyran-2-one		Withanolides and derivatives	No	0.68	No	0.03	No	0.09
**CID 225689**	4,4,6a,6b,8a,11,11,14b-octamethyl-1,2,3,4,4a,5,6,6a,6b, 7,8,8a,9,10,11,12,12a,14,14a,14b-icosahydropicen-3-ol	Amyrin	Triterpenoids	No	0.37	No	0.04	No	0.04
**CID 74039188**	6-hydroxy-15-{1-hydroxy-1-[5-(hydroxymethyl)-4-methyl-6-oxo-3,6-dihydro-2H-pyran-2-yl]ethyl}-2,16-dimethyl-8-oxapentacyclo[9.7.0.0^{2,7}.0^{7,9}.0^{12,16}]octadec-4-en-3-one	5,6-Epoxy-4,20,27-Trihydroxy-1-Oxowitha-2,24-Dienolide	Withanolides and derivatives	Yes	0.93	No	0.03	No	0.07
**CID 71446842**	heptadeca-8,11-dienoic acid	heptadeca-8,11-dienoic acid	Long-chain fatty acids	No	0.05	No	0.24	No	0.23
**CID 72787720**	15-[1-(4,5-dimethyl-6-oxo-3,6-dihydro-2H-pyran-2-yl)-1-hydroxyethyl]-5,7-dihydroxy-10,14-dimethyl-3-oxapentacyclo[9.7.0.0^{2,4}.0^{5,10}.0^{14,18}]octadecan-9-one		Withanolides and derivatives	Yes	0.90	No	0.03	No	0.06
**CID 301754**	15-[1-(4,5-dimethyl-6-oxo-3,6-dihydro-2H-pyran-2-yl)-1-hydroxyethyl]-6-hydroxy-2,16-dimethyl-8-oxapentacyclo [9.7.0.0^{2,7}.0^{7,9}.0^{12,16}]octadec-4-en-3-one	Withaferin D	Withanolides and derivatives	Yes	0.87	No	0.03	No	0.08

**CID 162888923**	6-[1-(5,9-dihydroxy-10,14-dimethyl-7-{[3,4,5-trihydroxy-6-(hydroxymethyl)oxan-2-yl]oxy}-3-oxapentacyclo [9.7.0.0^{2,4}.0^{5,10}.0^{14,18}]octadecan-15-yl)ethyl]-3-(hydroxymethyl)-4-methyl-5,6-dihydro-2H-pyran-2-one	Withanoside Iii	Withanolide glycosides and derivatives	No	0.66	No	0.03	No	0.04
**CID 162986859**	5-hydroxy-15-{1-hydroxy-1-[5-(hydroxymethyl)-4-methyl-6-oxo-3,6-dihydro-2H-pyran-2-yl]ethyl}-10,14-dimethyl-3-oxapentacyclo[9.7.0.0^{2,4}.0^{5,10}.0^{14,18}]octadec-7-en-9-one		Withanolides and derivatives	Yes	0.93	No	0.04	No	0.05
**CID 14605184**	15-[1-(4,5-dimethyl-6-oxooxan-2-yl)-1-hydroxyethyl]-5-hydroxy-10,14-dimethyl-3-oxapentacyclo [9.7.0.0^{2,4}.0^{5,10}.0^{14,18}]octadec-7-en-9-one		Withanolides and derivatives	Yes	0.83	No	0.04	No	0.03
**CID 163009498**	6-(1-{6-hydroxy-9a,11a-dimethyl-9-oxo-1H,2H,3H,3aH,3bH,4H,6H,9H,9aH,9bH,10H,11H,11aH-cyclopenta[a]phenanthren-1-yl}ethyl)-3-(hydroxymethyl)-4-methyl-5,6-dihydro-2H-pyran-2-one		Withanolides and derivatives	No	0.67	No	0.04	No	0.07
**CID 162893110**	11-[1-(4,5-dimethyl-6-oxo-3,6-dihydro-2H-pyran-2-yl)-1-hydroxyethyl]-18-hydroxy-6,10-dimethyl-17-oxapentacyclo[14.1.1.0^{1,6}.0^{7,15}.0^{10,14}]octadec-3-en-5-one		Withanolides and derivatives	Yes	0.87	No	0.04	No	0.01
**CID 163041831**	6-(1-{5,5a,6-trihydroxy-9a,11a-dimethyl-9-oxo-hexadecahydro-1H-cyclopenta[a]phenanthren-1-yl}ethyl)-3-(hydroxymethyl)-4-methyl-5,6-dihydro-2H-pyran-2-one		Withanolides and derivatives	Yes	0.85	No	0.04	No	0.05
**CID 73093474**	6-[1-(9-hydroxy-9a,11a-dimethyl-7-{[3,4,5-trihydroxy-6-(hydroxymethyl)oxan-2-yl]oxy}-1H,2H,3H,3aH,3bH,4H,6H,7H,8H,9H,9aH,9bH,10H,11H,11aH-cyclopenta[a]phenanthren-1-yl)ethyl]-3-(hydroxymethyl)-4-methyl-5,6-dihydro-2H-pyran-2-one	Physagulin D	Withanolide glycosides and derivatives	Yes	0.70	No	0.04	No	0.08
**CID 72982317**	6-[1-(9-hydroxy-9a,11a-dimethyl-7-{[3,4,5-trihydroxy-6-({[3,4,5-trihydroxy-6-(hydroxymethyl)oxan-2-yl]oxy}methyl)oxan-2-yl]oxy}-1H,2H,3H,3aH,3bH,4H,6H,7H,8H,9H,9aH,9bH,10H,11H,11aH-cyclopenta[a]phenanthren-1-yl)ethyl]-3-(hydroxymethyl)-4-methyl-5,6-dihydro-2H-pyran-2-one	Withanoside Iv	Withanolide glycosides and derivatives	No	0.57	No	0.04	No	0.04
**CID 10465**	heptadecanoic acid	Margaric Acid	Long-chain fatty acids	No	0.04	No	0.51	No	0.43
**CID 160494**	docos-11-enoic acid	Cetoleic Acid	Very long-chain fatty acids	No	0.03	No	0.33	No	0.32
**CID 5385553**	5-hydroxy-2-(4-hydroxyphenyl)-7-{[3,4,5-trihydroxy-6-(hydroxymethyl)oxan-2-yl]oxy}-4H-chromen-4-one	Cosmetin	Flavonoid-7-O-glycosides	No	0.43	No	0.04	No	0.05
**CID 72960452**	6-[1-(7-{[6-({[3,4-dihydroxy-6-(hydroxymethyl)-5-{[3,4,5-trihydroxy-6-(hydroxymethyl)oxan-2-yl]oxy}oxan-2-yl]oxy}methyl)-3,4,5-trihydroxyoxan-2-yl]oxy}-9-hydroxy-9a,11a-dimethyl-1H,2H,3H,3aH,3bH,4H,6H,7H,8H,9H,9aH,9bH,10H,11H,11aH-cyclopenta[a]phenanthren-1-yl)ethyl]-3-(hydroxymethyl)-4-methyl-5,6-dihydro-2H-pyran-2-one		Withanolide glycosides and derivatives	No	0.54	No	0.04	No	0.04
**CID 85435491**	6,15-dihydroxy-15-{1-[5-(hydroxymethyl)-4-methyl-6-oxo-3,6-dihydro-2H-pyran-2-yl]ethyl}-2,16-dimethyl-8-oxapentacyclo[9.7.0.0^{2,7}.0^{7,9}.0^{12,16}]octadec-4-en-3-one		Withanolides and derivatives	Yes	0.93	No	0.04	No	0.08

**CID 85343763**	6-[1-hydroxy-1-(9-hydroxy-9a,11a-dimethyl-7-{[3,4,5-trihydroxy-6-(hydroxymethyl)oxan-2-yl]oxy}-1H,2H,3H,3aH,3bH,4H,6H,7H,8H,9H,9aH,9bH,10H,11H,11aH-cyclopenta[a]phenanthren-1-yl)ethyl]-3-(hydroxymethyl)-4-methyl-5,6-dihydro-2H-pyran-2-one	Withanoside Xi	Withanolide glycosides and derivatives	No	0.65	No	0.04	No	0.08
**CID 73072970**	2-{[1-(5-ethyl-6-methylhept-3-en-2-yl)-9a,11a-dimethyl-1H,2H,3H,3aH,3bH,4H,6H,7H,8H,9H,9aH,9bH,10H,11H,11aH-cyclopenta[a]phenanthren-7-yl]oxy}-6-(hydroxymethyl)oxane-3,4,5-triol	stigmasteryl 3-beta-D-glucoside	Stigmastanes and derivatives	No	0.52	No	0.04	No	0.07
**CID 77911018**	6-(1-{5,5a,6-trihydroxy-9a,11a-dimethyl-9-oxo-1H,2H,3H,3aH,3bH,4H,5H,5aH,6H,9H,9aH,9bH,10H,11H,11aH-cyclopenta[a]phenanthren-1-yl}ethyl)-3-(hydroxymethyl)-4-methyl-5,6-dihydro-2H-pyran-2-one		Withanolides and derivatives	Yes	0.85	No	0.04	No	0.04
**CID 3802778**	3,4-bis({[3-(3,4-dihydroxyphenyl)prop-2-enoyl]oxy})-1,5-dihydroxycyclohexane-1-carboxylic acid		Quinic acids and derivatives	No	0.57	No	0.04	No	0.06
**CID 250154**	nonadec-10-enoic acid	10-Nonadecenoic Acid	Long-chain fatty acids	No	0.00	No	0.33	No	0.32
**CID 74039190**	15-[1-(4,5-dimethyl-6-oxooxan-2-yl)-1-hydroxyethyl]-6-hydroxy-2,16-dimethyl-8-oxapentacyclo[9.7.0.0²,⁷.0⁷,⁹.0¹²,¹⁶]octadec-4-en-3-one		Withanolides and derivatives	Yes	0.83	No	0.04	No	0.10
**CID 12401**	nonadecane	Nonadekan	Alkanes	No	0.40	No	0.57	No	0.58
**CID 94159**	icos-9-enoic acid	Gadoleic Acid	Long-chain fatty acids	No	0.00	No	0.33	No	0.32
**CID 12840593**	6-[1-hydroxy-1-(9-hydroxy-9a,11a-dimethyl-7-{[3,4,5-trihydroxy-6-(hydroxymethyl)oxan-2-yl]oxy}-1H,2H,3H,3aH,3bH,4H,6H,7H,8H,9H,9aH,9bH,10H,11H,11aH-cyclopenta[a]phenanthren-1-yl)ethyl]-3,4-dimethyl-5,6-dihydro-2H-pyran-2-one		Withanolide glycosides and derivatives	No	0.65	No	0.04	No	0.11
**CID 53398767**	5-hydroxy-15-{1-[5-(hydroxymethyl)-4-methyl-6-oxo-3,6-dihydro-2H-pyran-2-yl]ethyl}-10,14-dimethyl-3-oxapentacyclo[9.7.0.0²,⁴.0⁵,¹⁰.0¹⁴,¹⁸]octadec-7-en-9-one		Withanolides and derivatives	Yes	0.90	No	0.04	No	0.04
**CID 14236712**	15-[1-(4,5-dimethyl-6-oxo-3,6-dihydro-2H-pyran-2-yl)ethyl]-5-hydroxy-10,14-dimethyl-3-oxapentacyclo[9.7.0.0²,⁴.0⁵,¹⁰.0¹⁴,¹⁸]octadec-7-en-9-one		Withanolides and derivatives	Yes	0.75	No	0.04	No	0.03
**CID 619166**	10-hydroxy-2,2,6a,6b,9,9,12a-heptamethyl-1,2,3,4,4a,5,6,6a,6b,7,8,8a,9,10,11,12,12a,12b,13,14b-icosahydropicene-4a-carboxylic acid	Caryophyllin	Triterpenoids	No	0.46	No	0.05	No	0.05
**CID 163010458**	15-[1-(4,5-dimethyl-6-oxo-3,6-dihydro-2H-pyran-2-yl)-1-hydroxyethyl]-6-hydroxy-2,16-dimethyl-8-oxapentacyclo[9.7.0.0²,⁷.0⁷,⁹.0¹²,¹⁶]octadecan-3-one		Withanolides and derivatives	Yes	0.87	No	0.04	No	0.04
**CID 14236708**	6-(1-{5a-hydroxy-9a,11a-dimethyl-9-oxo-1H,2H,3H,3aH,3bH,5aH,6H,9H,9aH,9bH,10H,11H,11aH-cyclopenta[a]phenanthren-1-yl}-1-hydroxyethyl)-3,4-dimethyl-5,6-dihydro-2H-pyran-2-one	Withacoagin	Withanolides and derivatives	Yes	0.74	No	0.04	No	0.00
**CID 418033**	6-hydroxy-15-{1-[5-(hydroxymethyl)-4-methyl-6-oxo-3,6-dihydro-2H-pyran-2-yl]ethyl}-2,16-dimethyl-8-oxapentacyclo[9.7.0.0²,⁷.0⁷,⁹.0¹²,¹⁶]octadecan-3-one	Dihydrowithaferin A	Withanolides and derivatives	Yes	0.81	No	0.05	No	0.06
**CID 72951313**	17-hydroxy-N-[2-(5-{[3,4,5-trihydroxy-6-({[3,4,5-trihydroxy-6-(hydroxymethyl)oxan-2-yl]oxy}methyl)oxan-2-yl]oxy}-1H-indol-3-yl)ethyl]octadeca-6,9,12,15-tetraenamide	Withanamide H	Phenolic glycosides	No	0.59	No	0.05	No	0.08

**CID 85244687**	15-[1-(4,5-dimethyl-6-oxo-3,6-dihydro-2H-pyran-2-yl) ethylidene]-5-hydroxy-10,14-dimethyl-9-oxo-3-oxapentacyclo$[9.7.0.0^{2,4}.0^{5,10}.0^{14,18}]$octadec-7-en-16-yl acetate		Steroid lactones	Yes	0.84	No	0.05	No	0.02
**CID 163054317**	15-[1-(3-hydroxy-4,5-dimethyl-6-oxo-3,6-dihydro-2H-pyran-2-yl)ethyl]-10,14-dimethyl-3-oxapentacyclo $[9.7.0.0^{2,4}.0^{5,10}.0^{14,18}]$octadec-7-en-9-one		Withanolides and derivatives	Yes	0.76	No	0.05	No	0.03
**CID 592382**	15-[1-(4,5-dimethyl-6-oxo-3,6-dihydro-2H-pyran-2-yl)-1-hydroxyethyl]-10,15-dihydroxy-2,16-dimethyl-8-oxapentacyclo$[9.7.0.0^{2,7}.0^{7,9}.0^{12,16}]$octadec-4-en-3-one		Withanolides and derivatives	Yes	0.88	No	0.05	No	0.04
**CID 12398**	heptadecane	Heptadekan	Alkanes	No	0.32	No	0.57	No	0.58
**CID 12389**	tetradecane	Tetradekan	Alkanes	No	0.18	No	0.57	No	0.58
**CID 162882387**	15-[1-(4,5-dimethyl-6-oxo-3,6-dihydro-2H-pyran-2-yl)-1-hydroxyethyl]-2,16-dimethyl-8-oxapentacyclo $[9.7.0.0^{2,7}.0^{7,9}.0^{12,16}]$octadec-4-ene-3,6-dione		Withanolides and derivatives	Yes	0.85	No	0.05	No	0.05
**CID 14236710**	15-[1-(4,5-dimethyl-6-oxo-3,6-dihydro-2H-pyran-2-yl)-1-hydroxyethyl]-5-hydroxy-10,14-dimethyl-3-oxapentacyclo $[9.7.0.0^{2,4}.0^{5,10}.0^{14,18}]$octadec-7-en-9-one	Withaniol	Withanolides and derivatives	Yes	0.87	No	0.05	No	0.04
**CID 162905720**	15-{1-[5-(hydroxymethyl)-4-methyl-6-oxo-3,6-dihydro-2H-pyran-2-yl]ethyl}-2,16-dimethyl-8-oxapentacyclo $[9.7.0.0^{2,7}.0^{7,9}.0^{12,16}]$octadecan-3-one		Withanolides and derivatives	Yes	0.74	No	0.05	No	0.06
**CID 580064**	6-hydroxy-15-{1-[5-(hydroxymethyl)-4-methyl-6-oxo-3,6-dihydro-2H-pyran-2-yl]ethyl}-2,16-dimethyl-8-oxapentacyclo$[9.7.0.0^{2,7}.0^{7,9}.0^{12,16}]$octadec-4-en-3-one	Withaferin A	Withanolides and derivatives	Yes	0.87	No	0.05	No	0.08
**CID 296119**	2-{[1-(5-ethyl-6-methylheptan-2-yl)-9a,11a-dimethyl-1H,2H,3H,3aH,3bH,4H,6H,7H,8H,9H,9aH,9bH,10H,11H,11aH-cyclopenta[a]phenanthren-7-yl]oxy}-6-(hydroxymethyl) oxane-3,4,5-triol	Bssg	Stigmastanes and derivatives	No	0.52	No	0.05	No	0.12
**CID 85149076**	6-(2,2-dimethyl-3-oxocyclopropoxy)-15-{1-[5-(hydroxymethyl)-4-methyl-6-oxo-3,6-dihydro-2H-pyran-2-yl]ethyl}-2,16-dimethyl-8-oxapentacyclo $[9.7.0.0^{2,7}.0^{7,9}.0^{12,16}]$octadecan-3-one		Withanolides and derivatives	Yes	0.79	No	0.05	No	0.07
**CID 1198**	1-hydroxypropane-1,2,3-tricarboxylic acid	Isocitric Acid	Tricarboxylic acids and derivatives	No	0.07	No	0.59	No	0.62
**CID 142770**	icos-11-enoic acid	Gondoic Acid	Long-chain fatty acids	No	0.00	No	0.33	No	0.32
**CID 5280343**	2-(3,4-dihydroxyphenyl)-3,5,7-trihydroxy-4H-chromen-4-one	Quercetin	Flavonols	No	0.51	No	0.05	No	0.06
**CID 73797121**	15-[1-(4,5-dimethyl-6-oxo-3,6-dihydro-2H-pyran-2-yl) ethyl]-6-hydroxy-2,16-dimethyl-8-oxapentacyclo $[9.7.0.0^{2,7}.0^{7,9}.0^{12,16}]$octadeca-4,14-dien-3-one		Withanolides and derivatives	Yes	0.76	No	0.05	No	0.06
**CID 162873960**	6-(1-{9a,9b,11a-trimethyl-7-oxo-1H,2H,3H,3aH,3bH,4H,5H,7H,9aH,9bH,10H,11H,11aH-cyclopenta[a]phenanthren-1-yl}ethyl)-3-(hydroxymethyl)-4-methyl-5,6-dihydro-2H-pyran-2-one		Withanolides and derivatives	No	0.60	No	0.05	No	0.09
**CID 3931**	octadeca-9,12-dienoic acid	Linolic Acid	Lineolic acids and derivatives	No	0.07	No	0.24	No	0.23

**CID 162962125**	6-[1-(5,9-dihydroxy-10,14-dimethyl-7-{[3,4,5-trihydroxy-6-(hydroxymethyl)oxan-2-yl]oxy}-3-oxapentacyclo $[9.7.0.0^{2,4}.0^{5,10}.0^{14,18}]$octadecan-15-yl)ethyl]-3,4-dimethyl-5,6-dihydro-2H-pyran-2-one		Withanolide glycosides and derivatives	Yes	0.72	No	0.05	No	0.04
**CID 8141**	nonane	Nonan	Alkanes	No	0.30	No	0.57	No	0.58
**CID 74039189**	15-[1-(4,5-dimethyl-6-oxooxan-2-yl)ethyl]-6-hydroxy-2,16-dimethyl-8-oxapentacyclo$[9.7.0.0^{2,7}.0^{7,9}.0^{12,16}]$ octadec-4-en-3-one		Withanolides and derivatives	Yes	0.76	No	0.06	No	0.10
**CID 72835871**	5,6-dihydroxy-15-{1-[5-(hydroxymethyl)-4-methyl-6-oxo-3,6-dihydro-2H-pyran-2-yl]ethyl}-2,16-dimethyl-8-oxapentacyclo$[9.7.0.0^{2,7}.0^{7,9}.0^{12,16}]$octadecan-3-one	Viscosalactone B	Other	Yes	0.91	No	0.06	No	0.07
**CID 3893**	dodecanoic acid	Vulvic Acid	Medium-chain fatty acids	No	0.08	No	0.51	No	0.43
**CID 163045000**	15-[1-(4,5-dimethyl-6-oxo-3,6-dihydro-2H-pyran-2-yl) ethyl]-6-hydroxy-2,11,16-trimethyl-8-oxapentacyclo $[9.7.0.0^{2,7}.0^{7,9}.0^{12,16}]$octadeca-4,14-dien-3-one		Withanolides and derivatives	Yes	0.76	No	0.06	No	0.02
**CID 163006961**	15-hydroxy-6-{1-[5-(hydroxymethyl)-4-methyl-6-oxo-3,6-dihydro-2H-pyran-2-yl]ethyl}-7,11-dimethyl-3-oxapentacyclo$[8.8.0.0^{2,4}.0^{2,7}.0^{11,16}]$octadec-13-en-12-one		Withanolides and derivatives	Yes	0.87	No	0.06	No	0.07
**CID 13743180**	6-(1-{3a-hydroxy-9a,11a-dimethyl-9-oxo-1H,2H,3H,3aH,3bH,4H,6H,9H,9aH,9bH,10H,11H,11aH-cyclopenta[a]phenanthren-1-yl}-1-hydroxyethyl)-3-(hydroxymethyl)-4-methyl-5,6-dihydro-2H-pyran-2-one		Withanolides and derivatives	Yes	0.74	No	0.06	No	0.05
**CID 74039186**	15-[1-(4,5-dimethyl-6-oxo-3,6-dihydro-2H-pyran-2-yl)-1-hydroxyethyl]-6,15-dihydroxy-2,16-dimethyl-8-oxapentacyclo$[9.7.0.0^{2,7}.0^{7,9}.0^{12,16}]$octadec-4-en-3-one		Withanolides and derivatives	Yes	0.88	No	0.06	No	0.08
**CID 162960476**	6-(1-{2,3b,10-trihydroxy-9a,11a-dimethyl-9-oxo-1H,2H,3H,3aH,3bH,4H,6H,9H,9aH,9bH,10H,11H,11aH-cyclopenta[a]phenanthren-1-yl}ethyl)-3,4-dimethyl-5,6-dihydro-2H-pyran-2-one (6-hydroxy-15-{1-[5-(hydroxymethyl)-4-methyl-6-oxo-3,6-		Withanolides and derivatives	Yes	0.78	No	0.06	No	0.05
**CID 45359808**	dihydro-2H-pyran-2-yl]ethyl}-2,16-dimethyl-3-oxo-8-oxapentacyclo$[9.7.0.0^{2,7}.0^{7,9}.0^{12,16}]$octadecan-5-yl) oxidanesulfonic acid 15-[1-(4,5-dimethyl-6-oxo-3,6-dihydro-2H-pyran-2-yl)-1-		Withanolides and derivatives	Yes	0.80	No	0.06	No	0.10
**CID 73823413**	hydroxyethyl]-15-hydroxy-2,16-dimethyl-3-oxo-8-oxapentacyclo$[9.7.0.0^{2,7}.0^{7,9}.0^{12,16}]$octadec-4-en-6-yl acetate 2-(3,4-dihydroxyphenyl)-3,5-dihydroxy-7-methoxy-4H-chromen-4-one		Withanolides and derivatives	Yes	0.84	No	0.06	No	0.07
**CID 5281691**	octadecanoic acid	Rhamnetin	Flavonols	Yes	0.72	No	0.06	No	0.07
**CID 5281**	2-aminopropanoic acid	Stearic Acid	Long-chain fatty acids	No	0.04	No	0.51	No	0.43
**CID 602**	15-hydroxy-N-[2-(5-{[3,4,5-trihydroxy-6-({[3,4,5-	L-Ala	Alanine and derivatives	No	0.10	Yes	0.70	No	0.66
**CID 72814196**	trihydroxy-6-(hydroxymethyl)oxan-2-yl]oxy}methyl)oxan-2-yl]oxy}-1H-indol-3-yl)ethyl]heptadecanamide heptadec-9-enoic acid		Phenolic glycosides	No	0.47	No	0.09	No	0.08
**CID 3014063**		heptadec-9-enoic acid	Long-chain fatty acids	No	0.05	No	0.33	No	0.32

**CID 73800706**	5,13-dihydroxy-15-{1-[5-(hydroxymethyl)-4-methyl-6-oxo-3,6-dihydro-2H-pyran-2-yl]ethyl}-10,14-dimethyl-3-oxapentacyclo[9.7.0.0^{2,4}.0^{5,10}.0^{14,18}]octadec-7-en-9-one	withastramonolide	Withanolides and derivatives	Yes	0.91	No	0.06	No	0.06
**CID 74039174**	6-(1-{1,6-dihydroxy-9a,11a-dimethyl-9-oxo-1H,2H,3H,4H,6H,9H,9aH,9bH,10H,11H,11aH-cyclopenta[a]phenanthren-1-yl}ethyl)-3,4-dimethyl-5,6-dihydro-2H-pyran-2-one		Withanolides and derivatives	No	0.64	No	0.06	No	0.01
**CID 76641674**	15-hydroxy-N-[2-(5-{[3,4,5-trihydroxy-6-({[3,4,5-trihydroxy-6-(hydroxymethyl)oxan-2-yl]oxy}methyl)oxan-2-yl]oxy}-1H-indol-3-yl)ethyl]hexadecanamide	Withanamide B	Phenolic glycosides	No	0.48	No	0.10	No	0.08
**CID 73091599**	5,6,7,8-tetrahydroxy-2-(4-hydroxyphenyl)-3-[(3,4,5-trihydroxy-6-{[(3,4,5-trihydroxy-6-methyloxan-2-yl)oxy]methyl}oxan-2-yl)oxy]-4H-chromen-4-one		Flavonoid-3-O-glycosides	No	0.31	No	0.02	No	0.03
**CID 11197**	tetracosanoic acid	Lignoceric Acid	Very long-chain fatty acids	No	0.09	No	0.51	No	0.43
**CID 76641673**	17-hydroxy-N-[2-(5-{[3,4,5-trihydroxy-6-({[3,4,5-trihydroxy-6-(hydroxymethyl)oxan-2-yl]oxy}methyl)oxan-2-yl]oxy}-1H-indol-3-yl)ethyl]octadecanamide	Withanamide D	Phenolic glycosides	No	0.48	No	0.10	No	0.08
**CID 301757**	15-[1-(4,5-dimethyl-6-oxo-3,6-dihydro-2H-pyran-2-yl)ethyl]-5,15-dihydroxy-10,14-dimethyl-3-oxapentacyclo[9.7.0.0^{2,4}.0^{5,10}.0^{14,18}]octadec-7-en-9-one	Withanone	Withanolides and derivatives	Yes	0.87	No	0.06	No	0.06
**CID 74024412**	6-(1-{9a,11a-dimethyl-7-oxo-1H,2H,3H,3aH,3bH,4H,5H,7H,9aH,9bH,10H,11H,11aH-cyclopenta[a]phenanthren-1-yl}ethyl)-3-(hydroxymethyl)-4-methyl-5,6-dihydro-2H-pyran-2-one	3-Oxowitha-1,4,24-Trienolide; (22R)-Form, 27-Hydroxy	Withanolides and derivatives	No	0.55	No	0.06	No	0.11
**CID 73797122**	5,15-dihydroxy-15-{1-[5-(hydroxymethyl)-4-methyl-6-oxo-3,6-dihydro-2H-pyran-2-yl]ethyl}-10,14-dimethyl-3-oxapentacyclo[9.7.0.0^{2,4}.0^{5,10}.0^{14,18}]octadec-7-en-9-one	27-Hydroxywithanone	Withanolides and derivatives	Yes	0.93	No	0.06	No	0.05
**CID 58427569**	4-{[3-(3,4-dihydroxyphenyl)prop-2-enoyl]oxy}-1,3,5-trihydroxycyclohexane-1-carboxylic acid		Quinic acids and derivatives	No	0.62	No	0.07	No	0.05
**CID 74021829**	16,17-dihydroxy-7-{1-[5-(hydroxymethyl)-4-methyl-6-oxo-3,6-dihydro-2H-pyran-2-yl]ethyl}-8,12-dimethyl-18-oxapentacyclo[13.2.1.0^{3,11}.0^{4,8}.0^{12,17}]octadecan-13-one		Other	Yes	0.91	No	0.07	No	0.05
**CID 329485**	6-(1-{1,3a,5,5a-tetrahydroxy-9a,11a-dimethyl-9-oxo-1H,2H,3H,3aH,3bH,4H,5H,5aH,6H,9H,9aH,9bH,10H,11H,11aH-cyclopenta[a]phenanthren-1-yl}-1-hydroxyethyl)-3,4-dimethyl-5,6-dihydro-2H-pyran-2-one		Withanolides and derivatives	Yes	0.80	No	0.07	No	0.08
**CID 14257**	undecane	undecane	Alkanes	No	0.21	No	0.57	No	0.58
**CID 76641671**	14-hydroxy-N-[2-(5-{[3,4,5-trihydroxy-6-({[3,4,5-trihydroxy-6-(hydroxymethyl)oxan-2-yl]oxy}methyl)oxan-2-yl]oxy}-1H-indol-3-yl)ethyl]hexadecanamide	Withanamide C	Phenolic glycosides	No	0.48	No	0.09	No	0.08
**CID 5255784**	6-(1-{7,9-dihydroxy-9a,11a-dimethyl-1H,2H,3H,3aH,3bH,4H,6H,7H,8H,9H,9aH,9bH,10H,11H,11aH-cyclopenta[a]phenanthren-1-yl}-1-hydroxyethyl)-3,4-dimethyl-5,6-dihydro-2H-pyran-2-one		Withanolides and derivatives	No	0.69	No	0.07	No	0.12
**CID 4602094**	octadec-7-enoic acid	octadec-7-enoic acid	Long-chain fatty acids	No	0.05	No	0.33	No	0.32
**CID 74039193**	3,4-dimethyl-6-(1-{5,7,9-trihydroxy-10,14-dimethyl-3-oxapentacyclo[9.7.0.0^{2,4}.0^{5,10}.0^{14,18}]octadecan-15-yl}ethyl)-5,6-dihydro-2H-pyran-2-one		Withanolides and derivatives	Yes	0.82	No	0.07	No	0.08

**CID 11635**	octadecane	Oktadekan	Alkanes	No	0.38	No	0.57	No	0.58
**CID 76641672**	16-hydroxy-N-[2-(5-{[3,4,5-trihydroxy-6-({[3,4,5-trihydroxy-6-(hydroxymethyl)oxan-2-yl]oxy}methyl)oxan-2-yl]oxy}-1H-indol-3-yl)ethyl]octadecanamide	Withanamide E	Phenolic glycosides	No	0.48	No	0.09	No	0.08
**CID 72764833**	16-hydroxy-N-[2-(5-{[3,4,5-trihydroxy-6-({[3,4,5-trihydroxy-6-(hydroxymethyl)oxan-2-yl]oxy}methyl)oxan-2-yl]oxy}-1H-indol-3-yl)ethyl]octadec-9-enamide	Withanamide F	Phenolic glycosides	No	0.50	No	0.07	No	0.10
**CID 163008986**	15-[1-(4,5-dimethyl-6-oxo-3,6-dihydro-2H-pyran-2-yl)ethyl]-1,5-dihydroxy-10,14-dimethyl-3-oxapentacyclo[9.7.0.0^{2,4}.0^{5,10}.0^{14,18}]octadec-7-en-9-one		Withanolides and derivatives	Yes	0.86	No	0.07	No	0.10
**CID 73190083**	5,6-dihydroxy-16-{1-[5-(hydroxymethyl)-4-methyl-6-oxo-3,6-dihydro-2H-pyran-2-yl]ethyl}-2,17-dimethyl-8,13-dioxahexacyclo[9.8.0.0^{2,7}.0^{7,9}.0^{12,14}.0^{12,17}]nonadecan-3-one		Withanolides and derivatives	No	0.94	No	0.07	No	0.09
**CID 163018694**	15-[1-(4,5-dimethyl-6-oxo-3,6-dihydro-2H-pyran-2-yl)ethyl]-15-hydroxy-10,14-dimethyl-3-oxapentacyclo[9.7.0.0^{2,4}.0^{5,10}.0^{14,18}]octadec-5-en-9-one		Withanolides and derivatives	Yes	0.79	No	0.08	No	0.11
**CID 119**	4-aminobutanoic acid	Gaba	Gamma amino acids and derivatives	No	0.14	No	0.62	No	0.46
**CID 4668**	hexadec-9-enoic acid	Zoomaric Acid	Long-chain fatty acids	No	0.07	No	0.33	No	0.32
**CID 77911828**	6-(1-{5-chloro-5a,6-dihydroxy-9a,11a-dimethyl-9-oxo-1H,2H,3H,3aH,3bH,4H,5H,5aH,6H,9H,9aH,9bH,10H,11H,11aH-cyclopenta[a]phenanthren-1-yl}ethyl)-3-(hydroxymethyl)-4-methyl-5,6-dihydro-2H-pyran-2-one		Withanolides and derivatives	Yes	0.80	No	0.08	No	0.02
**CID 73797123**	15-[1-(4,5-dimethyl-6-oxo-3,6-dihydro-2H-pyran-2-yl)ethyl]-5,7,15-trihydroxy-10,14-dimethyl-3-oxapentacyclo[9.7.0.0^{2,4}.0^{5,10}.0^{14,18}]octadecan-9-one		Withanolides and derivatives	Yes	0.90	No	0.08	No	0.08
**CID 8182**	dodecane	Dodekan	Alkanes	No	0.19	No	0.57	No	0.58
**CID 13942388**	2-(3,4-dihydroxyphenyl)-5-hydroxy-7-{[3,4,5-trihydroxy-6-(hydroxymethyl)oxan-2-yl]oxy}-3-[(3,4,5-trihydroxy-6-{[(3,4,5-trihydroxy-6-methyloxan-2-yl)oxy]methyl}oxan-2-yl)oxy]-4H-chromen-4-one		Flavonoid-7-O-glycosides	No	0.45	No	0.03	No	0.06
**CID 163016179**	6-(1-{5-[(5a,6-dihydroxy-1-{1-[5-(hydroxymethyl)-4-methyl-6-oxo-3,6-dihydro-2H-pyran-2-yl]ethyl}-9a,11a-dimethyl-9-oxo-1H,2H,3H,3aH,3bH,4H,5H,5aH,6H,9H,9aH,9bH,10H,11H,11aH-cyclopenta[a]phenanthren-5-yl)sulfanyl]-5a,6-dihydroxy-9a,11a-dimethyl-9-oxo-1H,2H,3H,3aH,3bH,4H,5H,5aH,6H,9H,9aH,9bH,10H,11H,11aH-cyclopenta[a]phenanthren-1-yl}ethyl)-3-(hydroxymethyl)-4-methyl-5,6-dihydro-2H-pyran-2-one	Ashwagandhanolide	Withanolides and derivatives	No	0.58	No	0.08	No	0.04
**CID 162851586**	6-(1-{3a,5a-dihydroxy-9a,11a-dimethyl-9-oxo-1H,2H,3H,3aH,5H,5aH,6H,9H,9aH,9bH,10H,11H,11aH-cyclopenta[a]phenanthren-1-yl}-1-hydroxyethyl)-3,4-dimethyl-5,6-dihydro-2H-pyran-2-one		Withanolides and derivatives	Yes	0.76	No	0.08	No	0.03
**CID 433363**	6-hydroxy-15-{1-[5-(hydroxymethyl)-4-methyl-6-oxo-3,6-dihydro-2H-pyran-2-yl]ethyl}-5-methoxy-2,16-dimethyl-8-oxapentacyclo[9.7.0.0^{2,7}.0^{7,9}.0^{12,16}]octadecan-3-one	Quresimine A	Withanolides and derivatives	Yes	0.74	No	0.08	No	0.08
**CID 12530**	tridecanoic acid	Tridecylic Acid	Long-chain fatty acids	No	0.10	No	0.51	No	0.43
**CID 11005**	tetradecanoic acid	Myristic Acid	Long-chain fatty acids	No	0.08	No	0.51	No	0.43

**CID 356**	octane	Octane	Alkanes	No	0.32	No	0.57	No	0.58
**CID 12442813**	1-(5-ethyl-6-methylheptan-2-yl)-9a,11a-dimethyl-hexadecahydro-1H-cyclopenta[a]phenanthren-7-one		Stigmastanes and derivatives	No	0.44	No	0.13	No	0.15
**CID 53462571**	2-(2-hydroxyethyl)-3,4-bis(hydroxymethyl)cyclopent-3-en-1-ol	Eucommiol	Iridoids and derivatives	No	0.48	No	0.28	No	0.30
**CID 301752**	6-(1-{3a-hydroxy-9a,11a-dimethyl-9-oxo-1H,2H,3H,3aH,3bH,4H,6H,9H,9aH,9bH,10H,11H,11aH-cyclopenta[a]phenanthren-1-yl}-1-hydroxyethyl)-3,4-dimethyl-5,6-dihydro-2H-pyran-2-one		Withanolides and derivatives	No	0.68	No	0.09	No	0.10
**CID 8215**	docosanoic acid {15-[1-(4,5-dimethyl-6-oxo-3,6-dihydro-2H-pyran-2-yl)	Behensaeure	Very long-chain fatty acids	No	0.07	No	0.51	No	0.43
**CID 73797125**	ethyl]-5,15-dihydroxy-10,14-dimethyl-9-oxo-3-oxapentacyclo[$9.7.0.0^{2,4}.0^{5,10}.0^{14,18}$]octadecan-7-yl} oxidanesulfonic acid icos-13-enoic acid		Withanolides and derivatives	Yes	0.83	No	0.09	No	0.10
**CID 4379731**		Paullinic Acid	Long-chain fatty acids	No	0.00	No	0.33	No	0.32
**CID 10470**	octacosanoic acid 15-[1-(4,5-dimethyl-6-oxo-3,6-dihydro-2H-pyran-2-yl)-1-	octacosanoic acid	Very long-chain fatty acids	No	0.20	No	0.51	No	0.43
**CID 74039187**	hydroxyethyl]-6,12-dihydroxy-2,16-dimethyl-8-oxapentacyclo[$9.7.0.0^{2,7}.0^{7,9}.0^{12,16}$]octadec-4-en-3-one octadec-11-enoic acid		Withanolides and derivatives	Yes	0.88	No	0.09	No	0.08
**CID 122325**	(6-{1-[6-(acetyloxy)-2,16-dimethyl-3-oxo-8-oxapentacyclo	Asclepic Acid	Long-chain fatty acids	No	0.05	No	0.33	No	0.32
**CID 73823412**	[$9.7.0.0^{2,7}.0^{7,9}.0^{12,16}$]octadec-4-en-15-yl]-1-hydroxyethyl}-4-methyl-2-oxo-5,6-dihydro-2H-pyran-3-yl)methyl acetate 6-[1-(4,5-dimethyl-6-oxo-3,6-dihydro-2H-pyran-2-yl)		Withanolides and derivatives	Yes	0.78	No	0.09	No	0.08
**CID 162980924**	ethyl]-1,9-dihydroxy-7,11-dimethyl-5-oxapentacyclo [$8.8.0.0^{2,7}.0^{4,6}.0^{11,16}$]octadeca-13,16-dien-12-one 15-[1-(4,5-dimethyl-6-oxo-3,6-dihydro-2H-pyran-2-yl)		Withanolides and derivatives	Yes	0.83	No	0.09	No	0.07
**CID 13831016**	ethyl]-5,15,18-trihydroxy-10,14-dimethyl-3-oxapentacyclo[$9.7.0.0^{2,4}.0^{5,10}.0^{14,18}$]octadec-7-en-9-one 6-(1-{3a-hydroxy-9a,11a-dimethyl-9-oxo-1H,2H,3H,3aH,		Withanolides and derivatives	Yes	0.90	No	0.09	No	0.08
**CID 162860542**	3bH,9H,9aH,9bH,10H,11H,11aH-cyclopenta[a]phenanthren-1-yl}-1-hydroxyethyl)-3,4-dimethyl-5,6-dihydro-2H-pyran-2-one 5,7-dihydroxy-2-(4-hydroxyphenyl)-4H-chromen-4-one		Withanolides and derivatives	No	0.67	No	0.09	No	0.07
**CID 5280443**	icosanoic acid	Apigenin	Flavones	No	0.53	No	0.10	No	0.08
**CID 10467**	16-hydroxy-N-[2-(5-({[3,4,5-trihydroxy-6-({[3,4,5-trihydroxy-6-(hydroxymethyl)oxan-2-yl]oxy}methyl)oxan-	Arachic Acid	Long-chain fatty acids	No	0.03	No	0.51	No	0.43
**CID 72973468**	2-yl]oxy}-1H-indol-3-yl)ethyl]heptadecanamide 6-(1-{5-chloro-5a,6-dihydroxy-9a,11a-dimethyl-9-oxo-1H,2H,3H,3aH,3bH,4H,5H,5aH,6H,9H,9aH,9bH,10H,11H,		Phenolic glycosides	No	0.49	No	0.10	No	0.08
**CID 162887735**	11aH-cyclopenta[a]phenanthren-1-yl}-1-hydroxyethyl)-3,4-dimethyl-5,6-dihydro-2H-pyran-2-one		Withanolides and derivatives	Yes	0.77	No	0.10	No	0.03

**CID 73823415**	6-(1-{3a-hydroxy-9a,11a-dimethyl-9-oxo-1H,2H,3H,3aH,3bH,4H,8H,9H,9aH,9bH,10H,11H,11aH-cyclopenta[a]phenanthren-1-yl}-1-hydroxyethyl)-3-(hydroxymethyl)-4-methyl-5,6-dihydro-2H-pyran-2-one		Withanolides and derivatives	Yes	0.76	No	0.10	No	0.06
**CID 73823410**	15-[1-(4,5-dimethyl-6-oxo-3,6-dihydro-2H-pyran-2-yl)-1-hydroxyethyl]-12-hydroxy-2,16-dimethyl-3-oxo-8-oxapentacyclo[9.7.0.0^{2,7}.0^{7,9}.0^{12,16}]octadec-4-en-6-yl acetate		Withanolides and derivatives	Yes	0.83	No	0.10	No	0.09
**CID 78157654**	6-(1-{3a-hydroxy-9a,11a-dimethyl-9-oxo-3H,3aH,3bH,4H,6H,9H,9aH,9bH,10H,11H,11aH-cyclopenta[a]phenanthren-1-yl}-1-hydroxyethyl)-3,4-dimethyl-5,6-dihydro-2H-pyran-2-one		Withanolides and derivatives	No	0.65	No	0.10	No	0.06
**CID 8222**	icosane	icosane	Alkanes	No	0.46	No	0.57	No	0.58
**CID 163020161**	5,15,18-trihydroxy-15-[1-(3-hydroxy-4,5-dimethyl-6-oxo-3,6-dihydro-2H-pyran-2-yl)ethyl]-10,14-dimethyl-3-oxapentacyclo[9.7.0.0^{2,4}.0^{5,10}.0^{14,18}]octadec-7-en-9-one		Withanolides and derivatives	Yes	0.90	No	0.10	No	0.06
**CID 163030448**	6-(1-{5a-ethoxy-1,3a,5-trihydroxy-9a,11a-dimethyl-9-oxo-1H,2H,3H,3aH,3bH,4H,5H,5aH,6H,9H,9aH,9bH,10H,11H,11aH-cyclopenta[a]phenanthren-1-yl}-1-hydroxyethyl)-3,4-dimethyl-5,6-dihydro-2H-pyran-2-one		Withanolides and derivatives	Yes	0.78	No	0.10	No	0.09
**CID 72973518**	18-hydroxy-N-[2-(5-{[3,4,5-trihydroxy-6-({[3,4,5-trihydroxy-6-(hydroxymethyl)oxan-2-yl]oxy}methyl)oxan-2-yl]oxy}-1H-indol-3-yl)ethyl]nonadecanamide		Phenolic glycosides	No	0.50	No	0.10	No	0.08
**CID 13849**	pentadecanoic acid	Pentadecylic Acid	Long-chain fatty acids	No	0.08	No	0.51	No	0.43
**CID 206**	6-(hydroxymethyl)oxane-2,3,4,5-tetrol	D-Glc	Hexoses	No	0.44	No	0.04	No	0.08
**CID 379**	octanoic acid	Octansaeure	Medium-chain fatty acids	No	0.19	No	0.51	No	0.43
**CID 72755035**	17-hydroxy-N-[2-(5-{[3,4,5-trihydroxy-6-({[3,4,5-trihydroxy-6-(hydroxymethyl)oxan-2-yl]oxy}methyl)oxan-2-yl]oxy}-1H-indol-3-yl)ethyl]nonadecanamide		Phenolic glycosides	No	0.44	No	0.09	No	0.08
**CID 17085**	tricosanoic acid	tricosanoic acid	Very long-chain fatty acids	No	0.09	No	0.51	No	0.43
**CID 10469**	hexacosanoic acid	hexacosanoic acid	Very long-chain fatty acids	No	0.16	No	0.51	No	0.43
**CID 3208**	icosa-11,14-dienoic acid	icosa-11,14-dienoic acid	Long-chain fatty acids	No	0.06	No	0.24	No	0.23
**CID 72739516**	1-(5-ethyl-6-methylhept-3-en-2-yl)-9a,11a-dimethyl-1H,2H,3H,3aH,3bH,4H,6H,7H,8H,9H,9aH,9bH,10H,11H,11aH-cyclopenta[a]phenanthren-7-one		Stigmastanes and derivatives	No	0.38	No	0.05	No	0.08
**CID 86821**	1-(5-ethyl-6-methylheptan-2-yl)-9a,11a-dimethyl-1H,2H,3H,3aH,3bH,4H,6H,7H,8H,9H,9aH,9bH,10H,11H,11aH-cyclopenta[a]phenanthren-7-ol	Cupreol	Stigmastanes and derivatives	No	0.55	No	0.10	No	0.13
**CID 12391**	pentadecane	Pentadekan	Alkanes	No	0.18	No	0.57	No	0.58
**CID 13743182**	6-(1-{3a-hydroxy-9a,11a-dimethyl-9-oxo-1H,2H,3H,3aH,3bH,4H,8H,9H,9aH,9bH,10H,11H,11aH-cyclopenta[a]phenanthren-1-yl}-1-hydroxyethyl)-3,4-dimethyl-5,6-dihydro-2H-pyran-2-one		Withanolides and derivatives	No	0.69	No	0.11	No	0.06

**CID 163071135**	6-(1-{3a-hydroxy-9a,11a-dimethyl-9-oxo-1H,2H,3H,3aH,3bH,4H,5H,9H,9aH,9bH,10H,11H,11aH-cyclopenta[a]phenanthren-1-yl}-1-hydroxyethyl)-3,4-dimethyl-5,6-dihydro-2H-pyran-2-one		Withanolides and derivatives	No	0.68	No	0.12	No	0.12
**CID 162892340**	6-(1-{3a,6-dihydroxy-9a,11a-dimethyl-9-oxo-1H,2H,3H,3aH,3bH,4H,6H,9H,9aH,9bH,10H,11H,11aH-cyclopenta[a]phenanthren-1-yl}-1-hydroxyethyl)-3,4-dimethyl-5,6-dihydro-2H-pyran-2-one		Withanolides and derivatives	Yes	0.73	No	0.12	No	0.09
**CID 13743195**	6-(1-{5-chloro-1,3a,5a-trihydroxy-9a,11a-dimethyl-9-oxo-1H,2H,3H,3aH,3bH,4H,5H,5aH,6H,9H,9aH,9bH,10H,11H,11aH-cyclopenta[a]phenanthren-1-yl}-1-hydroxyethyl)-3,4-dimethyl-5,6-dihydro-2H-pyran-2-one		Withanolides and derivatives	Yes	0.81	No	0.12	No	0.03
**CID 13743184**	6-(1-{1,3a-dihydroxy-9a,11a-dimethyl-9-oxo-1H,2H,3H,3aH,3bH,4H,6H,9H,9aH,9bH,10H,11H,11aH-cyclopenta[a]phenanthren-1-yl}-1-hydroxyethyl)-3,4-dimethyl-5,6-dihydro-2H-pyran-2-one		Withanolides and derivatives	Yes	0.73	No	0.12	No	0.13
**CID 3372729**	15-[1-(4,5-dimethyl-6-oxo-3,6-dihydro-2H-pyran-2-yl)-1-hydroxyethyl]-12,15-dihydroxy-2,16-dimethyl-8-oxapentacyclo[9.7.0.0^{2,7}.0^{7,9}.0^{12,16}]octadec-4-en-3-one		Withanolides and derivatives	Yes	0.87	No	0.13	No	0.09
**CID 162920570**	6-(1-{5-ethoxy-1,3a,5a-trihydroxy-9a,11a-dimethyl-9-oxo-1H,2H,3H,3aH,3bH,4H,5H,5aH,6H,9H,9aH,9bH,10H,11H,11aH-cyclopenta[a]phenanthren-1-yl}-1-hydroxyethyl)-3,4-dimethyl-5,6-dihydro-2H-pyran-2-one		Withanolides and derivatives	Yes	0.77	No	0.14	No	0.10
**CID 709**	3-(4-hydroxy-3-methoxyphenyl)prop-2-enoic acid	Ferulic Acid	Hydroxycinnamic acids	Yes	0.79	No	0.14	No	0.10
**CID 5280460**	7-hydroxy-6-methoxy-2H-chromen-2-one	Buxuletin	7-hydroxycoumarins	No	0.69	No	0.14	No	0.22
**CID 162967639**	6-(1-{1,3a-dihydroxy-9a,11a-dimethyl-9-oxo-1H,2H,3H,3aH,3bH,4H,5H,9H,9aH,9bH,10H,11H,11aH-cyclopenta[a]phenanthren-1-yl}-1-hydroxyethyl)-3,4-dimethyl-5,6-dihydro-2H-pyran-2-one		Withanolides and derivatives	Yes	0.73	No	0.14	No	0.14
**CID 442783**	5-hydroxy-1,7-bis(4-hydroxy-3-methoxyphenyl)hepta-1,4,6-trien-3-one	Curcumin	Curcuminoids	Yes	0.84	No	0.18	No	0.11
**CID 163001934**	3-(tetratriacont-3-en-1-yl)-1,4-dioxane-2,5-dione	3-(tetratriacont-3-en-1-yl)-1,4-dioxane-2,5-dione	1,4-dioxanes	No	0.67	No	0.19	No	0.11
**CID 8180**	undecanoic acid	undecanoic acid	Medium-chain fatty acids	No	0.12	No	0.51	No	0.43
**CID 738**	2-amino-4-carbamoylbutanoic acid	Hgln	Alpha amino acids	No	0.05	No	0.59	No	0.44
**CID 5281416**	6,7-dihydroxy-2H-chromen-2-one	Esculetin	6,7-dihydroxycoumarins	No	0.51	No	0.21	No	0.25
**CID 13743197**	6-(1-{5a-chloro-1,3a,5-trihydroxy-9a,11a-dimethyl-9-oxo-1H,2H,3H,3aH,3bH,4H,5H,5aH,6H,9H,9aH,9bH,10H,11H,11aH-cyclopenta[a]phenanthren-1-yl}-1-hydroxyethyl)-3,4-dimethyl-5,6-dihydro-2H-pyran-2-one		Withanolides and derivatives	Yes	0.73	No	0.21	No	0.18
**CID 73152883**	2-[1-[3-[3,4-dihydroxy-6-(hydroxymethyl)-5-[3,4,5-trihydroxy-6-[[3,4,5-trihydroxy-6-(hydroxymethyl)oxan-2-yl]oxymethyl]oxan-2-yl]oxyoxan-2-yl]oxy-1-hydroxy-10,13-dimethyl-2,3,4,7,8,9,11,12,14,15,16,17-dodecahydro-1H-cyclopenta[a]phenanthren-17-yl]ethyl]-5-(hydroxymethyl)-4-methyl-2,3-dihydropyran-6-one		Other	No	0.54	No	0.21	No	0.01

**CID 443011**	8-methyl-8-azabicyclo[3.2.1]octan-3-yl 2-methylbut-2-enoate	Tigloidin	Tropane alkaloids	No	0.61	No	0.28	No	0.30
**CID 1183**	4-hydroxy-3-methoxybenzaldehyde	Vaniline	Methoxyphenols	No	0.58	No	0.31	No	0.38
**CID 72960154**	15-hydroxy-N-[2-(5-{[3,4,5-trihydroxy-6-({[3,4,5-trihydroxy-6-(hydroxymethyl)oxan-2-yl]oxy}methyl)oxan-2-yl]oxy}-1H-indol-3-yl)ethyl]hexadec-9-enamide		Phenolic glycosides	No	0.48	No	0.05	No	0.11
**CID 965**	octadec-9-enoic acid	Oleic Acid	Long-chain fatty acids	No	0.05	No	0.33	No	0.32
**CID 189702**	[3,4,5-trihydroxy-6-[[2-[1-(6-hydroxy-2,16-dimethyl-3-oxo-8-oxapentacyclo[9.7.0.02,7.07,9.012,16]octadec-4-en-15-yl)ethyl]-4-methyl-6-oxo-2,3-dihydropyran-5-yl] methoxy]oxan-2-yl]methyl hexadecanoate		Other	No	0.58	No	0.32	No	0.09
**CID 8468**	4-hydroxy-3-methoxybenzoic acid	Vanillic Acid	M-methoxybenzoic acids and derivatives	Yes	0.72	No	0.43	No	0.25
**CID 307185**	6-(hydroxymethyl)thiane-2,3,4,5-tetrol	6-(hydroxymethyl) thiane-2,3,4,5-tetrol	Thianes	No	0.51	No	0.47	No	0.35
**CID 543360**	tetradec-7-enoic acid	tetradec-7-enoic acid	Long-chain fatty acids	No	0.05	No	0.33	No	0.32
**CID 72982306**	17-hydroxy-N-[2-(5-{[3,4,5-trihydroxy-6-({[3,4,5-trihydroxy-6-(hydroxymethyl)oxan-2-yl]oxy}methyl)oxan-2-yl]oxy}-1H-indol-3-yl)ethyl]octadeca-6,9-dienamide		Phenolic glycosides	No	0.49	No	0.04	No	0.10
**CID 163113507**	2-[1-hydroxy-1-[8-hydroxy-13-(hydroxymethyl)-10-methyl-1-oxo-7,9,11,12,14,15,16,17-octahydro-4H-cyclopenta[a]phenanthren-17-yl]ethyl]-4,5-dimethyl-2,3-dihydropyran-6-one		Other	Yes	0.74	No	0.48	No	0.02
**CID 441070**	1,3-bis(1-methylpyrrolidin-2-yl)propan-2-one	Cuskhygrine	Alkaloids and derivatives	No	0.62	No	0.49	No	0.12
**CID 370**	3,4,5-trihydroxybenzoic acid	Gallic Acid	Gallic acids	No	0.65	No	0.50	No	0.42
**CID 74978174**	2-(3,4-dihydroxyphenyl)-5-hydroxy-3-{[3,4,5-trihydroxy-6-(hydroxymethyl)oxan-2-yl]oxy}-7-[(3,4,5-trihydroxy-6-{[(3,4,5-trihydroxy-6-methyloxan-2-yl)oxy]methyl}oxan-2-yl)oxy]-4H-chromen-4-one	Quercetin 3-Glucoside-7-Rutinoside	Flavonoid-7-O-glycosides	No	0.45	No	0.03	No	0.06
**CID 425265**	1,3-bis(piperidin-2-yl)propan-2-one	Anapheline	Piperidines	No	0.57	No	0.52	No	0.20
**CID 10742**	4-hydroxy-3,5-dimethoxybenzoic acid	Cedar Acid	Gallic acid and derivatives	No	0.69	No	0.52	No	0.28
**CID 442877**	3-phenyl-4H,5H,6H-pyrrolo[1,2-b]pyrazole	Withasomnine	Phenylpyrazoles	No	0.59	No	0.56	No	0.54
**CID 12535**	triacontane	triacontane	Alkanes	No	0.58	No	0.57	No	0.58
**CID 12407**	hexacosane	hexacosane	Alkanes	No	0.58	No	0.57	No	0.58
**CID 12409**	nonacosane	nonacosane	Alkanes	No	0.58	No	0.57	No	0.58
**CID 12410**	hentriacontane	Hentriacontan	Alkanes	No	0.58	No	0.57	No	0.58
**CID 11636**	heptacosane	heptacosane	Alkanes	No	0.58	No	0.57	No	0.58
**CID 12408**	octacosane	octacosane	Alkanes	No	0.58	No	0.57	No	0.58
**CID 12406**	pentacosane	pentacosane	Alkanes	No	0.56	No	0.57	No	0.58
**CID 138925**	octatetracontane	octatetracontane	Alkanes	No	0.56	No	0.57	No	0.58
**CID 81613**	hentetracontane	hentetracontane	Alkanes	No	0.54	No	0.57	No	0.58
**CID 26519**	tetratriacontane	tetratriacontane	Alkanes	No	0.54	No	0.57	No	0.58

**CID 23494**	tetratetracontane	tetratetracontane	Alkanes	No	0.54	No	0.57	No	0.58
**CID 522398**	tritetracontane	tritetracontane	Alkanes	No	0.54	No	0.57	No	0.58
**CID 522399**	hexatetracontane	hexatetracontane	Alkanes	No	0.54	No	0.57	No	0.58
**CID 23598**	heptatriacontane	heptatriacontane	Alkanes	No	0.54	No	0.57	No	0.58
**CID 114842**	nonatriacontane	nonatriacontane	Alkanes	No	0.54	No	0.57	No	0.58
**CID 12413**	pentatriacontane	pentatriacontane	Alkanes	No	0.54	No	0.57	No	0.58
**CID 1115**	2-{[3,4-dihydroxy-2,5-bis(hydroxymethyl)oxolan-2-yl]oxy}-6-(hydroxymethyl)oxane-3,4,5-triol	Sucrose	O-glycosyl compounds	No	0.44	No	0.06	No	0.13
**CID 20149**	tetracontane	tetracontane	Alkanes	No	0.54	No	0.57	No	0.58
**CID 11006**	hexadecane	Zetan	Alkanes	No	0.33	No	0.57	No	0.58
**CID 236**	2-amino-3-carbamoylpropanoic acid	Hasp	Asparagine and derivatives	No	0.05	No	0.57	No	0.43
**CID 15600**	decane	Decane	Alkanes	No	0.30	No	0.57	No	0.58
**CID 247705**	1-(5,6-dimethylhept-3-en-2-yl)-9a,11a-dimethyl-1H,2H,3H,3aH,6H,7H,8H,9H,9aH,9bH,10H,11H,11aH-cyclopenta[a]phenanthren-7-ol	Ergosterol	Ergosterols and derivatives	No	0.34	No	0.08	No	0.09
**CID 8216**	docos-13-enoic acid	Erucic Acid	Very long-chain fatty acids	No	0.03	No	0.33	No	0.32
**CID 1064**	1,3,4,5-tetrahydroxycyclohexane-1-carboxylic acid	Kinic Acid	Quinic acids and derivatives	No	0.45	No	0.10	No	0.12
**CID 162949541**	6-[1-(9-hydroxy-9a,11a-dimethyl-7-{[3,4,5-trihydroxy-6-({[3,4,5-trihydroxy-6-(hydroxymethyl)oxan-2-yl]oxy}methyl)oxan-2-yl]oxy}-1H,2H,3H,3aH,3bH,4H,6H,7H,8H,9H,9aH,9bH,10H,11H,11aH-cyclopenta[a]phenanthren-1-yl)ethyl]-4-methyl-3-({[3,4,5-trihydroxy-6-({[3,4,5-trihydroxy-6-(hydroxymethyl)oxan-2-yl]oxy}methyl)oxan-2-yl]oxy}methyl)-5,6-dihydro-2H-pyran-2-one	Withanoside Ix	Withanolide glycosides and derivatives	No	0.46	No	0.03	No	0.03
**CID 11008**	dotriacontane	dotriacontane	Alkanes	No	0.54	No	0.57	No	0.58
**CID 12411**	tritriacontane	Triacontane	Alkanes	No	0.54	No	0.57	No	0.58
**CID 12412**	hexatriacontane	hexatriacontane	Alkanes	No	0.54	No	0.57	No	0.58
**CID 8158**	nonanoic acid	Pelargon	Medium-chain fatty acids	No	0.20	No	0.51	No	0.43
**CID 23599**	octatriacontane	octatriacontane	Alkanes	No	0.54	No	0.57	No	0.58
**CID 123244**	dotetracontane	dotetracontane	Alkanes	No	0.54	No	0.57	No	0.58
**CID 91127917**	dotriacont-8-enoic acid	8-Dotriacontenoic Acid	Very long-chain fatty acids	No	0.20	No	0.33	No	0.32
**CID 12403**	henicosane	henicosane	Alkanes	No	0.47	No	0.57	No	0.58
**CID 12592**	tetracosane	tetracosane	Alkanes	No	0.53	No	0.57	No	0.58
**CID 5282598**	octatriacontanoic acid	octatriacontanoic acid	Other	No	0.18	No	0.51	No	0.43
**CID 53679925**	nonadeca-9,12-dienoic acid	nonadeca-9,12-dienoic acid	Long-chain fatty acids	No	0.02	No	0.24	No	0.23
**CID 73836089**	hexacos-11-enoic acid	hexacos-11-enoic acid	Very long-chain fatty acids	No	0.14	No	0.33	No	0.32

**CID 73721023**	pentacos-19-enoic acid	pentacos-19-enoic acid	Very long-chain fatty acids	No	0.11	No	0.33	No	0.32
**CID 12405**	docosane	Dokosan	Alkanes	No	0.51	No	0.57	No	0.58
**CID 3693124**	8-azabicyclo[3.2.1]octane-1,2,3,4-tetrol	8-azabicyclo[3.2.1] octane-1,2,3,4-tetrol	Tropane alkaloids	No	0.45	No	0.54	No	0.07
**CID 72444876**	icosa-10,13-dienoic acid	icosa-10,13-dienoic acid	Long-chain fatty acids	No	0.04	No	0.24	No	0.23
**CID 72728218**	octacos-21-enoic acid	octacos-21-enoic acid	Very long-chain fatty acids	No	0.18	No	0.33	No	0.32
**CID 12534**	tricosane	tricosane	Alkanes	No	0.51	No	0.57	No	0.58
**CID 16898**	henicosanoic acid	henicosanoic acid	Long-chain fatty acids	No	0.05	No	0.51	No	0.43
**CID 440765**	2-amino-3-seleninopropanoic acid	2-amino-3-seleninopropanoic acid	Alpha amino acids	No	0.16	No	0.51	No	0.39
**CID 78383961**	pentacos-17-enoic acid	pentacos-17-enoic acid	Very long-chain fatty acids	No	0.11	No	0.33	No	0.32
**CID 195555**	hexacos-18-enoic acid	hexacos-18-enoic acid	Other	No	0.14	No	0.33	No	0.32
**CID 72955969**	17-hydroxy-N-[2-(5-{[3,4,5-trihydroxy-6-({[3,4,5-trihydroxy-6-({[3,4,5-trihydroxy-6-(hydroxymethyl)oxan-2-yl]oxy}methyl)oxan-2-yl]oxy}methyl)oxan-2-yl]oxy}-1H-indol-3-yl)ethyl]octadeca-6,9-dienamide	Withanamide I	Oligosaccharides	No	0.43	No	0.04	No	0.10
**CID 5282597**	heptatriacontanoic acid	heptatriacontanoic acid	Very long-chain fatty acids	No	0.18	No	0.51	No	0.43
**CID 3014053**	pentadec-9-enoic acid	pentadec-9-enoic acid	Long-chain fatty acids	No	0.07	No	0.33	No	0.32
**CID 53393964**	icosa-8,14-dienoic acid	icosa-8,14-dienoic acid	Long-chain fatty acids	No	0.02	No	0.33	No	0.32
**CID 78383960**	tricos-17-enoic acid	tricos-17-enoic acid	Very long-chain fatty acids	No	0.07	No	0.33	No	0.32
**CID 53802661**	heptadec-7-enoic acid	heptadec-7-enoic acid	Long-chain fatty acids	No	0.05	No	0.33	No	0.32
**CID 3851514**	8-azabicyclo[3.2.1]octane-1,2,3,4,6-pentol		Tropane alkaloids	No	0.47	No	0.54	No	0.08
**CID 53806273**	nonadeca-10,13-dienoic acid	10Z,13Z-Nonadecadienoic Acid	Long-chain fatty acids	No	0.04	No	0.24	No	0.23
**CID 71321501**	triacont-23-enoic acid	triacont-23-enoic acid	Other	No	0.20	No	0.33	No	0.32
**CID 53813767**	nonadec-13-enoic acid	nonadec-13-enoic acid	Long-chain fatty acids	No	0.00	No	0.33	No	0.32
**CID 2969**	decanoic acid	Decanoate	Medium-chain fatty acids	No	0.14	No	0.51	No	0.43
**CID 12306778**	1-(1-methylpyrrolidin-2-yl)-3-(piperidin-2-yl)propan-2-one	Anahygrine	Alkaloids and derivatives	No	0.68	No	0.57	No	0.47
**CID 4385123**	heptadec-10-enoic acid	Cis-10-Heptadecenoic Acid	Long-chain fatty acids	No	0.05	No	0.33	No	0.32
**CID 150284721**	docosa-11,14-dienoic acid	docosa-11,14-dienoic acid	Very long-chain fatty acids	No	0.09	No	0.24	No	0.23
**CID 2153**	1,3-dimethyl-2,3,6,9-tetrahydro-1H-purine-2,6-dione	Theophyllin	Xanthines	No	0.62	No	0.62	Yes	0.85
**CID 71404199**	docosa-7,13-dienoic acid	docosa-7,13-dienoic acid	Other	No	0.03	No	0.33	No	0.32
**CID 71374575**	octacos-9-enoic acid	octacos-9-enoic acid	Very long-chain fatty acids	No	0.18	No	0.33	No	0.32
**CID 181572**	tritriacontanoic acid	tritriacontanoic acid	Very long-chain fatty acids	No	0.20	No	0.51	No	0.43

**CID 73836094**	triaconta-9,23-dienoic acid	triaconta-9,23-dienoic acid	Other	No	0.28	No	0.33	No	0.32
**CID 193435**	docosa-13,16-dienoic acid	docosa-13,16-dienoic acid	Very long-chain fatty acids	No	0.09	No	0.24	No	0.23
**CID 5282595**	pentatriacontanoic acid	pentatriacontanoic acid	Other	No	0.20	No	0.51	No	0.43
**CID 53904386**	hexadec-10-enoic acid	hexadec-10-enoic acid	Long-chain fatty acids	No	0.07	No	0.33	No	0.32
**CID 71365436**	pentadec-7-enoic acid	pentadec-7-enoic acid	Long-chain fatty acids	No	0.07	No	0.33	No	0.32
**CID 8424**	8-methyl-8-azabicyclo[3.2.1]octan-3-ol	Tropine	Tropane alkaloids	No	0.54	Yes	0.72	Yes	0.91
**CID 86195978**	triacont-24-enoic acid	24-triacontenoic acid; (z) -form	Other	No	0.20	No	0.12	No	0.06
**CID 71343200**	heptacos-20-enoic acid	heptacos-20-enoic acid	Very long-chain fatty acids	No	0.16	No	0.33	No	0.32
**CID 19255**	dotriacontanoic acid	dotriacontanoic acid	Very long-chain fatty acids	No	0.20	No	0.51	No	0.43
